# Defect‐Templated Phase Engineering in Atomically Thin Metals

**DOI:** 10.1002/adma.74689

**Published:** 2026-08-19

**Authors:** Arpit Jain, Boyang Zheng, Sawani Datta, Kanchan Ulman, Jakob Henz, Matthew Wei‐Jun Liu, Van Dong Pham, Wen He, Chengye Dong, Li‐Syuan Lu, Alexander Vera, Nader Sawtarie, Wesley Auker, Ke Wang, Bob Hengstebeck, Zachary W. Henshaw, Shreya Mathela, Maxwell Wetherington, William H. Blades, Kenneth Knappenberger, Ursula Wurstbauer, Su Ying Quek, Ulrich Starke, Shengxi Huang, Vincent H. Crespi, Joshua A. Robinson

**Affiliations:** ^1^ Department of Materials Science and Engineering The Pennsylvania State University University Park Pennsylvania USA; ^2^ Department of Physics The Pennsylvania State University University Park Pennsylvania USA; ^3^ 2‐Dimensional Crystal Consortium The Pennsylvania State University University Park Pennsylvania USA; ^4^ Max‐Planck‐Institut Für Festkörperforschung Stuttgart Germany; ^5^ Department of Physics National University of Singapore Singapore Singapore; ^6^ Institute of Physics and Center for Soft Nanoscience (SoN) University of Münster Münster Germany; ^7^ Paul‐Drude‐Institut Für Festkörperelektronik Leibniz‐Institut Im Forschungsverbund Berlin e. V. Berlin Germany; ^8^ Department of Materials Science and Engineering National University of Singapore Singapore Singapore; ^9^ Center For 2‐Dimensional and Layered Materials The Pennsylvania State University University Park Pennsylvania USA; ^10^ Department of Chemical and Petroleum Engineering University of Pittsburgh Pittsburgh Pennsylvania USA; ^11^ Materials Research Institute The Pennsylvania State University University Park Pennsylvania USA; ^12^ Department of Physics and Engineering Physics Juniata College Huntingdon Pennsylvania USA; ^13^ Department of Chemistry The Pennsylvania State University University Park Pennsylvania USA; ^14^ Centre For Advanced 2D Materials National University of Singapore Singapore Singapore; ^15^ NUS Graduate School, Integrative Sciences and Engineering Programme National University of Singapore Singapore Singapore; ^16^ Department of Electrical and Computer Engineering and the Rice Advanced Materials Institute Rice University Houston Texas USA; ^17^ Center For Atomically Thin Multifunctional Coatings The Pennsylvania State University University Park Pennsylvania USA

## Abstract

Achieving deterministic control over crystal phase at the atomic limit remains a fundamental challenge for atomically thin metals, where subtle differences in atomic registry can produce large changes in electronic and optical functionality. Here, we establish interfacial defect templating as a general materials‐design strategy for phase engineering in confined two‐dimensional metals, using monolayer silver as a model system. By tailoring the defect chemistry of a graphene overlayer, we selectively stabilize two competing crystalline phases of two‐dimensional Ag at the graphene/SiC interface: a near‐commensurate phase promoted by vacancy and line defects in epitaxial graphene, and a denser phase favored beneath intrinsically sp^3^‐rich zero‐layer graphene. Multimodal characterization reveals distinct lattice registries, electronic structures, and charge transfer to the graphene overlayer for each phase. First‐principles calculations show that phase selectivity arises from a competition between kinetically favored nucleation pathways and thermodynamically preferred packing configurations, explaining both controlled phase formation and long‐term evolution. The defect‐programmed Ag phases exhibit strongly contrasting linear and nonlinear optical responses, enabling phase‐tunable optical functionality at atomic thickness. More broadly, this work reframes defects as deliberate design elements for programming structure–property relationships in confined two‐dimensional metals.

## Graphical abstract

Graphene defects are transformed from passive imperfections into programmable templates for phase‐selective growth of atomically thin silver. Plasma‐generated boundary defects favor Ag_(1)_, whereas sp^3^‐rich zero‐layer graphene promotes Ag_(2)_. This defect‐directed intercalation links local graphene chemistry to crystalline phase, electronic structure, and optical functionality, establishing a transferable strategy for the growth of confined two‐dimensional metals.

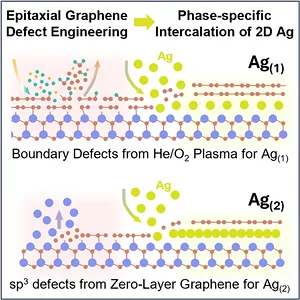

## 1 Introduction

Two‐dimensional materials constitute an emerging class of quantum‐confined materials whose electronic and optical properties differ markedly from their bulk counterparts [1, 2]. At the atomic thickness limit, modified bonding environments, enhanced light–matter interaction, and strong coupling to supporting substrates render their properties highly sensitive to crystal structure [3, 4]. Consequently, even modest variations in lattice registry or interfacial chemistry can lead to pronounced changes in functionality. Most existing approaches to structural control in two‐dimensional (2D) systems rely on indirect tuning of growth parameters—such as temperature, flux, or ambient [5, 6, 7, 8]—which often exhibit limited transferability and mechanistic specificity across different materials systems and confinement geometries. An alternative approach is to leverage defects. Although defects have traditionally been viewed as sources of disorder, they are increasingly recognized as active elements that influence nucleation pathways, interfacial energetics, and phase stability [9, 10]. In confined heterostructures, defects can mediate access to buried interfaces, modify local bonding configurations, and stabilize distinct structural motifs [11, 12, 13, 14], motivating a materials‐design framework in which defects act as tunable templates for controlling structure and functionality.

Intercalated two‐dimensional metals offer a particularly useful platform for exploring this framework. Intercalation stabilizes metal atoms within a host material, enabling access to atomically thin metallic phases that are difficult to realize using conventional evaporative [15, 16, 17] or sputtering‐based [18, 19, 20] techniques. At the epitaxial graphene (EG)/SiC interface [21, 22, 23], intercalation has enabled the formation of monolayer allotropes of a range of metals [24, 25, 26, 27, 28, 29], including noble metals [8, 30, 31, 32, 33, 34, 35, 36, 37, 38], while simultaneously decoupling graphene from the substrate and modifying its electronic structure [39]. More recently, confinement heteroepitaxy (CHet), which exploits defect‐mediated access to buried interfaces under near‐ambient conditions, has facilitated large‐area coalescence of intercalated 2D metals 40]. In these confined systems, variations in intercalation pathways and local interfacial environments can lead to multiple competing structural phases [25, 41, 42, 43], highlighting both the opportunity and complexity associated with phase engineering in confined metallic systems.

Within this broader context, silver (Ag) confined at the graphene/SiC interface serves as a well‐studied and informative model system. Atomically thin Ag has attracted interest due to its plasmonic response and potential relevance for optical and optoelectronic applications [16, 44, 45]; however, realizing these properties critically depends on controlling its structural phase [46, 47]. Both monolayer and few‐layer 2D Ag phases have been synthesized on SiC using plasma‐assisted [22, 48] and plasma‐free [33] intercalation methods, revealing at least two distinct structures: a near‐commensurate triangular (1 × 1) lattice [33] with an indirect bandgap of ∼ 1.1 eV [49] (Ag_(1)_) and a denser phase (Ag_(2)_) characterized by a distinct ultralow‐frequency Raman signature [50]. Prior studies have shown that the electronic and vibrational properties of the EG/Ag/SiC heterostructure depend on both the Ag phase and the underlying SiC stacking order [51], highlighting the importance of controlling phase formation. Despite these advances, a deterministic and reproducible route for selecting between Ag_(1)_ and Ag_(2)_ during growth has remained elusive.

Here, we demonstrate that this challenge can be overcome by treating the graphene overlayer as an active, defect‐programmable template rather than a passive confinement layer. We show that the defect chemistry of graphene deterministically selects between competing crystalline phases of monolayer Ag. By tailoring graphene growth conditions and defect motifs prior to intercalation within the CHet framework, we achieve near‐complete phase selectivity. The denser Ag_(2)_ phase preferentially forms beneath sp^3^‐rich zero‐layer graphene (ZLG), adopting a (3√3 × 3√3)R30° lattice commensurate with a 5 × 5 SiC unit cell, as confirmed by cross‐sectional scanning transmission electron microscopy (TEM), low‐energy electron diffraction (LEED), and Auger electron spectroscopy (AES) mapping. In contrast, the near‐commensurate Ag_(1)_ phase is stabilized beneath vacancy‐ and line‐defect motifs introduced into monolayer epitaxial graphene via helium plasma treatment, highlighting the role of distinct defect pathways in mediating access to the buried Ag/SiC interface and templating phase formation.

First‐principles density functional theory calculations reveal that Ag_(2)_ is thermodynamically favored, consistent with the experimentally observed partial transformation of Ag_(1)_ into Ag_(2)_ over time, while Ag_(1)_ is kinetically preferred during intercalation due to its closer lattice matching with the SiC substrate. This competition enables tunable phase coverage through defect engineering. Angle‐resolved photoemission spectroscopy (ARPES) shows that both Ag phases form semiconducting 2D layers and induce n‐type doping of the graphene overlayer, with stronger charge transfer observed for Ag_(2)_ owing to its higher Ag areal density. Spectroscopic imaging ellipsometry (SIE) captures phase‐dependent differences in optical absorption, while second‐harmonic generation (SHG) measurements reveal a three‐order‐of‐magnitude difference in second‐order susceptibility between the two structures. Together, these results demonstrate that graphene defects are not merely gateways for intercalation, but deliberate design elements that couple phase formation with electronic and optical functionality in atomically thin metals.

### 1.1 Intercalation of Two‐Dimensional Silver Under Distinct Graphene Templates

Two‐dimensional (2D) Ag is synthesized via the CHet method, following the procedure described by Briggs et al. In this process, epitaxial graphene (EG) is first prepared on 6H‐SiC substrates via high‐temperature silicon sublimation, initially forming zero‐layer graphene (ZLG), followed by monolayer EG [52, 53]. The EG films are then treated with oxygen or helium plasma to introduce defects [40, 54], which facilitates the intercalation of Ag atoms at the EG/SiC interface during thermal evaporation at 900°C for 1 h [22]. As illustrated schematically in Figure 1a, all three forms of graphene—ZLG, pristine EG, and plasma‐treated EG—enable Ag intercalation, leading to the formation of large‐area, crystalline 2D Ag films. These graphene substrates differ in carbon layer structure and thickness as well as by the defect types within the layer. During intercalation, the defects in ZLG and EG tend to self‐heal [40], and ZLG transforms into a free‐standing graphene layer after silver intercalation [22, 33], yielding either monolayer or bilayer graphene capping the 2D Ag. The intercalated 2D Ag can form two distinct structural phases, either the Ag_(1)_ phase in (1 × 1) registry with SiC, and the denser Ag_(2)_ phase [51].

**FIGURE 1:**
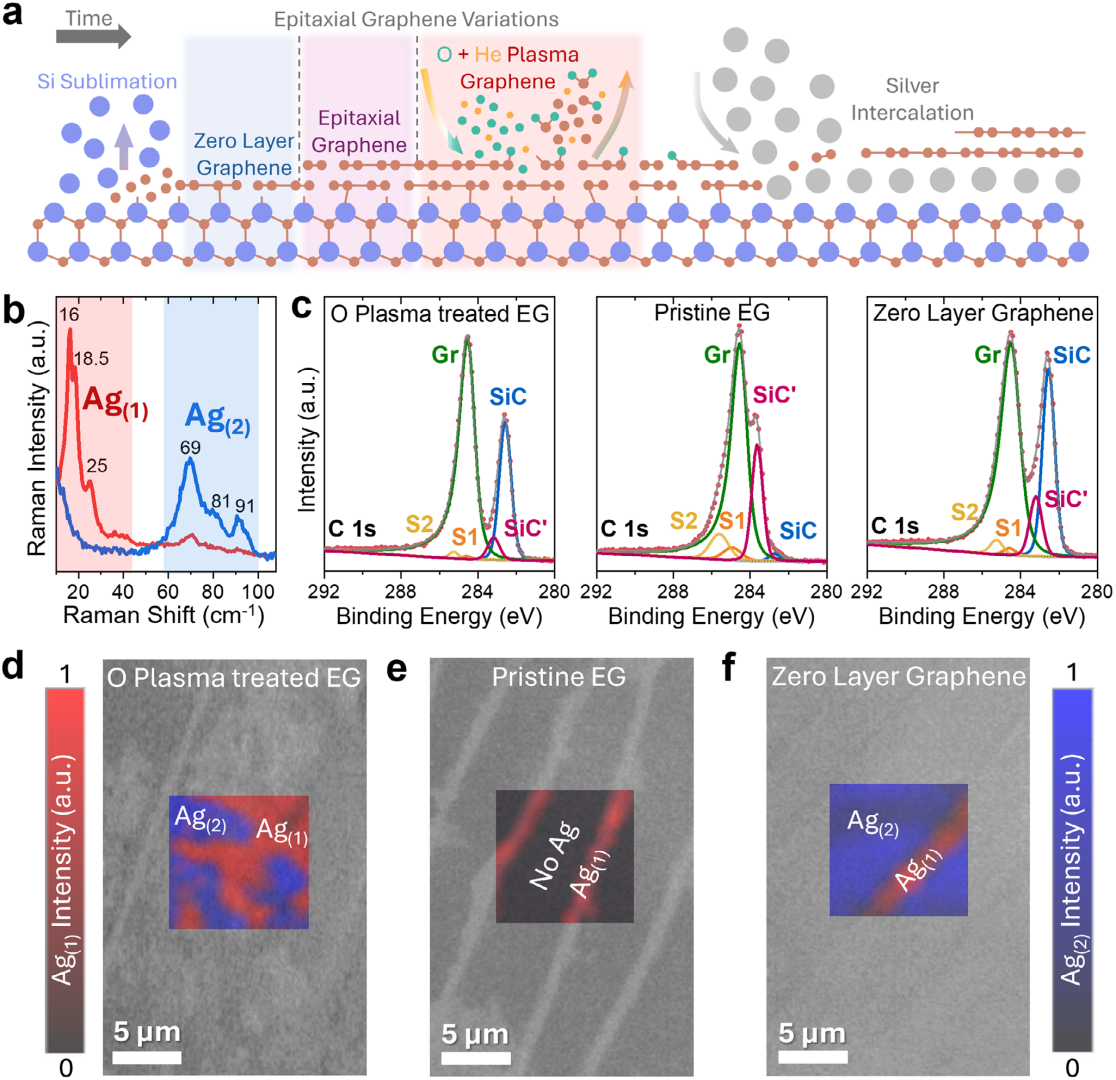
Defect‐templated confinement heteroepitaxy enables phase‐selective synthesis of two‐dimensional silver. (a) Schematic illustration of silver intercalation at the graphene/SiC interface using zero‐layer graphene (ZLG), pristine epitaxial graphene (EG), and oxygen plasma‐treated EG, highlighting how distinct defect motifs and thickness in the graphene overlayer mediate access to the buried interface. (b) Characteristic ultralow frequency (ULF) Raman spectra identifying the Ag_(1)_ and Ag_(2)_ phases. (c) X‐ray photoelectron spectroscopy (XPS) of the C 1s spectra for Ag‐intercalated O plasma treated EG, pristine EG and ZLG, indicating varying degrees of buffer layer decoupling as evidenced by the Gr‐SiC peak splitting. (d–f) Grayscale optical images of Ag intercalated beneath (d) O plasma‐treated EG, (e) pristine EG, and (f) ZLG. The insets show 10 × 10 µm^2^ ULF Raman maps, with mapped Raman intensity corresponding to Ag_(1)_ Raman modes (16, 18.5, and 25 cm^−1^) shown in red, and Ag_(2)_ Raman modes (69, 81, and 91 cm^−1^) shown in blue, corresponding to the spectra in panel (b). Color bars represent the relative Raman intensities for each phase (Ag_(1)_—red and Ag_(2)_—blue). This figure introduces defect chemistry in the graphene overlayer as a deterministic design parameter for phase selection in confined two‐dimensional silver.

The intercalation of Ag is effectively identified by the emergence of distinct ultralow frequency (ULF) Raman modes unique to each phase (Figure 1b) [50]. The Ag_(1)_ phase exhibits sharp Raman peaks near 16, 18, and 25 cm^−1^, whereas the Ag_(2)_ phase displays three prominent features at 69, 81, and 91 cm^−1^. These ULF Raman modes arise from shear and out‐of‐plane vibrations involving interactions between the intercalated silver atoms and the underlying silicon bonds [55], similar to observations in other 2D metals such as Ga and In [34]. Additionally, x‐ray photoelectron spectroscopy (XPS) characterization of the carbon 1s spectra (Figure 1c) confirms the decoupling of the ZLG layer by the intercalant, as indicated by the characteristic splitting between the sp^2^ carbon (284.5 eV) typical for graphene (Gr), the SiC substrate peaks (282.6 eV) position due to band bending and the reduction in the S1 (284.6 eV) and S2 (285.2 eV) buffer layer peaks [21]. A separation greater than 1.9 eV was observed for intercalated 2D silver on both ZLG and oxygen plasma treated EG, consistent with effective decoupling of the ZLG from the SiC to quasi‐freestanding monolayer graphene (QFMLG) and complete Ag intercalation. In contrast, pristine EG samples exhibited a smaller C 1s separation of 1.76 eV, with remnant buffer peaks (S1, S2) and a pronounced SiC’ peak from the unintercalated regions of SiC without band bending at 283.6 eV, signifying incomplete intercalation and highlighting the critical role of defects in facilitating Ag incorporation [40, 56, 57]. The evolution of the C 1s and Si 2p spectra for these samples is shown in Figure S1. The Ag 3d XPS and Ag MNN Auger spectra (Figure S2) further confirm the presence of intercalated 2D Ag in the ZLG and oxygen plasma‐treated EG samples [58].

Ag_(1)_ and Ag_(2)_ exhibit markedly different optical absorption behaviors, as measured via differential reflectance spectroscopy. The Ag_(1)_ phase shows two distinct absorption maxima at approximately 470 and 640 nm, whereas the Ag_(2)_ phase features a broad absorption peak centered near 570 nm (Figure S3). These features are associated with localized‐plasmon‐like interband transitions, characteristic of intercalated 2D metal systems [58]. The resulting contrast in the dielectric functions associated with the absorption behavior enables rapid, phase‐sensitive identification under an optical microscope (Figure 1d–f). Using the standard CHet intercalation method on oxygen plasma‐treated EG substrates, we achieve complete silver coverage across the 5 × 10 mm^2^ samples, with both Ag_(1)_ and Ag_(2)_ phases present. Phase distribution is confirmed by Raman mapping of their respective ULF signatures (Figure 1d), revealing approximately equal proportions of each phase. Correlative Raman and optical imaging shows that Ag_(2)_ regions have a consistently stronger reflective component in the visible range than Ag_(1)_ due to their differing absorption (Figure S3). When the intercalation duration is reduced from 60 to 40 min, we observe preferential formation of Ag_(1)_ along terrace edges, with incomplete intercalation and small, mixed‐phase domains across the terrace surface (Figure S3). These observations suggest that Ag_(1)_ is the kinetically favored phase, nucleating preferentially at terrace edges in oxygen plasma‐treated EG substrates.

Defect density in the graphene overlayer plays a critical role in initiating and guiding silver intercalation at the EG/SiC interface. To elucidate this role, we performed silver intercalation on a pristine EG sample without any plasma treatment, which is expected to exhibit a very low density of defects. Optical imaging and Raman mapping of the ULF region reveal only limited intercalation (<10%), with minimal to no 2D Ag coverage observed on the terraces (Figure 1e). Even in the absence of intentional defect introduction, localized intercalation of the Ag_(1)_ phase is observed at terrace edges, where thicker graphene layers typically reside [53, 59]. This behavior is consistent with the well‐established understanding that epitaxial graphene nucleates at SiC terrace edges, which are inherently rich in structural defects [59]. These results further support the speculation that Ag_(1)_ is the kinetically favored phase that forms first, and that specific defect types, such as those naturally occurring at SiC step edges, play a pivotal role in directing nucleation.

Intrinsic sp^3^‐type defects in ZLG enable preferential formation of the Ag_(2)_ phase during intercalation. Although ZLG exhibits a graphene‐like structure, approximately one‐third of its carbon atoms are covalently bonded to the underlying SiC substrate through sp^3^ hybridization [60, 61, 62]. This bonding configuration introduces a high density of intrinsic sp^3^‐type defects [63], which are known to facilitate intercalation without the need for ex situ defect generation [21, 64]. As shown in Figure 1e, optical imaging and ULF Raman mapping reveal extensive intercalation of the Ag_(2)_ phase across ZLG terraces, with localized Ag_(1)_ formation at terrace edges, underneath multilayer graphene. The dominant presence of Ag_(2)_ on ZLG suggests that specific defect types may preferentially promote the nucleation and stabilization of this higher‐order phase. Notably, utilizing molecular beam epitaxy (MBE)—an ultrahigh vacuum (UHV) technique—in combination with ZLG grown in Argon atmosphere using and RF (radio frequency) furnace leads only to the formation of Ag_(1)_ [33]. In contrast, the CHet‐based intercalation consistently yields Ag_(2)_ (Figure S4), indicating that differences in intercalation procedures (near atmosphere [22, 40] vs. MBE [33]), ZLG defect density (ZLG grown with RF or ordered ZLG, Figure S4a) and exposure to the ambient environment impact the defect density and chemistry (e.g., adsorption of molecules), and thus strongly influence phase formation.

### 1.2 Phase Engineering of Two‐dimensional Silver Through Graphene Defect Chemistry

Building on the phase selectivity observed for intrinsic sp^3^‐type defects in ZLG, we next investigate whether intentional defect engineering of epitaxial graphene (EG) can be used to deterministically program the structural phase of two‐dimensional Ag. Tailoring defect types in EG through controlled plasma treatment enables systematic modulation of intercalation pathways and phase outcome. To achieve uniform stabilization of the Ag_(1)_ phase, growth conditions were optimized by selectively modifying the defect landscape of EG using remote plasma treatments with different gas compositions (O_2_ and He) and exposure durations. Tailoring defect types in epitaxial graphene through plasma treatment enables controlled phase selection of 2D Ag. To achieve uniform growth of Ag_(1)_, growth conditions were optimized by systematically modifying the remote plasma treatment of epitaxial graphene (EG) using different gas compositions (O_2_ and He) and exposure durations. Plasma treatments are known to generate specific defect types in graphene [54], which can be identified by the D/D′ intensity ratio in Raman spectroscopy [65]. Literature reports associate D/D′ values of ∼ 13, ∼ 7, and ∼ 3 with sp^3^‐type, vacancy‐type, and line (boundary) defects, respectively, depending on the plasma chemistry used [65, 66, 67, 68, 69]. Raman mapping of treated EG samples revealed distinct trends in D/D′ ratios as a function of plasma parameters (Figure 2a). A 10‐s O/He plasma exposure produced a D/D′ ratio of ∼ 9.2, indicative of mixed sp^3^ and vacancy‐type defects. Switching to pure He plasma reduced the ratio to ∼ 7.6, entering the vacancy‐type defect regime. Increasing He plasma duration to 2 and 3 min further lowered the D/D′ ratios to ∼ 4.6 and ∼ 2.1, respectively, marking a transition toward boundary‐defect‐type dominance. Corresponding average Raman spectra of plasma‐treated EG (Figure 2b), show broadening of the D peak and the emergence of a pronounced D′ shoulder at longer exposures, consistent with increased structural disorder and the formation of extended line defects [67]. In contrast, pristine EG showed minimal D and no visible D′ peak, while zero‐layer graphene (ZLG) exhibited a Raman profile characteristic of a well‐ordered buffer layer [62, 70, 71].

**FIGURE 2:**
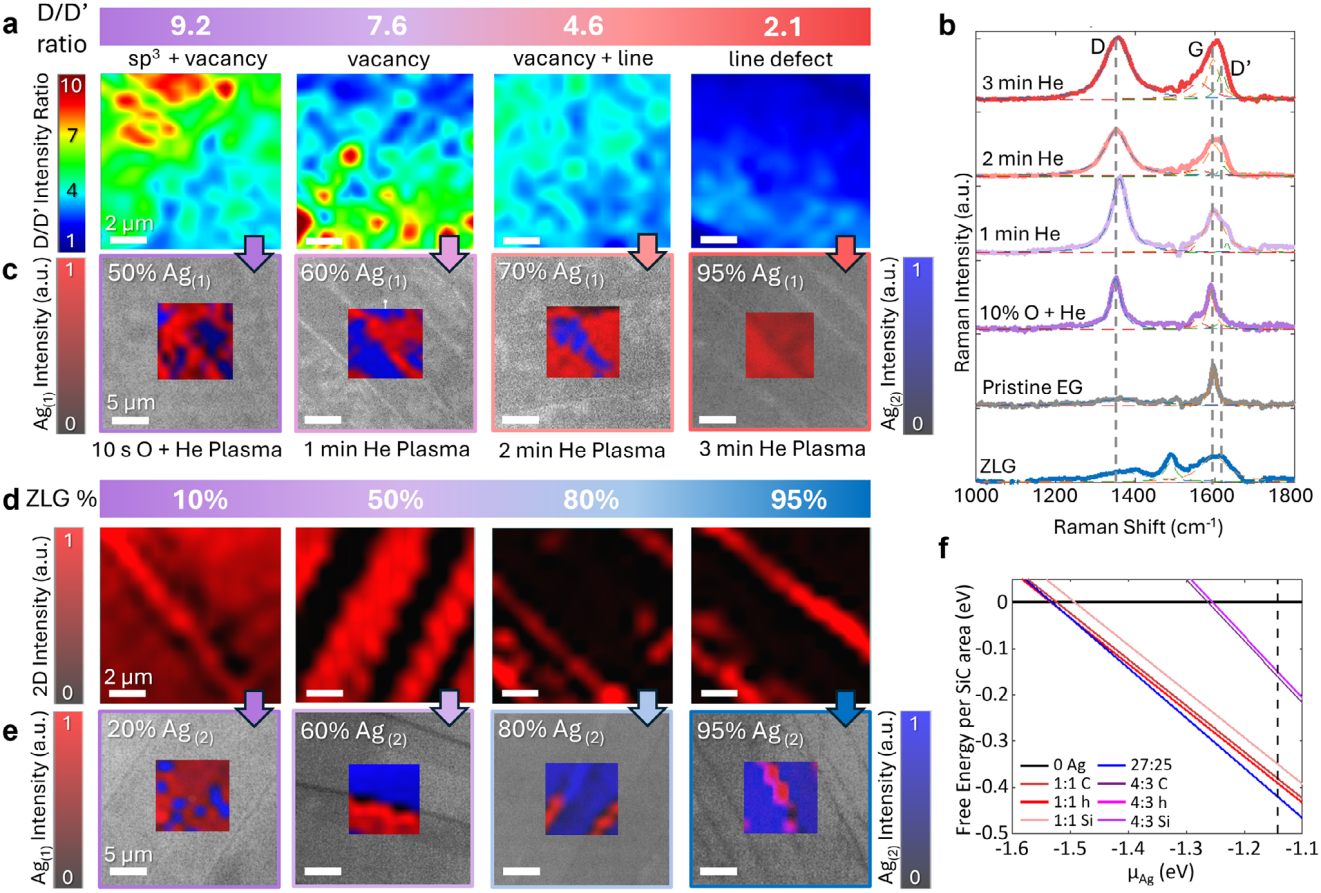
Engineering structural phase formation in two‐dimensional silver through graphene defect chemistry. (a) Raman maps of the D/D′ intensity ratio for epitaxial graphene (EG) substrates treated with 10 s oxygen/helium plasma, and with 1, 2, and 3 min helium plasma, illustrating systematic evolution of defect type from sp^3^‐ and vacancy‐dominated motifs toward extended line defects (scale bars: 2 µm). (b) Representative background subtracted Raman spectra of EG substrates (pristine, zero‐layer graphene (ZLG), and various plasma treatments), showing fitted D, G, and D′ peaks to assess defect nature and density. (c) Grayscale optical images of Ag‐intercalated samples prepared on the EG substrates shown in (a), with insets showing corresponding ultralow frequency (ULF) Raman maps identifying Ag_(1)_ (red) and Ag_(2)_ (blue) phases (scale bars: 5 µm). (d) Raman 2D peak maps of EG samples with increasing ZLG surface coverage (10%, 50%, 80%, and 95%), highlighting monolayer EG regions (red) and exposed ZLG terraces (black) (scale bars: 2 µm). (e) Grayscale optical images of Ag‐intercalated samples with 20%–95% ZLG coverage. Insets show ULF Raman maps identifying Ag_(1)_ (red) and Ag_(2)_ (blue) phase distributions. Scale bars: 5 µm. (f) Calculated phase diagram for Ag intercalation on SiC (without graphene), showing formation energy per SiC surface unit cell as a function of Ag chemical potential. “0Ag” refers to the nonintercalated system. The dashed line indicates the chemical potential of bulk Ag. The lowest‐energy configuration at each chemical potential identifies the thermodynamically preferred phase, revealing a competition between the 1:1 (Ag_(1)_) and 27:25 (Ag_(2)_) phases. C, Si, and h labels denote Ag atoms on top of carbon, silicon, and hollow sites, respectively (Figure S8). *Note*: 4:3 Si has a slightly lower free energy compared to 4:3 h site registry. Together, these data link graphene defect motifs to phase outcome and provide a mechanistic framework for defect‐templated phase programming in confined two‐dimensional metals.

Defect engineering via helium plasma treatment enables controlled, phase‐pure growth of 2D Ag_(1)_. Silver intercalation at 900°C for 1 h on plasma‐treated EG substrates from Figure 2a resulted in distinct phase outcomes depending on the defect profile. The standard CHet process using O/He plasma treatment [22, 40] produced complete intercalation with approximately equal fractions of Ag_(1)_ and Ag_(2)_ phases (Figure 2c). Helium plasma treatment for 1‐min increased Ag_(1)_ domain size and coverage to ∼ 60%, suggesting that vacancy‐type defects favor Ag_(1)_ nucleation. Extending plasma exposure to 2 min further boosted Ag_(1)_ coverage to ∼ 70%, reinforcing the role of defect type and density. At 3 min of helium plasma exposure, corresponding to the line defect regime, Ag_(1)_ coverage exceeded 95%, yielding large‐area, phase‐pure 2D Ag_(1)_.

XPS analysis of this 3‐mi helium‐treated sample before and after intercalation (Figure S5) showed a C 1s peak splitting >1.9 eV and the emergence of Ag^0^ and Ag‐Si 3d peaks, confirming both successful intercalation and graphene‐SiC decoupling. These observations suggest that larger, plasma‐induced defects—particularly line defects—facilitate direct access for Ag atoms to the underlying SiC, enabling preferential nucleation of the (1 × 1) Ag_(1)_ phase over the denser Ag_(2)_ structure. This mechanism aligns with earlier observations of Ag_(1)_ formation along terrace edges of pristine EG (Figure 1e), which are naturally enriched with such line defects. Larger defects likely provide pathways for direct Ag–SiC interaction, promoting the formation of the commensurate Ag_(1)_ lattice [72]. Although the as‐synthesized Ag_(1)_ film appears uniform and phase‐pure, it remains metastable and undergoes a partial transformation (∼30% conversion) into the Ag_(2)_ phase over a period of 10 weeks, akin to Ostwald ripening, even when stored in a nitrogen‐filled environment at ambient temperature (Figure S6). This long‐term phase evolution highlights the thermodynamic preference for Ag_(2)_ and sets the stage for a deeper discussion of the relative stability between the two phases in the following sections.

To promote large‐area Ag_(2)_ phase growth, sp^3^‐type defects were targeted as effective intercalation sites by leveraging ZLG substrates. Unlike EG, which requires plasma treatments to introduce sp^3^ defects, ZLG naturally contains a high density of such defects due to partial covalent bonding between carbon atoms and the underlying SiC substrate [75]. Prior studies have shown that short‐duration oxygen plasma treatments can introduce sp^3^‐type defects into graphene [67], but these often result in insufficient defect densities or transition toward vacancy‐type defects at longer exposures. To avoid these limitations, undergrown EG samples with varying degrees of ZLG contribution were synthesized and characterized using 2D Raman peak mapping (Figure 2d). Spatially resolved Raman intensity maps revealed regions with monolayer EG coverage (bright red) and ZLG terraces (dark), corresponding to the presence or absence of the prominent 2D Raman peak. Atomic force microscopy (AFM) adhesion imaging (Figure S7) further confirmed the spatial variation, with ZLG regions exhibiting distinct adhesion contrast.

Modulating the spatial coverage of exposed ZLG and EG enables defect‐engineered control over Ag phase intercalation and distribution. Pristine EG substrates with <5% exposed ZLG (>95% EG/ZLG) coverage exhibit minimal Ag intercalation due to a lack of accessible interfacial pathways (Figure 1e). Introducing ∼ 10% ZLG dramatically enhances intercalation and yields predominantly Ag_(1)_, suggesting that sp^3^‐defect‐rich ZLG regions trap Ag atoms and facilitate lateral diffusion beneath adjacent EG, enabling widespread metal access. Increasing ZLG coverage to 50% produces coexisting Ag_(1)_ and Ag_(2)_ phases, with Ag_(2)_ localized beneath ZLG and Ag_(1)_ beneath EG, indicating that intercalation pathways extend across phase boundaries while preserving local phase selectivity. Further increasing ZLG coverage to 80% and 95% promotes dominant Ag_(2)_ formation across terraces, with residual Ag_(1)_ appearing along terrace edges and beneath EG regions—echoing trends observed in pristine and plasma‐treated EG substrates. These results support the hypothesis that ZLG's small, localized sp^3^‐type defects favor nucleation of the higher‐order Ag_(2)_ phase, while larger, extended defects promote Ag_(1)_. Additionally, enhanced charge transfer at the ZLG/SiC interface [73] may further stabilize Ag_(2)_ by modifying interfacial electronic structure. Together, these templating and electronic effects drive rapid growth and stabilization of large‐area, phase‐pure Ag_(2)_ films on ZLG. However, MBE‐based Ag intercalation on RF‐grown ZLG still leads to the Ag_(1)_ phase [33] as the RF‐grown ZLG is less defective in comparison to ZLG grown by resistive heating.

First‐principles calculations evaluating the relative phase stability, finds that Ag_(2)_ is more likely to be thermodynamically favored over Ag_(1)_. The intercalation formation energy density (per SiC surface unit) was computed using:

(1) $$\frac{F}{A}=\frac{E-\left(E_{0Ag}+n_{Ag}\mu_{Ag}\right)}{n_{SiC}}$$

where *E* is the energy of the intercalated system, *E*_0Ag_ is the energy of the nonintercalated system, *n*_Ag_ is the number of Ag atoms, µ_Ag_ is the chemical potential of Ag, and *n*_SiC_ is the number of SiC primitive cells on the surface in the computational unit cell. These calculations, performed without the graphene overlayer, are summarized in Figure 2f. For each value of silver chemical potential, the phase with the lowest formation energy density is thermodynamically the most stable. Inside the chemical potential energy window where the intercalation happens, the Ag_(2)_ phase (*n*_Ag_: *n*_SiC_ = 27: 25) is found to be the most stable over a broad range, consistent with the experimentally seen transition from Ag_(1)_ to Ag_(2)_ over time in Figure S6. Additionally, the formation energy density of the Ag_(1)_ phase (*n*_Ag_: *n*_SiC_ = 1: 1) of carbon (C) and hollow‐site (h) registries (Figure S8) is very close to that of the Ag_(2)_ phase, which offers the opportunity to tune the relative areal coverage of each phase through graphene defect engineering. Additionally, the relatively small formation energy difference between Ag_(1)_ and Ag_(2)_ suggests that modest modifications to the local energetic landscape, such as changes to the interfacial bonding environment or the introduction of additional kinetic barriers, may provide viable routes toward extending the lifetime of the metastable Ag_(1)_ phase. In contrast, the 4:3 phase ( *n*_Ag_: *n*_SiC_ = 4: 3), which has been proposed in high‐throughput computational [74] and experimental [51] studies, exhibits significantly higher formation energy density and is therefore unlikely to form under experimental conditions. Altough this thermodynamic analysis excludes the effects of graphene or entropy, it offers a semiquantitative framework that aligns well with the observed phase evolution trends.

The thermodynamic stability of intercalated 2D Ag phases arises from a balance between Ag─Ag cohesion and Ag–SiC substrate interactions, which can be interpreted by comparing Ag─Ag bond lengths across different packing configurations (Figure S9). Bulk FCC Ag exhibits a bond length of 2.88 Å in its (111) plane. In the commensurate (1:1) Ag_(1)_ phase, the Ag─Ag bond length increases to 3.1 Å, matching the Si─Si spacing in the SiC substrate and indicating stronger Ag─SiC bonding that stabilizes this phase despite weaker Ag─Ag interactions. In contrast, the (27:25) Ag_(2)_ phase shows a shorter Ag─Ag bond length of 2.98 Å, closer to the bulk value, suggesting that enhanced Ag─Ag interactions drive the formation of this higher‐order phase. The previously proposed 4:3 phase [51, 74], while offering better geometric commensurability than the (27:25) phase, exhibits a significantly short Ag─Ag bond length of 2.68 Å, making it energetically unfavorable. Together, these results indicate that Ag_(1)_ and Ag_(2)_ represent the only low‐energy configurations accessible under the present growth conditions, while alternative packing motifs are destabilized by unfavorable strain and Ag─Ag bond compression.

In addition to these thermodynamic trends, kinetic and structural considerations play a critical role in determining phase formation. As shown in Figure S10, Ag clusters with diameters around 12 Å fail to stabilize the Ag_(2)_ configuration. The high edge‐to‐area ratio in such small clusters diminishes the stabilizing Ag─Ag interactions needed for Ag_(2)_, making it less favorable during the early nucleation stage. The Ag_(1)_ phase, by contrast, forms more readily due to stronger Ag–SiC interfacial interactions. Thus, under growth conditions involving rapid intercalation—such as regions with line defects (Figure 2c) or terrace edges (Figure 1e)—the system preferentially forms Ag_(1)_. These observations highlight a defect‐ and kinetics‐mediated pathway for phase selection in 2D confined metals.

### 1.3 Phase‐Dependent Structural and Electronic Properties of 2D Ag

Structural characterization confirms the distinct lattice symmetries and superstructures of the Ag_(1)_ and Ag_(2)_ phases. Figure 3a shows low‐energy electron diffraction (LEED) patterns for the two phases, Ag_(1)_ in the upper half, Ag_(2)_ in the lower half, both with the typical first order diffraction, i.e., (10) spots originating from the primitive cells of graphene and SiC (indicated by white and orange circles, respectively). The Ag_(2)_ phase in the lower half exhibits a distinct LEED pattern (Figure 3a (lower half)) with a number of additional spots. Most prominently, six satellite spots arise around the graphene (10) spots (blue circles) corresponding to an approximate (6.25 × 6.25) grid with respect to graphene. We attribute these to a buckling or a double diffraction superstructure of the graphene on top of the Ag_(2)_ structure in close correspondence with the STM findings (see below). In the Ag_(1)_ pattern (upper half), this spot group is also visible but much weaker, owing to the low percentage residual Ag_(2)_ contribution in this sample. Otherwise, the Ag_(1)_ pattern contains no additional diffraction features, since the Ag_(1)_ phase should be nearly commensurate with the substrate (Ag:SiC ≈ 1:1) [21, 33, 49]. Notably, in the Ag_(2)_ pattern also faint superstructure spots of the (6√3 × 6√3)R30° supercell of epitaxial orientation of graphene on SiC (green circles) are visible, that arise from the presence of residual non‐intercalated areas (see also XPS in Figure 1c). They are absent in the Ag_(1)_ pattern, either due to more complete intercalation or the damping through the bilayer EG cover. Last but not least, we observe five spots in the central region between the electron gun and the graphene (10) spot three of which are part of the (6√3 × 6√3)R30° superstructure (green circles), and two of which correspond to a (5 × 5) mesh with respect to SiC [21, 33, 49]. All these superstructures align with the geometry of the Ag_(2)_ phase (Table S1, Figure S11), which consists of a (3√3 × 3√3)R30° Ag lattice commensurate with a (5 × 5) SiC unit cell, also referred to as the “27:25” structure due to its Ag:SiC atomic ratio. The 6.1 × 6.1 graphene supercell originates from commensuration between the Ag and graphene layers [75], while the (6√3 × 6√3) SiC superstructure results from the lattice match between graphene and SiC. Schematic atomic structures of both phases are illustrated in Figure 3b: the Ag_(1)_ phase forms a (1 × 1) triangular lattice aligned with the SiC substrate, analogous to the 1:1 structures observed in CHet‐grown Ga and In [40], while the Ag_(2)_ phase adopts a higher‐order (3√3 × 3√3)R30° configuration over a (5 × 5) SiC supercell.

**FIGURE 3:**
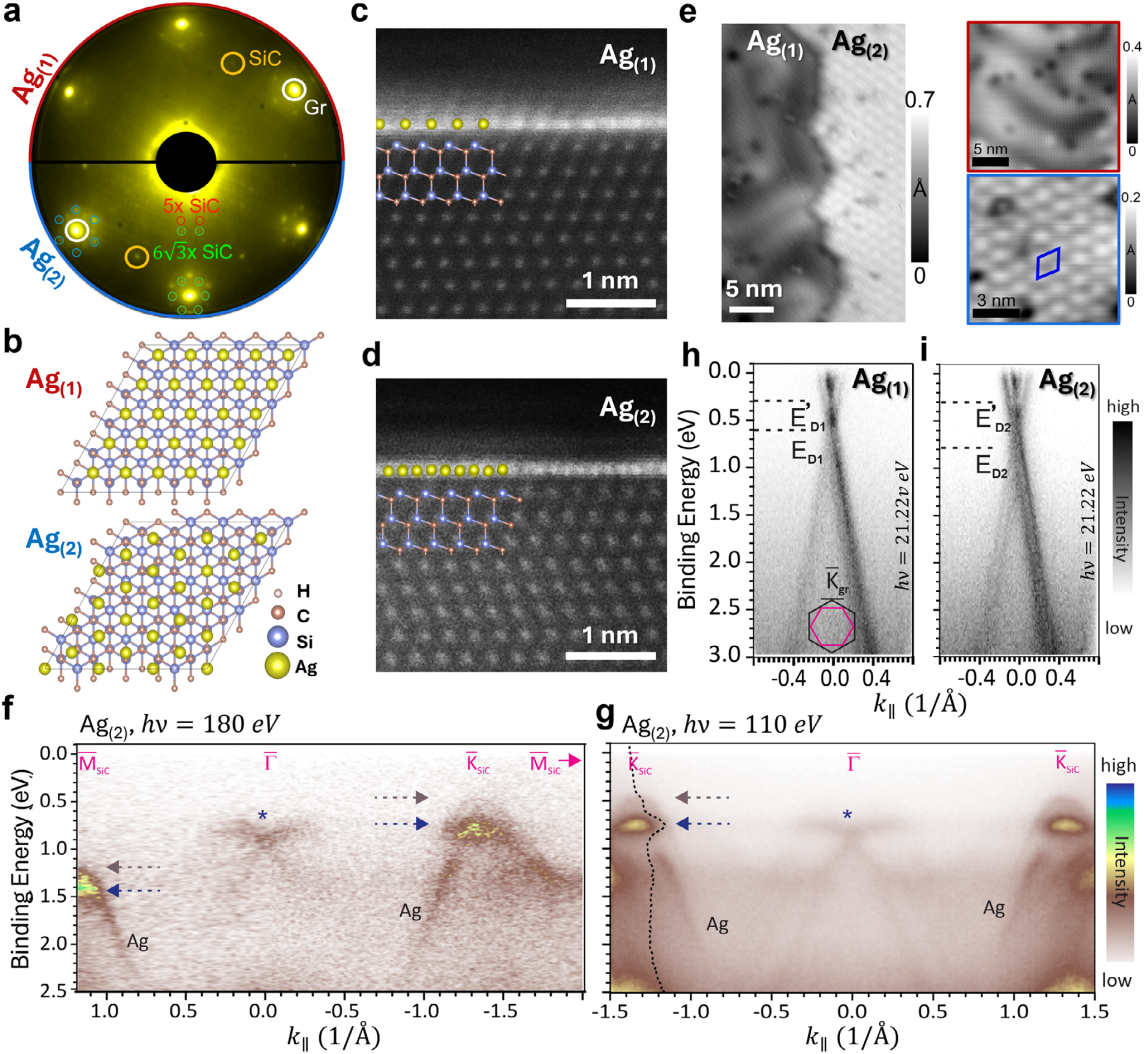
Phase‐dependent structure‐property relationships in 2D Ag. (a) Low‐energy electron diffraction (LEED) patterns of the Ag_(1)_ and Ag_(2)_, revealing distinct lattice registry with the SiC substrate. (b) Atomic models of the Ag_(1)_ and Ag_(2)_, illustrating their respective 1:1 and 27:25 commensuration with the SiC substrate (graphene layer omitted for clarity). (c, d) Cross‐sectional transmission electron microscopy (TEM) images of (c) Ag_(1)_ and (d) Ag_(2)_, with corresponding structural models overlaid, confirming monolayer thickness and atomic registry. (e) Large‐area scanning tunneling microscopy (STM) image acquired at 2 V, showing a boundary between Ag_(1)_ and Ag_(2)_; high‐resolution STM images of each phase are outlined in red (Ag_(1)_) and blue (Ag_(2)_). The superstructure periodicity in Ag_(2)_, shown by the blue rhombus, is ∼ 14.7 Å. (f, g) High‐resolution angle‐resolved photoemission spectroscopy (ARPES) maps for Ag_(2)_– quasi‐free‐standing monolayer graphene (QFMLG) showing Ag_(2)_‐band dispersion along (f) $\bar{M_{\mathrm{SiC}}\Gamma K_{\mathrm{SiC}}}$ (180 eV photons) and (g) $\bar{K_{\mathrm{SiC}}\Gamma K_{\mathrm{SiC}}}$ (110 eV photons), with the energy distribution curve at $\bar{K_{\mathrm{Ag}}}$ overlaid in (g). Brown and blue arrows in (f, g) mark characteristic bands of Ag_(1)_ and Ag_(2)_, respectively; blue asterisks in (g, h) denote Ag bands backfolded via Umklapp scattering from the graphene Brillouin zone. (h, i) Graphene π‐band dispersion in (h) Ag_(1)_ quasi‐free‐standing bilayer graphene (QFBLG) and **(i)** Ag_(2)_ QFMLG, recorded at $\bar{K_{\mathrm{gr}}}$ perpendicular to $\bar{\Gamma K_{\mathrm{gr}}}$ direction, measured by ARPES using lab‐based He Iα radiation. Dirac points are labeled E’_D1(2)_ and E_D1(2)_ for Ag_(1)_ and Ag_(2)_, respectively. These measurements demonstrate that defect‐engineered Ag phases exhibit distinct atomic registry and electronic coupling to the graphene overlayer.

Cross‐sectional transmission electron microscopy (TEM) confirms the monolayer nature and atomic registries of both phases. In the Ag_(1)_ phase, the silver atoms exhibit a nearly 1:1 registry with the underlying Si atoms (Figure 3c), while the Ag_(2)_ phase is more densely packed, matching the 27:25 atomic configuration (Figure 3d). The overlayed corresponding structural models align well with the observed atomic positions. Large‐area TEM imaging and energy‐dispersive x‐ray spectroscopy (EDS) maps (Figure S12), further confirm the presence of continuous, monolayer 2D Ag films. Interlayer spacing between graphene and the intercalated silver reveal distinct differences between the two phases: 0.32 nm for Ag_(2)_ and 0.47 nm for Ag_(1)_ (Figure S12). This substantial difference suggests a stronger interaction between graphene and the Ag_(2)_ phase, tentatively due to larger charge transfer from Ag_(2)_ to the graphene (Figure 3f, Figure S13), indicating that the graphene overlayer may play an active role in stabilizing specific structural phases during or after intercalation.

Correlative multimodal characterization using Raman, SEM, AFM, and AES confirms structural and electronic distinctions between Ag_(1)_ and Ag_(2)_. These measurements validate differences in packing density, strain/doping in overlying graphene, and silver–graphene interaction strength. A detailed analysis is provided in the Supporting Information (Note, Figures S14–S15).

Scanning tunneling microscopy (STM) reveals atomic‐scale structural and electronic properties of intercalated 2D Ag. Large‐area STM topography acquired at +2 V (bias at sample, Figure 3e) clearly resolves adjacent domains of the Ag_(1)_ and Ag_(2)_ phases, separated by a well‐defined boundary. Epitaxial graphene is observed atop both phases but becomes visible only at low bias voltages near the Fermi level due to its weak coupling with the underlying Ag (Figure S16). The Ag_(1)_ phase exhibits a nonperiodic structure with elongated bright and dark stripes, resembling a herringbone‐like pattern in Au(111) [76]. We tentatively attribute this modulation to in‐plane stress variations within the Ag_(1)_ layer, possibly arising from Ag atoms alternating between hollow and carbon‐site positions beneath graphene, driven by small energy differences between these configurations (Figure S8) [77].

In contrast, the Ag_(2)_ phase displays a bright‐dot superstructure with a periodicity of ∼14.7 Å (highlighted by a blue rhombus), which is commensurate to six times the graphene unit cell (14.76 Å). This periodic modulation is attributed to a moiré pattern resulting from lattice mismatch and angular misalignment between the 27:25 Ag_(2)_ superstructure and the graphene overlayer. The resulting moiré leads to long‐range electronic potential modulation at the graphene/Ag interface. Notably, the Ag_(2)_ domains appear slightly elevated relative to Ag_(1)_, though this apparent height difference is bias‐dependent. Point defects are also observed in both phases, likely originating from Ag vacancies in the intercalated layer or Si adatoms exchanged with the uppermost SiC substrate [78]. Differential conductance (dI/dV) spectroscopy reveals distinct electronic features of the intercalated 2D Ag phases. Both Ag_(1)_ and Ag_(2)_ exhibit a steep increase in conductance at large positive bias (Figure S16), diverging from the density of states expected for pristine graphene [79], with Ag_(2)_ showing conduction band onset at ∼ 0.2 eV higher energy than Ag_(1)_. This behavior likely originates from contributions of Ag d_x_‐type orbitals [74].

In angle‐resolved photoemission spectroscopy (ARPES) measurements, the presence of sharp Ag_(1)_ (Figure S17a) and Ag_(2)_ (Figure S17b) bands confirms their well‐ordered nature after intercalation. The Ag_(1)_ band (Figure S17a), closely matching previously reported data [33, 49], indicates the semiconducting character. The Ag_(2)_ band structure, recorded along the $\bar{M_{SiC}\Gamma K_{SiC}}\;$ direction using 180 eV photons, is shown in Figure 3f. The absence of any band crossing the Fermi level further confirms the semiconducting nature of Ag_(2)_. Band splitting in Ag_(2)_ (highlighted by blue and brown arrows) is evident at both the $\bar{M_{SiC}}$ and $\bar{K_{SiC}}$​ points. To characterize this splitting at ${\bar{K}}_{SiC}$, ARPES data were collected along the $\bar{M_{SiC}\Gamma K_{SiC}}\;$ direction using an excitation energy of 110 eV (Figure 3g), which enhances the Ag photoemission cross‐section. The energy distribution curve (EDC) (black dashed line in Figure 3g) at ${\bar{K}}_{SiC}$​ shows two peaks at 0.50 eV (brown arrow) and 0.75 eV (blue arrow) binding energy (BE). The weaker peak at 0.50 eV aligns well with the Ag_(1)_ band maximum [33, 80], while the 0.75 eV peak corresponds to the Ag_(2)_ valence band maximum (VBM). This reveals an energy offset of approximately 250 meV between the Ag_(2)_ and Ag_(1)_ VBMs. The faint bands marked by blue asterisks in Figure 3f,g near the Γ point correspond to Umklapp‐scattered Ag valence bands arising from the graphene Brillouin zone [8, 33].

Figure 3h,i presents ARPES measurements (lab‐based He Iα) of the Dirac cones originating from the decoupled graphene layer, recorded at the graphene K point (${\bar{K}}_{gr}$) after Ag_(1)_ and Ag_(2)_ intercalation, respectively. Here, *K*_||_ is oriented perpendicular to the $\;\bar{\Gamma K_{gr}}\;$​direction, as indicated in the inset of Figure 3h. In both datasets, two distinct sets of Dirac cones are observed. For the Ag_(1)_‐intercalated sample, (Figure 3h), the Dirac points (DPs) appear at approximately 0.3 eV BE (*E*′_*D*1_) and 0.6 eV BE (*E*_*D*1_). In the Ag_(2)_ sample (Figure 3i), the corresponding Dirac points are labeled as *E*′_*D*2_ and *E*_*D*2_, respectively. The presence of these dual Dirac cones is more clearly resolved in the synchrotron‐based high‐resolution data for the Ag_(2)_‐intercalated sample (Figure S17d). The lower‐binding‐energy Dirac point *E*′_*D*2_ (weaker band) corresponds to bilayer graphene (BLG) bands, as confirmed by the observable splitting in the lower part of that cone [81], a feature not resolved in the lab‐based ARPES data due to the energy resolution limit. The higher‐binding‐energy Dirac point *E*_*D*2_​ is associated with the quasi‐free monolayer graphene (MLG) Dirac cone, while the intensity elongation between 0.8 and 1.2 eV arises from electron–plasmon coupling [33, 80, 82, 83]. In the Ag_(1)_ sample, the BLG Dirac cone (*E*′_*D*1_) exhibits stronger intensity. Since this sample was prepared from monolayer graphene on SiC, Ag_(1)_ intercalation likely leads to decoupling of a BLG layer formed during intercalation, whereas the weaker MLG feature (*E*_*D*1_) results from decoupling of residual buffer‐layer patches. In contrast, the Ag_(2)_ sample—intercalated into a zero‐layer graphene substrate—shows a dominant Dirac cone from the quasi‐free‐standing mono layer graphene (QFMLG) layer after intercalation, while the weaker BLG feature originates from intercalation within overgrown graphene patches.

Notably, the binding energy of *E*_*D*2_​is approximately 0.1 eV higher than that of *E*_*D*1_, indicating enhanced n‐type doping in the (Ag_(2)_–QFMLG) layer. The charge carrier density (*n*) of the Ag_(2)_–QFMLG layer was estimated using Luttinger's theorem, *n*  =  *A*/π^2^, where A represents the Fermi surface area [84, 85]. Neglecting the trigonal warping of the π* band Fermi pocket, it was approximated as a circle with diameter *k*_F_, corresponding to the peak separation in the MDC curve at the Fermi level (Figure S17e(ii)) for the Ag_(2)_–QFMLG π* bands. With *k*_F_ = 0.274  Å^−1^, this yields *n* ≈ 6.0 × 10^13^cm^−2^, which is higher than the previously reported value for Ag_(1)_–QFMLG [49, 80]. This confirms the stronger n‐doping in the Ag_(2)_–QFMLG layer, resulting from increased charge transfer from the Ag_(2)_ layer to the graphene.

### 1.4 Phase‐Dependent Optical and Nonlinear Properties of 2D Ag

The phase‐engineered intercalation of 2D Ag enables tunable optoelectronic properties, particularly through phase‐dependent optical absorption. To quantify this, we employed spectroscopic imaging ellipsometry (SIE) to extract the complex dielectric functions (ε = ε_1 _+ i ε_2_) of the Ag_(1)_ and Ag_(2)_. SIE measures changes in the polarization state of monochromatic light reflected from the thin film system, yielding ellipsometric angles Ψ and Δ via the complex reflectance ratio ρ =  *r*_p_/*r*_s_ =  tan Ψ* *e* ^*i*Δ^, where *r*_p_ and *r*_s_ are the amplitude reflection coefficients of p‐, and s‐polarized light, respectively [86]. Using a CCD‐camera together with suitable lens in the detection arm of the ellipsometer system, hyperspectral maps of Ψ(λ) and Δ(λ) were obtained with submicron resolution. Figure 4a shows Ψ maps at 450 nm (∼2.76 eV) for Ag_(1)_ and Ag_(2)_ dominant structures highlighting the strong contrast between both phases in the reflection matrix indicating sizeable changes in their respective dielectric response.

**FIGURE 4:**
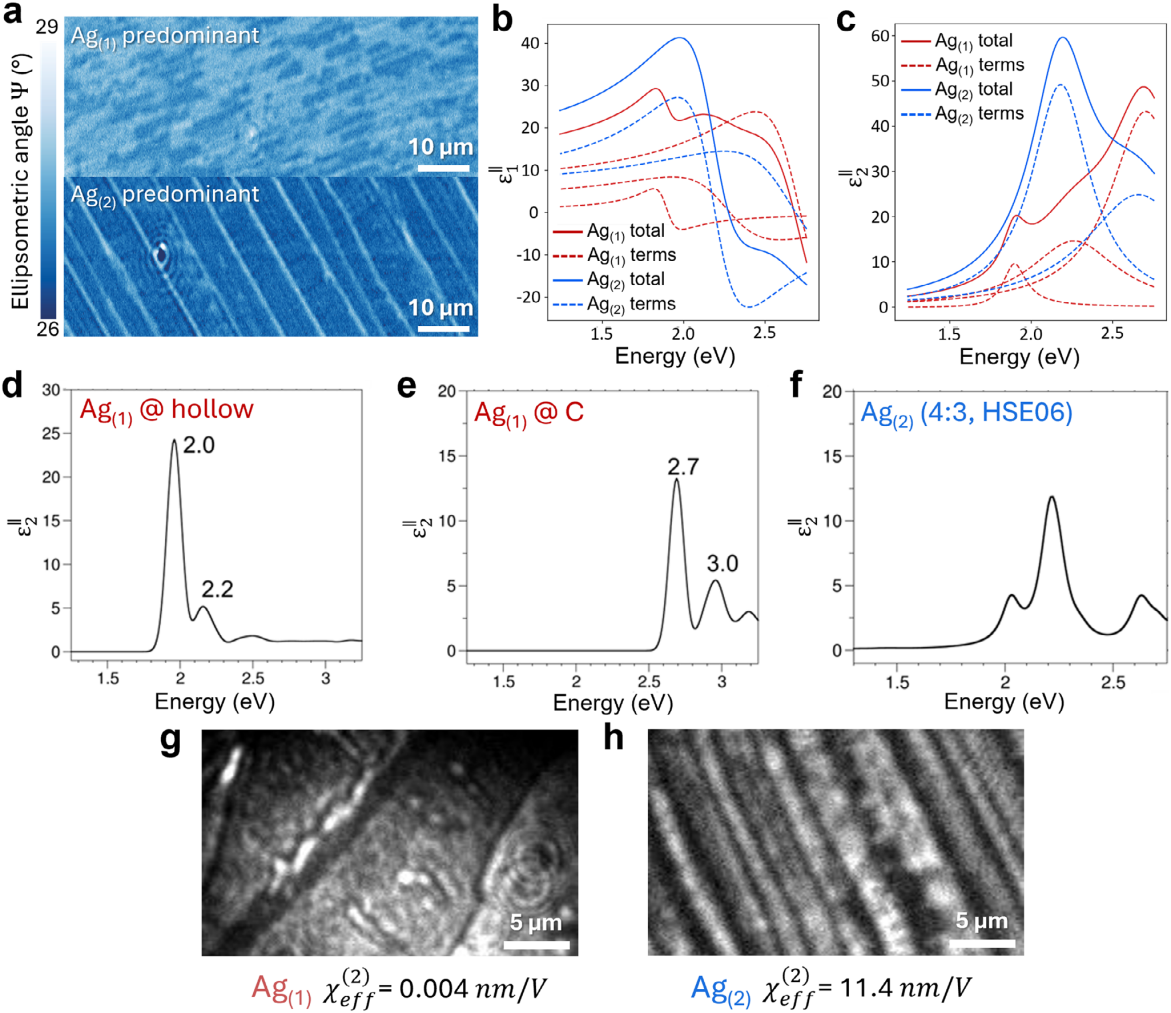
Phase‐engineered optical and nonlinear response in 2D Ag. (a) Spectroscopic imaging ellipsometry (SIE) Ψ‐maps acquired at 450 nm (∼2.76 eV) for Ag_(1)_ and Ag_(2)_ dominant samples, showing high (Ag_(1)_) and low (Ag_(2)_) optical contrast regions. (b) Real (ε_1_) and (c) Imaginary (ε_2_) part of the complex dielectric function (ε = ε_1_ + i ε_2_) obtained from homogenous regions of Ag_(1)_ predominant sample, shown as red solid lines and from homogenous regions of Ag_(2)_ predominant sample, shown as blue solid lines. Individual terms of the parametrized absorption peaks are shown as dashed lines. (d–f) Theoretically calculated optical absorption spectra for (d) Ag_(1)_ on hollow‐site (Ag_(1)_(h)), (e) Ag_(1)_ on carbon‐site registry (Ag_(1)_(C)) and (f) Ag_(2)_. The optical spectra for Ag_(1)_ (d, e) include excitonic effects within the GW‐BSE approximation, while the optical spectrum for Ag_(2)_ (f) is estimated using density‐functional theory within the HSE06 approximation, applied to the 4:3 model for Ag_(2)_. Second harmonic generation (SHG) microscopy images of (g) Ag_(1)_ and (h) Ag_(2)_ dominant samples, respectively, with their extracted effective second order susceptibility $\left(\chi_{eff}^{\left(2\right)}\right)$ values, showing strong phase‐dependent contrast in nonlinear optical response. These results demonstrate that defect‐templated phase selection directly enables tunable linear and nonlinear optical functionality at atomic thickness.

Ellipsometric spectra (Ψ(λ), Δ(λ)) extracted from representative regions of the Ag_(1)_ dominant sample were fitted using a multilayer optical model using regression analysis (Figure S18). Very similar results are obtained for the Ag_(2)_ sample following the same approach. The resulting dielectric functions (ε), shown in Figure 4b,c, display clear differences between the real (ε_1_) and imaginary part (ε_2_) of the dielectric function between Ag_(1)_ and Ag_(2)_. In the loss term (ε_2_), Ag_(1)_ exhibits a strong interband resonance at 2.7 eV (∼460 nm) and two weaker peaks at 1.9 and 2.3 eV, whereas Ag_(2)_ shows a dominant feature at 2.2 eV (∼560 nm) and a smaller peak near 2.7 eV. Although the resonance positions and relative spectral shapes in ε_2_ are robust, the absolute magnitude and baseline of the extracted dielectric response should be interpreted with care. This is due to several limitations in the analysis of ultrathin layers. A comprehensive discussion can be found in the Supporting Information.

We perform state‐of‐the‐art calculations for the optical spectra in the Ag_(1)_ system, including the effects of electron‐hole interactions for the 2D semiconductor, using the GW‐BSE approximation [87]. The calculated ε_2_ spectrum is shown in Figure 4d for Ag_(1)_ at the hollow‐site registry (Ag_(1)_(h)), and in Figure 4e for Ag_(1)_ at the carbon‐site registry (Ag_(1)_(C)) (see Figure S8). Prominent excitonic peaks are observed at ∼ 2.0 and ∼ 2.7 eV for Ag_(1)_(h) and Ag_(1)_(C), respectively. The theoretically‐predicted exciton resonances are consistent with the peaks obtained from ellipsometry—a strong interband resonance at 2.7 eV (attributed to Ag_(1)_(C)), and weaker peaks at 1.9 and 2.3 eV (attributed to Ag_(1)_h)). This agreement confirms the coexistence of both registries in Ag_(1)_, consistent with their similar formation energies (Figure 2f and Figure S8). We note that the electron‐hole interactions dramatically change the low‐energy optical spectra (Figure S19), due to the semiconducting and confined nature of the 2D Ag, which has an indirect quasiparticle gap of ∼ 1.0 eV in both registries (Figure S20, Table S3), in stark contrast to other 2D metals grown by CHet, such as 2D Ga [58], which is metallic. The interband contributions to the lowest‐energy exciton resonances are shown in Figure S19 – that in Ag_(1)_(h) arises mainly from transitions between the highest occupied and lowest unoccupied bands at the K and M pockets of the Brillouin zone while that in Ag_(1)_(C) arises due to transitions between the highest occupied and lowest unoccupied bands localized mainly close to K. The differences observed in the exciton energies partially reflects the differences in band gaps at the K point—∼2.6 eV for Ag_(1)_(h) and ∼ 3.1 eV for Ag_(1)_(C) structures.

For Ag_(2)_, we provide an estimate for the optical absorption spectra by using as a model the 4:3 Ag:SiC structure (2 × 2 Ag on √3 × √3 SiC) [74] that approximates the electronic environment of (27:25) Ag_(2)_ while maintaining the 30° lattice alignment. We do not take into account excitonic effects in predicting the optical spectra of Ag_(2)_, but use standard density functional theory (DFT) with the hybrid HSE06 exchange‐correlation functional, which is commonly used to estimate optical gaps (see also Figure S21) [88, 89]. Although we do not expect the predicted optical absorption spectrum to quantitatively describe the measured ε_2_, the peak positions obtained using this approach can be indicative of the experimental features, and the predicted peak at ∼ 2.2 eV is in reasonable agreement with experiment (Figure 4c).

Beyond linear optics, the broken inversion symmetry at the SiC/Ag interface enables strong second‐order [90] and higher‐order [91] nonlinear optical responses unlike bulk Ag. Figure 4g presents second harmonic generation (SHG) microscopy images of Ag_(1)_ and Ag_(2)_ dominant samples, with step and terrace morphology. From the intensity of the images, the extracted sheet susceptibilities ($\chi_{sheet}^{\left(2\right)}$) are 0.001 ± 0.0001 nm^2^/V for Ag_(1)_ and 3.2 ± 0.08 nm^2^/V for Ag_(2)_, corresponding to effective susceptibilities ($\chi_{eff}^{\left(2\right)}$) of 0.004 ± 0.0005 and 11.4 ± 0.3 nm/V, respectively, based on monolayer thickness from TEM (Figure 3c,d) using the sheet model [92]. Ag_(2)_’s $\chi_{eff}^{2}$ is comparable to values for 2D Ga and In [90], while Ag_(1)_ shows a more than three‐orders‐of‐magnitude lower response. This stark difference is attributed to stronger Ag–Si interactions in Ag_(1)_, which stiffen the electron cloud and reduce polarizability. These results reveal that precise defect engineering not only governs 2D Ag phase formation but also modulates optical absorption, nonlinear susceptibility, and polarization response—providing a materials platform that could enable atomically thin metasurfaces and reconfigurable nanophotonic components.

## 2 Conclusion

Phase control in atomically thin metals is inherently challenging because small energetic differences between competing structures are amplified under confinement, while conventional growth‐parameter tuning offers limited and system‐dependent selectivity. Here, we demonstrate that defect‐templated confinement heteroepitaxy provides a general and reproducible strategy to overcome this limitation by treating interfacial defects as deliberate design elements rather than unavoidable disorder. Using silver confined at the graphene/SiC interface as a model system, we establish a direct relationship between graphene defect chemistry and the stabilization of distinct crystalline phases of monolayer Ag.

By engineering vacancy‐, line‐, and sp^3^‐type defects in the graphene overlayer, we achieve near‐deterministic selection between a kinetically favored near‐commensurate Ag_(1)_ phase and a thermodynamically preferred, denser Ag_(2)_ phase. Multimodal characterization and first‐principles calculations reveal that this selectivity arises from a competition between defect‐mediated nucleation pathways and phase‐dependent packing energetics under confinement, demonstrating that graphene acts as an active template governing phase evolution rather than a passive encapsulant.

Importantly, defect‐programmed phase control directly translates into functional consequences. The Ag_(1)_ and Ag_(2)_ phases exhibit distinct electronic coupling to graphene, phase‐dependent charge transfer, and strongly contrasting linear and nonlinear optical responses, including a five‐order‐of‐magnitude difference in second‐harmonic generation. Together, these results show that interfacial defect engineering can encode optical and electronic functionality at atomic thickness without lithographic patterning or postgrowth modification.

Preliminary experiments on confined 2D Ga provide additional evidence that this concept extends beyond Ag. Oxygen‐plasma‐treated epitaxial graphene produces mixed‐phase or predominantly Ga_(1)_ growth, whereas zero‐layer graphene preferentially promotes Ga_(2)_, as identified through their characteristic ultralow‐frequency Raman modes (Figure S22).

More broadly, this work establishes defects as programmable design parameters for controlling crystal phase and emergent functionality in confined two‐dimensional metals. The preliminary Ga results further support the transferability of this framework, although the defect chemistry and growth conditions required for phase selection are expected to remain specific to each metal–substrate system.

## 3 Methods

### 3.1 ZLG and EG Synthesis

Zero‐layer graphene (ZLG) and epitaxial graphene (EG) were synthesized on the Si‐terminated (0001) face of semi‐insulating 6H‐SiC substrates (Coherent Corp.) via thermal silicon sublimation. The substrates were first annealed at 1400°C for 30 min in a 10% H_2_/Ar gas mixture at 700 Torr to remove polishing damage and surface contaminants. ZLG formation was then achieved by annealing the substrates at 1600°C in 700 Torr of pure Ar for 30 min. Partial ZLG coverage was obtained by tuning the annealing temperature and duration. Monolayer EG was synthesized by further annealing at 1800°C in 700 Torr of pure Ar for 20 min.

### 3.2 ZLG Growth by RF Heating in Ar Atmosphere

In this method, ZLG samples were prepared by heating an on‐axis, Si‐terminated, H‐etched 6H‐SiC(0001) (SiCrystal GmbH) substrate in an argon (impurity level < 10 ppm) atmosphere [53] in near‐atmospheric Ar pressure (approx. 600 Torr), with an argon flow rate in the range of 0.2 slm. During annealing, the temperature was maintained at 1444°C for 4 min and 30 s. This yields several‐µm wide terraces, uniformly covered with the (6√3 × 6√3)R30° superstructure characteristic for perfect epitaxy of graphene on SiC.

### 3.3 Plasma Treatment and 2D Silver Intercalation

To facilitate silver intercalation, epitaxial graphene (EG) surfaces were first activated using remote helium plasma treatment in a Pie Scientific Tergeo plasma cleaner. The treatment was carried out under 15 sccm He flow at 6 Torr pressure, 15 W RF power, and a 0.1 duty cycle for 1, 2, or 3‐min. In the case of oxygen plasma treated graphene, monolayer EG samples were subjected to a reactive oxygen plasma etch (150 sccm O_2_, 50 sccm He, 500 mTorr, 50 W, 1 min) using a Tepla M4L plasma chamber.

Silver intercalation was performed in a 1‐inch outer diameter quartz tube furnace (Thermo Scientific Lindberg/Blue M Mini‐Mite). ZLG or plasma‐treated EG/SiC substrates (1 × 0.5 cm^2^) were placed face‐down inside an alumina crucible (Robocasting Enterprises) positioned above 80 mg of high‐purity silver powder (99.99%, Sigma‐Aldrich). The tube was evacuated to ∼ 5 mTorr, leak‐checked, and then filled with Ar to 500 Torr. The system was ramped to 900°C at 50°C/min under continuous Ar flow (50 sccm), held at temperature for 1 h (unless otherwise noted), and then cooled to room temperature under inert atmosphere.

### 3.4 Raman Spectroscopy and Microscopy

Raman spectroscopy was performed using a Horiba LabRAM system equipped with a 532 nm excitation laser and an ultra‐low frequency (ULF) notch filter to suppress the Rayleigh line. For ULF Raman measurements, spectra were acquired using a 300 grooves mm^−1^ grating with a total integration time of 10 s, averaged over two accumulations and a laser power of ∼ 4 mW. Raman mapping was conducted using the SWIFT ultrafast imaging mode with a typical spatial resolution of 0.8 × 0.8 µm^2^ per pixel. A 100× objective (NA = 0.9) was used for all measurements, and spectral calibration was performed using a standard Si reference. All spectra were baseline corrected, and for the ZLG/EG Raman spectrum, an additional SiC background subtraction was applied.

Strain and carrier concentration in graphene were extracted via vector decomposition of the Raman G and 2D peak positions. Following background subtraction and peak fitting, spatial maps of the G and 2D peak frequencies were used to decouple contributions from strain and doping based on their known sensitivities to uniaxial strain and charge carrier density. The analysis employed reported Grüneisen parameters and empirical doping coefficients derived from literature [62, 93, 94]. Data points were plotted in the 2D versus G peak position space, and projections along the iso‐strain and iso‐doping vectors enabled extraction of relative changes in strain (ε) and electron concentration (n).

### 3.5 X‐Ray Photoelectron Spectroscopy

X‐ray photoelectron spectroscopy (XPS) measurements were performed using a Physical Electronics VersaProbe III system equipped with a monochromatic Al Kα x‐ray source (hν = 1486.6 eV) incident at 45° relative to the sample plane. Spectra were acquired using a concentric hemispherical analyzer over a 200 × 200 µm^2^ analysis area. High‐resolution scans of the C 1s, Si 2p, and Ag 3d regions were collected at a pass energy of 29 eV, while the O 1s and Ag MNN Auger regions were recorded at a pass energy of 55 eV. Samples were mounted directly onto a screw‐fastened platen without additional adhesives. Spectra were charge‐referenced by setting the sp^2^‐hybridized carbon (C 1s) peak to 284.5 eV.

### 3.6 Atomic Force Microscopy (AFM)

Atomic force microscopy (AFM) measurements were carried out using a Bruker Dimension Icon system operated in PeakForce Tapping mode. A ScanAsyst‐Air probe was used with a peak force setpoint of ∼ 700 pN. Topography and adhesion maps were acquired at a scan rate of 1.0 Hz.

### 3.7 Low Energy Electron Diffraction (LEED)

Low‐energy electron diffraction (LEED) measurements were performed using a Er‐LEED system (Specs GmbH). Samples previously exposed to ambient conditions were degassed in UHV (base pressure < 5 × 10^−10^  mbar) at 130 °C for 25–30 min to remove surface adsorbates. LEED patterns were acquired at an incident electron energy of 70 eV.

### 3.8 Scanning Transmission Electron Microscope (STEM)

Cross‐sectional lamellae were prepared using a focused ion beam (FIB) lift‐out technique in a dual‐beam system (Helios Nanolab 660, Thermo Fisher Scientific). Rough trenching to isolate the region of interest was performed at 30 kV with a beam current of 9 nA. Subsequent thinning was carried out at 30 kV and 0.79 nA, followed by final polishing at 5 kV and 0.36 nA to minimize surface damage and amorphization.

High‐angle annular dark‐field scanning transmission electron microscopy (HAADF‐STEM) imaging and energy‐dispersive x‐ray spectroscopy (EDS) elemental mapping were conducted using a double aberration‐corrected FEI Titan3 G2 microscope operated at 300 kV. The HAADF detector had a collection angle of 52–253 mrad. EDS maps were acquired using a SuperX detector in STEM mode.

Image analysis was performed using ImageJ and Gatan DigitalMicrograph. Average intensity line profiles were extracted from selected regions of interest (ROI) across the cross‐sections to quantify the interlayer spacing between the Ag and graphene layers. These profiles were averaged across rectangular windows spanning ∼ 10 nm in the lateral direction to minimize noise and highlight periodic contrast features. Relative intensities in the HAADF images were used as a proxy for atomic number (Z) contrast, allowing estimation of the number of Ag atomic layers based on comparative brightness.

### 3.9 Scanning Tunneling Microscopy (STM) Measurements

Intercalated Ag was investigated using a cryogenic STM system (Createc) operating at 5 K in UHV. The samples were transported in air and degassed at 150°C for 30 min in UHV before transferring into the cold STM head held at 5 K. The topography and differential conductance (dI/dV) spectroscopy were obtained using electrochemically etched W tips. Before measurements, the tips were cleaned by Ne^+^ sputtering and electron beam heating. The tip calibration was carefully done using an Ag (111) crystal until good topography images were achieved and differential tunneling spectroscopy (dI/dV) showed a surface state onset at about −63 mV, typical for Ag. dI/dV spectra were recorded using a lock‐in technique with a peak‐to‐peak bias modulation of 5 mV and a modulation frequency of 675 Hz.

### 3.10 Secondary Electron Microscopy (SEM)

Surface morphology was examined using an Apreo 5 scanning electron microscope (Thermo Fisher Scientific) operated in immersion mode. Secondary electron (SE) imaging was performed using the Through‐Lens Detector (TLD). Imaging was conducted at magnifications of 2000× to 10 000×, with an accelerating voltage of 2.00 kV, beam currents ranging from 0.4 to 3.2 nA, and working distances between 2 and 4 mm.

### 3.11 Auger Electron Spectroscopy (AES) Mapping

AES measurements were performed using a Physical Electronics VersaProbe III system equipped with a LaB_6_ scanning electron microscopy column (1–10 keV) and a concentric hemispherical analyzer. A monochromatic Al Kα X‐ray source (hν = 1486.6 eV) was used for XPS‐based height alignment, ensuring optimal positioning relative to the analyzer prior to AES analysis. The SEM column and analyzer were oriented at 45° takeoff angles with respect to the sample plane and rotated 90° relative to one another. Auger spectra were acquired using a 10 keV, 2 nA electron beam with an estimated probe diameter of ∼200 nm. The analyzer was operated in Fixed Retard Ratio (FRR) Transmission mode.

Spot and line scans were collected in scanned mode. Postacquisition data processing involved signal differentiation and smoothing using a 5‐point Savitzky–Golay filter in *CasaXPS* [95]. Elemental quantification was guided by relative sensitivity factors extracted from XPS spectra collected over the same ∼ 20 µm region. Auger mapping was conducted in unscanned mode using Physical Electronics’ three‐point intensity calibration. This qualitative method involves subtraction of a linear background across two energy intervals and intensity averaging over a third peak region to yield time‐normalized signals. For energy calibration, absolute reference values were taken from Seah et al. [96].

### 3.12 Angle‐Resolved Photoelectron Emission Spectroscopy (ARPES)

ARPES measurements were performed using both lab‐based and synchrotron‐based systems to probe the graphene π‐bands and the electronic band structure of 2D Ag. Prior to the measurement, samples previously exposed to ambient air were degassed at 130°C for 25–30 min in UHV (base pressure < 5 × 10^−^^10^ mbar) to remove surface contaminants. Lab‐based ARPES measurements were performed at room temperature using a NanoESCA system (Scienta Omicron GmbH) operating in momentum microscopy mode with an extractor voltage of 12 kV. During measurement, single‐shot, constant‐energy cuts in momentum space (k‐space) were obtained from ∼ 20 × 15 *µ*m^2^ area of the sample surface, controlled by an adjustable iris aperture. Nonmonochromatized, unpolarized He Iα radiation (hν = 21.22 eV) served as the excitation source. The spectrometer was operated with a k‐space field of view of 4.3 Å^−^^1^, enabling full access to the photoemission horizon up to the Fermi level for this photon energy.

High‐resolution ARPES measurements were performed at the Bloch beamline of the MAX IV synchrotron (Lund, Sweden) using a DA30‐L hemispherical analyzer (Scienta Omicron GmbH). The sample temperature during these measurements was maintained at approximately 20 K. For excitation, photon energies of 40 110, and 180 eV with linear horizontal polarization were employed. The total energy resolution, including contributions from both the analyzer and the beamline optics, ranged between ∼ 17 and ∼ 60 meV depending on the photon energy and experimental configuration. Data were analyzed using Igor Pro 6.37 software.

### 3.13 Spectroscopic Imaging Ellipsometry (SIE)

SIE measurements are performed in ambient conditions with a customized EP4 ellipsometer (Park Systems GmbH). Tunable, monochromatic illumination is achieved by a supercontinuum white light laser in combination with acousto‐optic tunable filters, yielding a spectral linewidth on the order of 5 nm. The signal is imaged onto a CCD camera using a 50x objective with NA = 0.45, which results in a lateral resolution of ∼ 1 *µ*m.

Data is obtained at a fixed angle of incidence of 50° in rotating‐compensator ellipsometry (RCE) mode, and with a spectral range of 450–1000 nm. Data points are spaced 5 nm apart for a total of 111 points. A beam cutter is included in the excitation path to suppress backside reflections from the transparent substrate. The measured spectroscopic maps are manually corrected for small sample movement during the measurement due to for example vibrations. Then, suitable homogeneous regions of interest (ROIs) are chosen to average over, resulting in ellipsometric spectra *Ψ*(*λ*), Δ(*λ*). To determine the complex dielectric function for Ag_(1)_ and Ag_(2)_, an optical multilayer model is constructed and fit to the spectra via regression analysis (see supplement for a detailed description).

### 3.14 SHG

Polarization‐resolved SHG microscopy was implemented following the method from Steves et al. [90]. 800‐nm pulses with 30‐nm bandwidth were focused onto the sample with a 0.4 NA aspheric lens from a Ti:sapphire laser (Vitara, Coherent). Polarization of the excitation was adjusted with a halfwave plate placed before the sample, while the emission from the sample was collected with a 1.25 NA oil immersion objective (Nikon). The SHG signal was isolated from the fundamental and multiphoton photoluminescence using a combination of 400‐nm bandpass and 650‐nm short‐pass filters. An analyzer was placed before the detector to determine the polarization of the emission. An electron‐multiplying CCD and coupled spectrometer (iXion Ultra 897/Shamrock 303i, Andor) were used to record the SHG image and spectra.

Absolute sheet susceptibilities ($\chi_{sheet}^{\left(2\right)}$) were extracted from calibrated CCD photon counts, and effective susceptibilities ($\chi_{eff}^{\left(2\right)}$) were calculated using monolayer thickness from TEM in Figure 3c,d, yielding $\chi_{sheet}^{\left(2\right)}$ = 0.001 ± 0.0001 nm^2^/V and $\chi_{eff}^{\left(2\right)}$= 0.004 ± 0.0005 nm/V for Ag_(1)_, and $\chi_{sheet}^{\left(2\right)}$= 3.2 ± 0.08 nm^2^/V and $\chi_{eff}^{\left(2\right)}$= 11.4 ± 0.3 nm/V for Ag_(2)_ as per Steves et al. [90].

### 3.15 Thermodynamic Calculations

The relative phase stability, and the Ag cluster calculations are done by density functional theory (DFT) as implemented in VASP [97, 98, 99, 100] at the PBE‐level [101] with 450 eV cutoff energy, electronic self‐consistent convergence at 1.4 × 10^−8^ eV/atom, and residual force convergence at 0.01 eV/Å on atoms with D3 van der Waals correction [102, 103]. The k‐point samplings are Γ‐centered 13 × 13 × 1 for 1 × 1 SiC unit cells, 8 × 8 × 1 for 3 × 3 SiC unit cells, 3 × 3 × 1 for 5 × 5 SiC unit cells, and Γ‐only for 10 × 10 SiC unit cells. The cell dimension in the z‐direction is 20Å. Three layers of SiC are included in the phase stability calculations, while two layers of SiC are included in the cluster calculation. All SiC systems are terminated with H atoms at the opposite site of the Ag layer. The structure visualization is made with VESTA [104].

### 3.16 Calculation for Optical Properties

The optical absorption spectra for the Ag_(1)_ phase were obtained with GW‐BSE calculations using the BerkeleyGW code [87], on top of DFT calculations performed using Quantum ESPRESSO [105]. The DFT starting points utilized the local density approximation for the exchange correlation functional. Optimized norm‐conserving Vanderbilt (ONCV) pseudopotentials [106, 107] were used, with a plane wave basis set cutoff of 90 Ry. An energy threshold of 10^−10^ Ry was used to converge the electronic cycles. The Ag_(1)_ phase was modeled with a six‐layered slab of SiC with one layer of Ag atoms stacked over the Si face at the hollow‐ and C‐site registries, while the dangling bonds on the bottom C‐face were passivated with hydrogen atoms. A vacuum spacing of ∼ 18 Å was used to reduce interactions between the periodic slabs. A uniform 18 × 18 × 1 𝑘 ‐grid was found to be sufficient to converge the total energy. A slab Coulomb truncation approach [108] was used in GW calculations. For calculating the dielectric matrix, we use a cutoff of 40 Ry and a summation over ∼ 1400 conduction bands. The quasiparticle energies were obtained using 1400 conduction bands for the summation of the self‐energy for 15 valence and 6 conduction bands over an 18 × 18 × 1 𝑘‐point mesh. The electron‐hole interaction kernel was calculated using an 18 × 18 × 1 𝑘‐point mesh, which was then interpolated onto a fine 54 × 54 × 1 𝑘 ‐grid for 6 valence and 4 conduction bands, that were used to obtain a converged excitonic absorption spectra.

For the Ag_(2)_ phase, we obtained the optical absorption spectra using the independent particle approximation within DFT. The results in Figure 4 are obtained using the 4:3 Ag:SiC structure and the HSE06 exchange‐correlation functional. The DFT transition dipole matrix elements and energy eigenvalues were calculated on a 36 × 36 × 1 𝑘‐point mesh with PBE. HSE06 calculations were performed on a coarser 6× 6 × 1 𝑘‐point mesh, and a 3 × 3 × 1 q‐point mesh for calculating the Hartree‐Fock exchange. Incorporating these corrections in the eigenvalues and rescaling of the transition dipole matrix elements, we obtained the HSE06 optical spectra for the 4:3 Ag:SiC structure, using the 6 × 6 × 1 𝑘‐point mesh. We investigated the effect of the choice of structural models by comparing the optical absorption spectra the 4:3 Ag:SiC structure and the 27:25 Ag:SiC, using the PBE exchange‐correlation functional (Figure S21). For the 27:25 Ag:SiC structure, the optical spectra was calculated using a 6 × 6 × 1 𝑘‐point mesh.

## Conflicts of Interest

The authors declare no conflicts of interest.

## Supporting information




**Supporting File**: adma74689‐sup‐0001‐SuppMat.pdf.

## Data Availability

The data that support the findings of this study are available from the corresponding author upon reasonable request.
