# Supplementary material for: Defect‐Templated Phase Engineering in Atomically Thin Metals
Source: Adv Mater. 2026 Aug 19;38(58):e74689. doi: 10.1002/adma.74689 (PMC13674628; doi:10.1002/adma.74689)
Supplement: Supplementary file 1 [file ADMA-38-e74689-s001.pdf]

Supporting Information

Defect-Templated Phase Engineering in Atomically Thin Metals

Arpit Jain[1], Boyang Zheng[2,3], Sawani Datta[4], Kanchan Ulman[5], Jakob Henz[6], Matthew Wei-Jun Liu[1], Van Dong Pham[7], Wen He[5,8], Chengye Dong[1,3,9], Li-Syuan Lu[1], Alexander Vera[1], Nader Sawtarie[10], Wesley Auker[11], Ke Wang[11], Bob Hengstebeck[11], Zachary W. Henshaw[12], Shreya Mathela[13], Maxwell Wetherington[11], William H. Blades[12], Kenneth Knappenberger[13], Ursula Wurstbauer[6], Su Ying Quek[8,14,15], Ulrich Starke[4], Shengxi Huang[16], Vincent H. Crespi[2], and Joshua A. Robinson*[,1,8,17]

[1]Department of Materials Science and Engineering, The Pennsylvania State University, University Park, PA 16802, USA

[2]Department of Physics, The Pennsylvania State University, University Park, PA 16802, USA

[3]2-Dimensional Crystal Consortium, The Pennsylvania State University, University Park, PA 16802, USA

[4]Max-Planck-Institut für Festkörperforschung, Heisenbergstraße 1, 70569 Stuttgart, Germany

[5]Department of Physics, National University of Singapore, Singapore 117551

[6]Institute of Physics and Center for Soft Nanoscience (SoN), University of Münster, 48149 Münster, Germany

[7]Paul-Drude-Institut für Festkörperelektronik, Hausvogteiplatz 5-7, Leibniz-Institut im Forschungsverbund Berlin e. V., 10117 Berlin, Germany

[8]Department of Materials Science and Engineering, National University of Singapore, Singapore 117575, Singapore

[9]Center for 2-Dimensional and Layered Materials, The Pennsylvania State University, University Park, PA 16802, USA

[10]Department of Chemical and Petroleum Engineering, University of Pittsburgh, Pittsburgh, Pennsylvania 15260, USA

[11]Materials Research Institute, The Pennsylvania State University, University Park, PA 16802, USA

[12]Department of Physics and Engineering Physiscs, Juniata College, Huntingdon 16652, Pennsylvania, USA

[13]Department of Chemistry, The Pennsylvania State University, University Park, PA 16802, USA

[14]Centre for Advanced 2D Materials, National University of Singapore, Singapore 117542, Singapore

[15]NUS Graduate School, Integrative Sciences and Engineering Programme, National University of Singapore, Singapore 117456, Singapore

[16]Department of Electrical and Computer Engineering and the Rice Advanced Materials Institute, Rice University, Houston, TX, USA

[17]Center for Atomically Thin Multifunctional Coatings, The Pennsylvania State University, University Park, PA 16802, USA

[*] Corresponding author: jar403@psu.edu

Additional X-ray Photoelectron Spectroscopy (XPS) Characterization for Gr/Ag/SiC

The structural evolution of the 2D Ag samples grown through the confinement heteroepitaxy (CHet) method can be monitored through changes in the high-resolution C 1s and Si 2p core levels measured by x-ray photoelectron spectroscopy (XPS) (**Fig S1**). Prior to intercalation, the zero-layer graphene (ZLG) sample displays a characteristic C 1s spectrum (**Fig S1a**) with SiC substrate peaks at 283.6 eV and buffer-layer S1 and S2 features at 284.6 eV and 285.2 eV, respectively. Additional sp^2 carbon and oxygen-functionalized C–O (286.8 eV) and C=O (288.3 eV) peaks reflect the presence of overgrown terrace-edge EG and partial oxidation of the buffer. Pristine epitaxial graphene (EG) samples show (**Fig S1b**) strong sp^2 graphene peaks alongside residual buffer components and the SiC substrate signal, while oxygen plasma treatment introduces prominent C–O and C=O features (**Fig S1c**), indicating surface functionalization.[1]

Following Ag intercalation, the C 1s spectra exhibit clear signatures of successful buffer layer decoupling and Ag incorporation. Both ZLG and oxygen-plasma-treated EG samples show significant reduction in the S1 and S2 buffer peaks, complete disappearance of the oxygenated carbon signals due to defect healing,[1] and increased intensity of the sp^2 peak. Notably, the SiC substrate peak shifts by ~1 eV to lower binding energy (282.6 eV), consistent with interfacial band bending.[1,2] These changes confirm the formation of an additional quasi-freestanding monolayer graphene (QFMLG) and successful intercalation of Ag at the graphene/SiC interface, further corroborated by Raman mapping (**Figure 1d–f**). In contrast, pristine EG samples exhibit incomplete intercalation, with persistent S1/S2 buffer peaks and both band-bended (SiC peak at 282.6 eV)) and bulk SiC’ (283.6 eV, unintercalated regions of SiC without band bending) peaks, suggesting coexistence of intercalated and non-intercalated regions.[1,3]

The Si 2p core-level evolution complements C 1s results and highlights the interfacial effects of Ag intercalation. All three starting samples display substrate (101.4 eV), surface (101.9 eV), and minor SiO_x (102.6 eV) peaks (**Figure S1d–i**). After intercalation, ZLG and plasma-treated samples reveal a distinct Ag–SiC signal shifted to ~100.3 eV, attributed to downward band bending from interfacial Ag. A separate SiC’ component at 101.0 eV corresponds to regions without Ag, while the SiO_x peak persists due to substrate defects. For pristine EG, the band-shifted Ag–SiC peak appears significantly weaker relative to the unshifted SiC’ signal, reinforcing that intercalation is incomplete in this case. Together, the C 1s and Si 2p analyses establish a strong spectroscopic framework for evaluating intercalation quality and structural transformation in 2D Ag heterostructures.

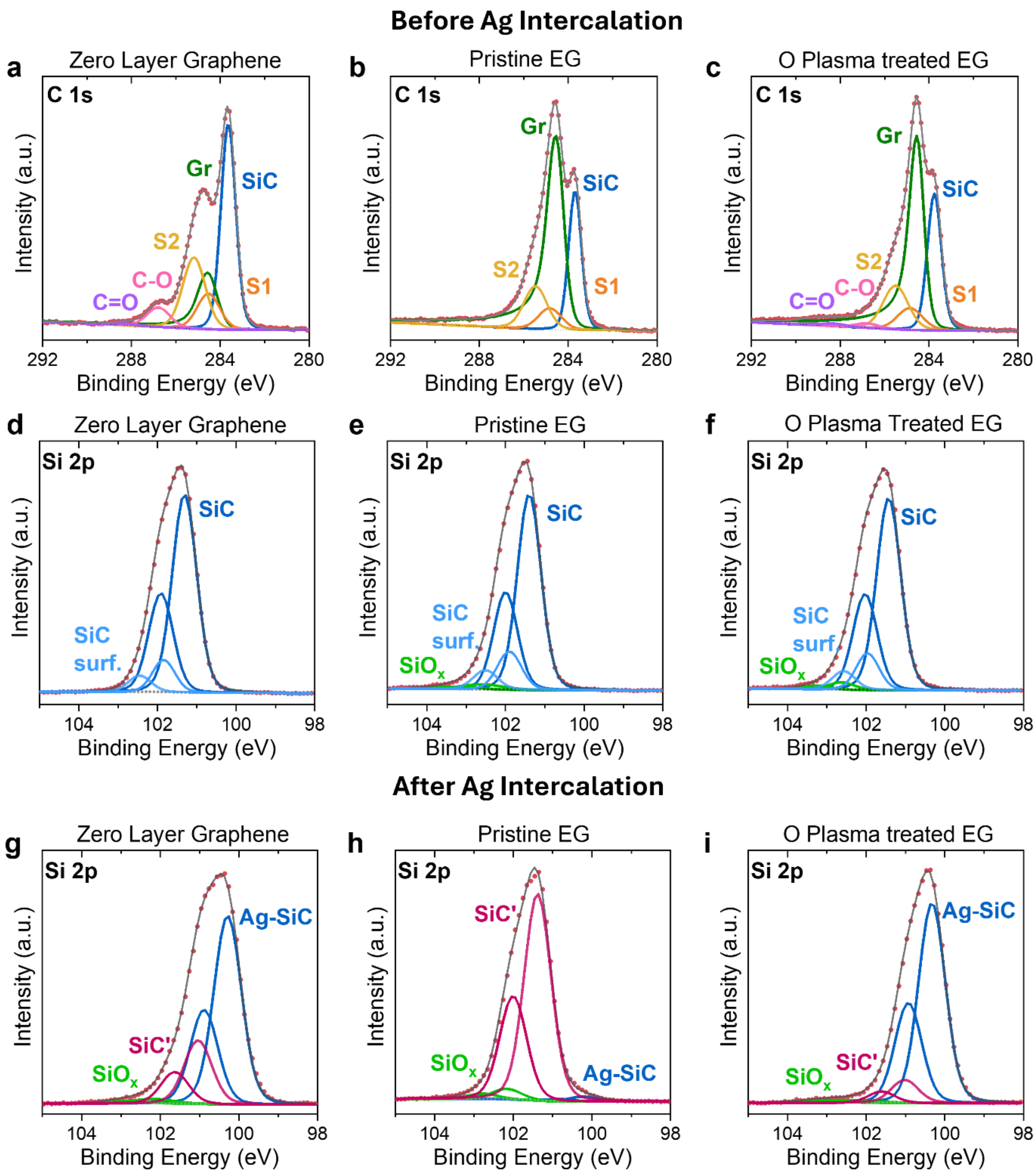

Figure S1. Core level XPS analysis for CHet evolution of EG/Ag/SiC. High-resolution **(a-c)** C 1s and **(d-f)** Si 2p spectra for ZLG, pristine EG, and O plasma-treated EG before Ag intercalation. Corresponding Si 2p spectra after intercalation are shown in **(g–i)** for the same three samples. The spectra reveal interfacial band bending, buffer layer decoupling, and intercalation quality through characteristic shifts and intensity changes in SiC, buffer, and oxide-related peaks.

Silver XPS and Auger Characterization of Intercalated 2D Ag

We used Ag 3d core-level X-ray photoelectron spectroscopy (XPS) and Ag MNN Auger spectroscopy to confirm the presence of intercalated 2D silver in Gr/Ag/SiC heterostructures. As a reference, we first measured a sputter-cleaned bulk silver film (**Figs. S2a–b**), which exhibited an Ag $3d_{5/2}$ peak at 368.3 eV, a spin–orbit doublet separation of 6 eV, and a plasmon loss feature at 372.2 eV—values consistent with bulk metallic Ag.[4] The Ag MNN Auger peak appeared at 1128.8 eV binding energy (corresponding to 357.8 eV kinetic energy using a 1486.6 eV excitation source).

To evaluate the bonding character of intercalated silver, we calculated the Auger parameter, defined as the sum of the Ag $3d_{5/2}$ binding energy and the Ag MNN kinetic energy. Pristine silver yields a parameter >725 eV, whereas compound or oxidized Ag results in a lower value.[4] After silver intercalation, all three sample types—ZLG, pristine EG, and oxygen plasma-treated EG—show Ag $3d_{5/2}$ peaks centered at 368.5 eV and a distinct shoulder at ~368.9 eV and an absence of the bulk silver plasmon loss feature (**Figs. S2c–e**). We assign the main Ag^0 peak to in-plane Ag–Ag bonding, and the higher binding energy shoulder to out-of-plane Ag–Si covalent interactions. Corresponding Ag MNN peaks (**Figs. S2f–h**) in all samples yield Auger parameters exceeding 725.5 eV.

Among the three intercalated systems, the pristine EG sample exhibited the lowest XPS signal intensity and signal-to-noise ratio, indicating a reduced intercalation efficiency compared to ZLG and oxygen-functionalized EG. This trend further corroborates the correlation between graphene defect density and intercalation quality, as detailed in the main text.

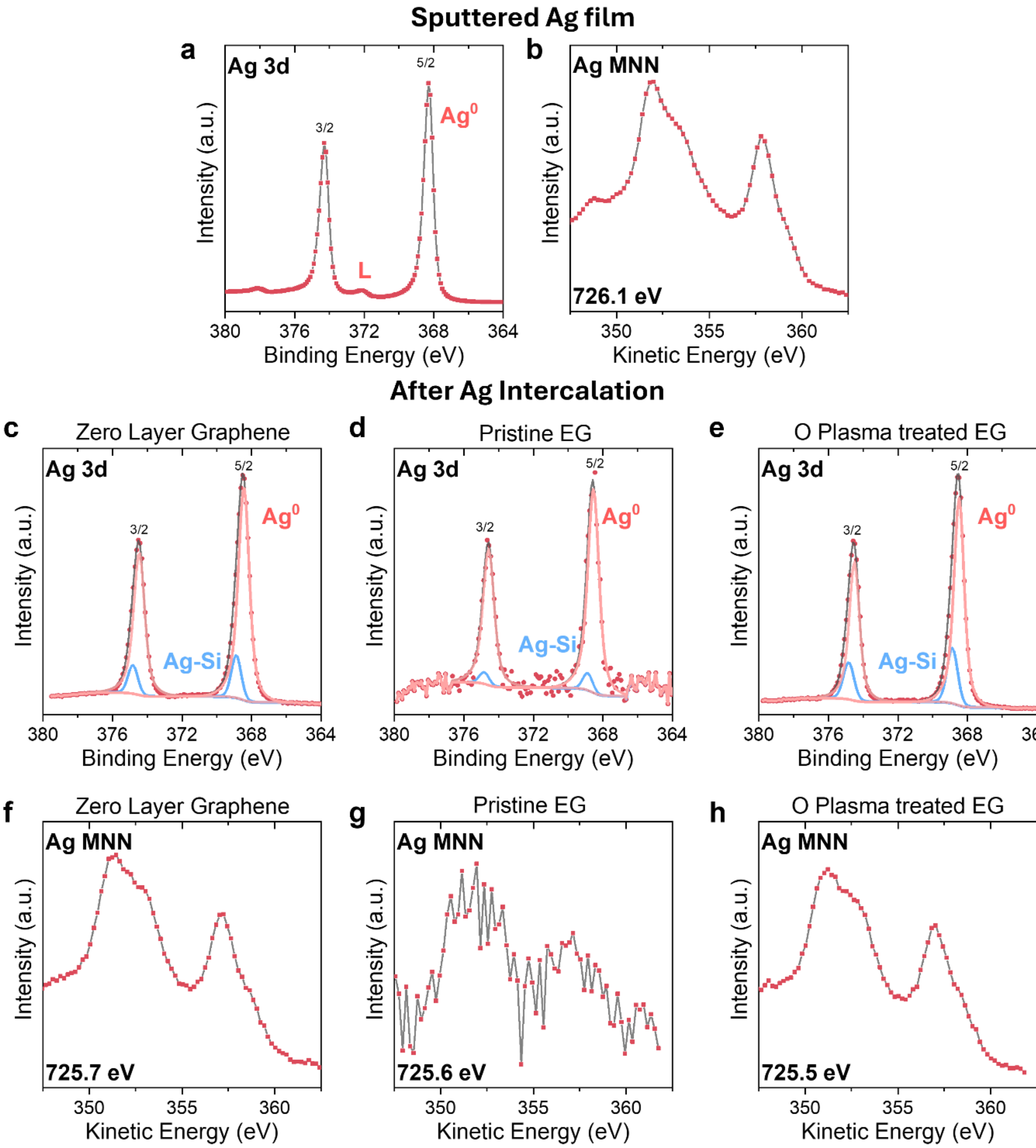

Figure S2. Ag 3d XPS and Ag MNN Auger spectra for intercalated Ag in Gr/Ag/SiC system. (a) High-resolution Ag 3d XPS and **(b)** Ag MNN Auger spectra for a reference Ag foil after Ar^{+} ion sputtering. High-resolution **(a-c)** Ag 3d XPS and (**f-h)** MNN Auger spectra for intercalated silver in **(c, f)** ZLG, **(d, g)** pristine EG, and **(e, h)** oxygen plasma-treated EG samples, respectively.

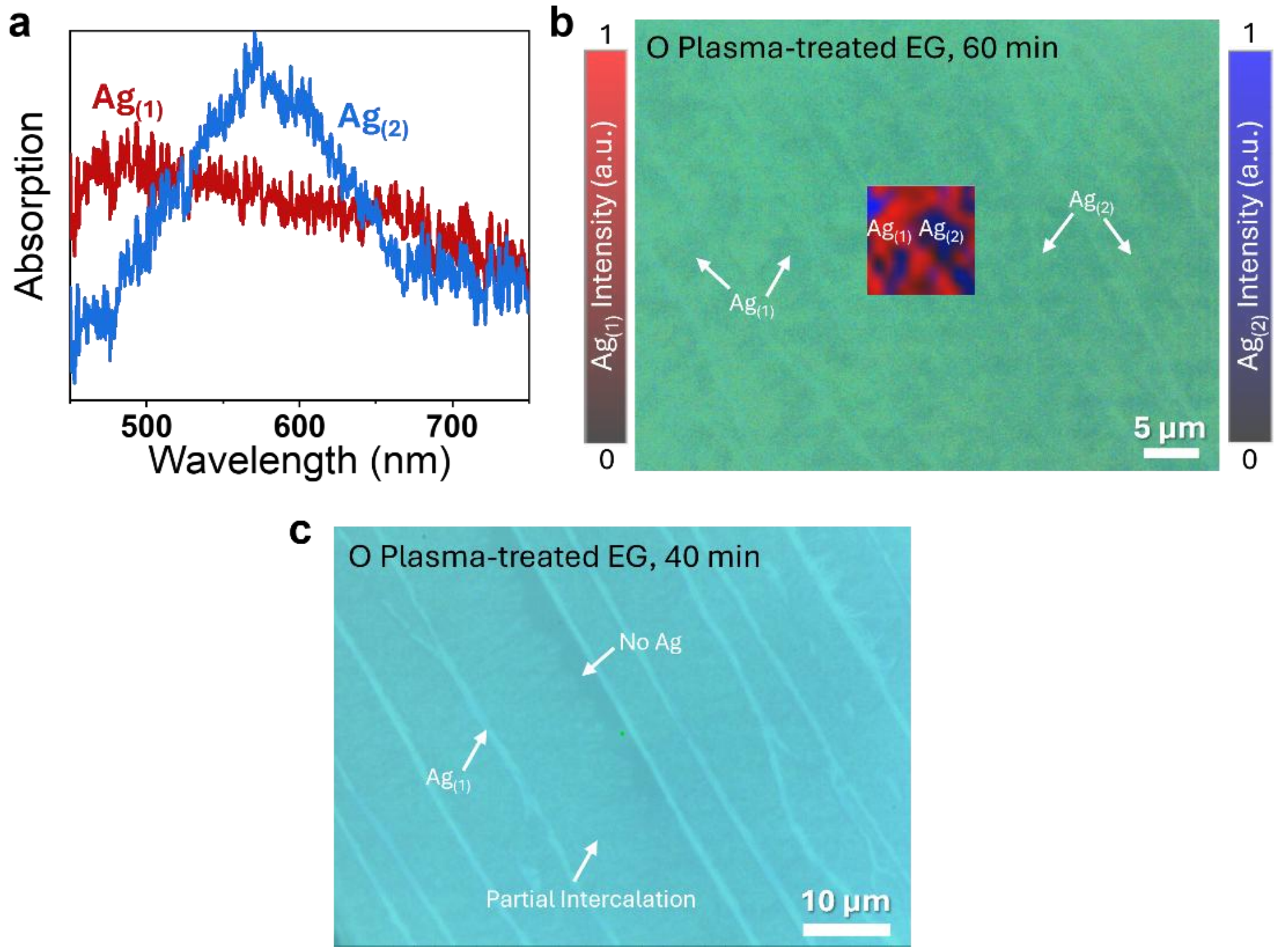

Figure S3. Phase-dependent optical absorption and contrast in 2D Ag. (a) White-light differential reflection spectra ($\frac{\Delta R}{R}(\omega) = \frac{R(\omega)-R_0(\omega)}{R_0(\omega)}$), for $Ag_{(1)}$ and $Ag_{(2)}$, directly reflecting optical absorption. Both spectra show interband transitions in good agreement with Zhang et al.,[9] with experimental and theoretical details described previously in their work.[9] **(b)** Optical micrograph of fully intercalated 2D Ag sample (60-minute intercalation) on oxygen plasma-treated EG. Inset shows 10×10 µm^2 Raman map of the ULF region, with mapped Raman intensity corresponding to $Ag_{(1)}$ Raman intensity map highlighting the $Ag_{(1)}$ modes (16, 18.5, 25 cm^{-1}, shown in red) and $Ag_{(2)}$ modes (69, 81, 91 cm^{-1}, shown in blue). Color bars indicate the relative intensity of each phase's Raman features. (c) Optical micrograph of a partially intercalated sample (40-minute intercalation) on oxygen plasma-treated EG, with regions labeled for $Ag_{(1)}$, partially intercalated, and unintercalated areas.

Silver intercalation on Zero Layer Graphene prepared in Ultra High Vacuum (UHV)

ZLG samples grown in Ar atmosphere in the RF-furnace at MPI-Germany, provide a contrasting platform to ZLG synthesized at PSU. Raman spectra (**Figure S4a**) of the RF-furnace ZLG, after subtracting the SiC background, reveal characteristic signatures of both ordered buffer (sharp 1500 cm^{-1} peak) and disordered buffer regions (broad bands at ~1350 cm^{-1} and 2500–3000 cm^{-1}) as reported by Turker *et al.*,[5] indicating lateral phase inhomogeneity within the laser spot size (~1 µm).

Raman mapping analysis confirms the presence of intercalated silver beneath the RF furnace-grown ZLG. As shown in **Figure S4b**, CHet-grown Ag beneath the RF-furnace grown ZLG sample from MPI exhibits coverage exclusively by the $Ag_{(2)}$ phase, in contrast to pure $Ag_{(1)}$ growth reported for MBE-based intercalation in UHV conditions.[6] Dark contrast regions in the optical image align with undergrown SiC terraces, where neither graphene growth nor Ag intercalation occurs.

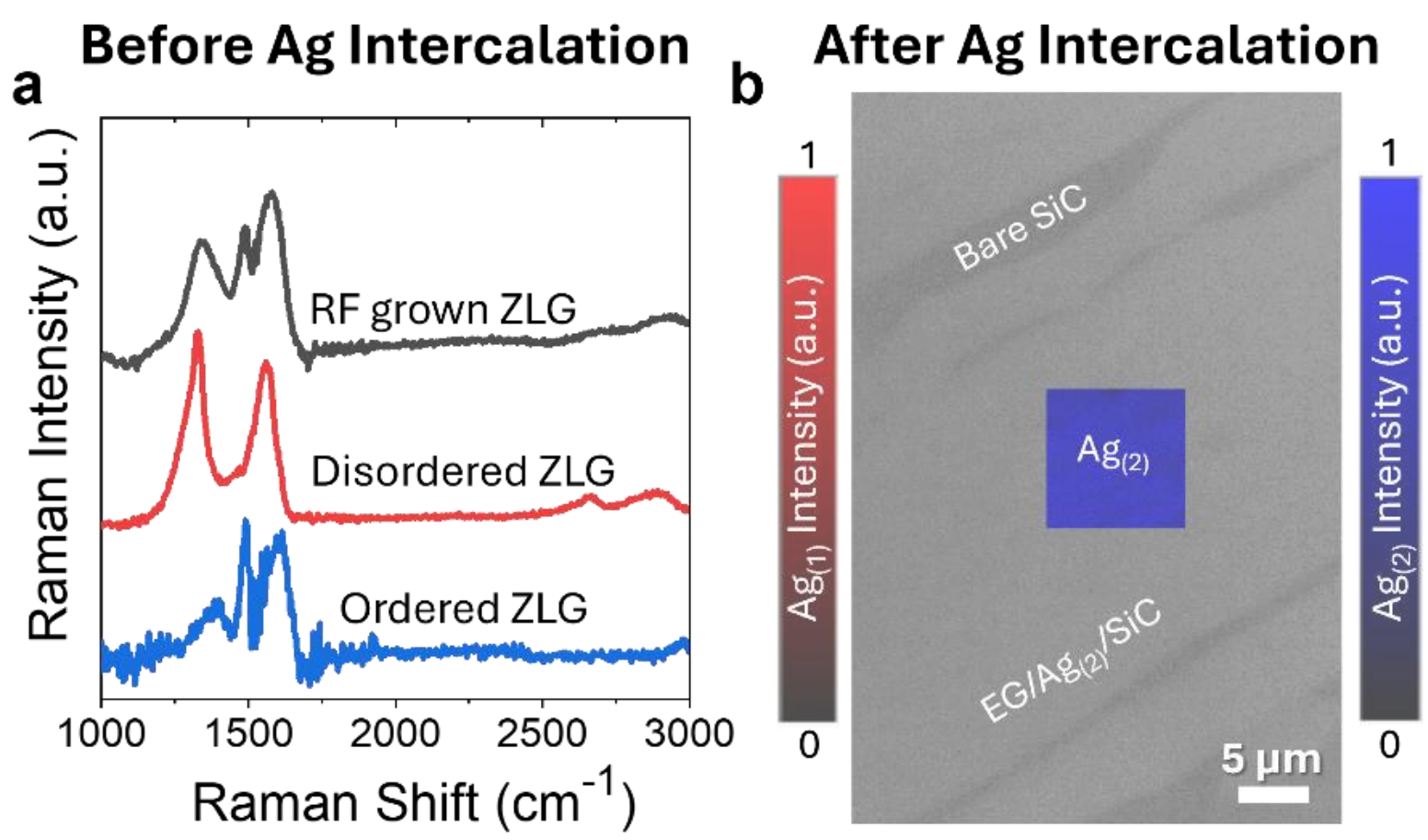

Figure S4. Ag CHet-intercalation on RF-furnace grown ZLG. (a) Representative background-subtracted Raman spectra for RF furnace-grown ZLG from MPI and ordered/disordered ZLG samples from PSU. **(b)** Optical micrograph of Ag CHet-intercalated sample (60 min) on RF grown ZLG, with inset showing a 10 × 10 µm² Raman map of the ULF region. Raman intensities of $Ag_{(1)}$ modes (16, 18.5, and 25 cm^{-1}) are shown in red, and $Ag_{(2)}$ modes (69, 81, and 91 cm^{-1}) in blue. Color bars represent relative intensities of each phase.

XPS Characterization of Silver intercalated on Helium Plasma Treated Graphene

This note discusses the evolution of silver intercalation in helium (He) plasma treated epitaxial graphene (EG) using high-resolution core level X-ray photoelectron spectroscopy (XPS). The intercalation process is tracked through changes in the C 1s, Si 2p, and Ag 3d spectra. Before intercalation, the C 1s spectrum (**Figure S5a**) reveals peaks for the SiC substrate (283.8 eV), the sp^2 graphene signal (284.5 eV), buffer layer components S1 (284.8 eV) and S2, and oxygen-functionalized carbon species, including C–O (285.8 eV) and C=O (288.3 eV). The corresponding Si 2p spectrum (**Figure S5b**) displays the SiC bulk (101.4 eV) and surface (101.9 eV) peaks, along with a SiO_x shoulder near 102.5 eV.

After Ag intercalation, the C 1s spectrum (**Figure S5d**) exhibits a clear shift in the SiC peak to lower binding energy (282.5 eV), consistent with downward band bending. Simultaneously, the buffer layer peaks (S1, S2) are significantly suppressed, and a strong sp^2 graphene peak re-emerges at 284.5 eV—indicative of buffer decoupling.[1,2] The disappearance of the oxygen-functionalized carbon peaks signals effective graphene healing during intercalation.[1] Si 2p spectra (**Figure S5e**) further confirm band bending, as the Ag–SiC peak shifts to ~100.2 eV, and a SiC′ peak at 100.9 eV reflects partially unintercalated regions. The Ag 3d core level (**Figure S5c**) presents peaks at 368.4 eV (Ag-Ag binding, Ag^0) and 368.8 eV (Ag–Si bonding), confirming presence of intercalated silver. The Ag MNN Auger spectrum (**Figure S5f**) yields an Auger parameter exceeding 725 eV, which also verifies the presence of silver in the sample.

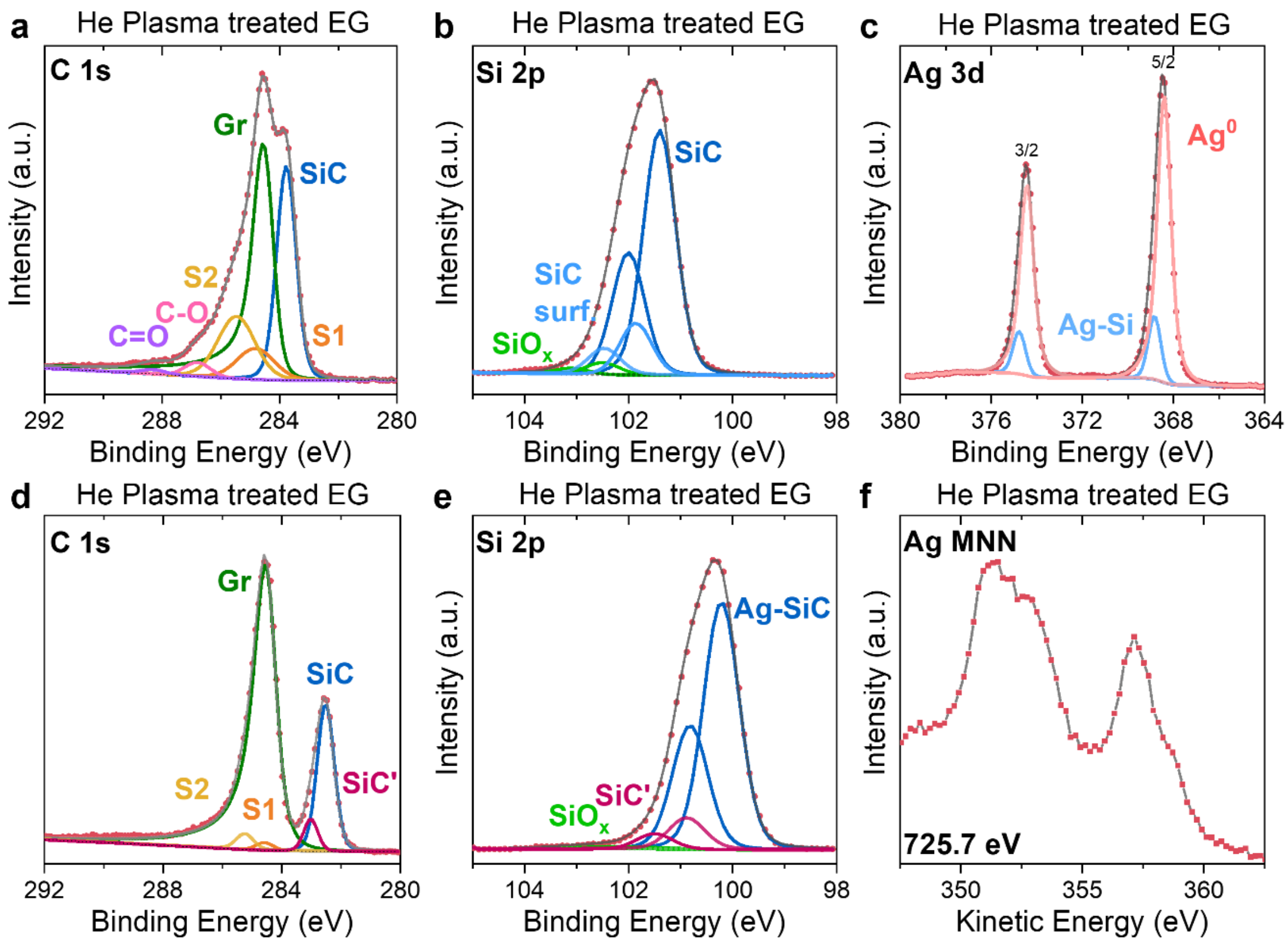

Figure S5. XPS characterization of Ag intercalation on helium plasma treated EG. High-resolution **(a, d)** C 1s and **(b, e)** Si 2p XPS spectrum for 3-minute helium plasma treated EG, before and after Ag intercalation, respectively. **(c)** Ag 3d XPS and **(f)** MNN Auger spectrum for the same sample after intercalation, confirming the presence of Ag and successful interface bonding with SiC.

Metastability of the Ag(1) phase

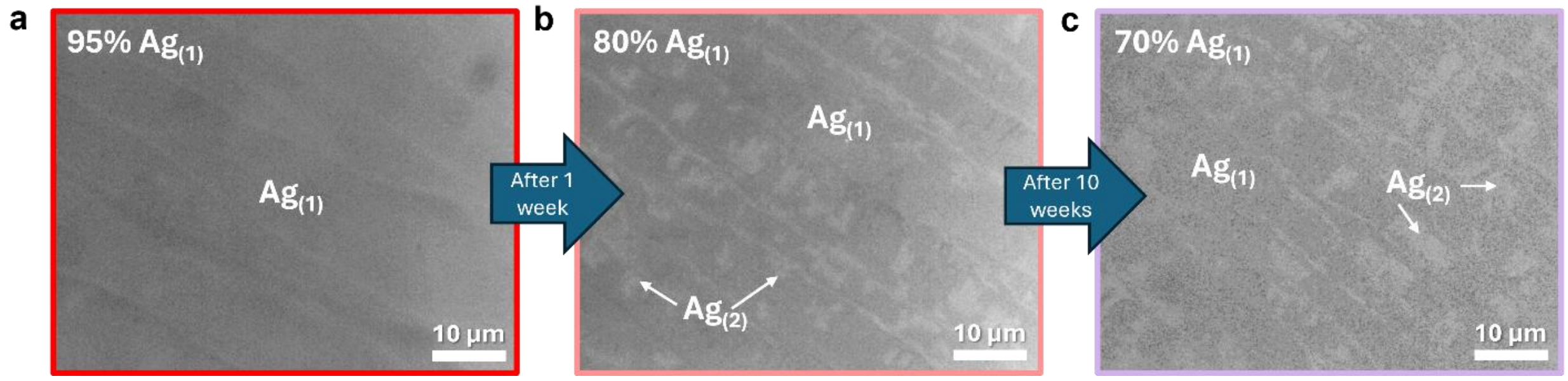

Figure S6. Time-dependent conversion of $Ag_{(1)}$ to $Ag_{(2)}$. Greyscale optical micrographs of a 2D silver sample grown on 3-minute Helium plasma treated EG **(a)** immediately after growth, **(b)** after 1 week, and **(c)** after 10 weeks of storage. Over time, dark-contrast $Ag_{(1)}$ regions progressively convert into bright-contrast $Ag_{(2)}$ domains, with a rapid initial transformation that slows and saturates at ~30% conversion. This behavior experimentally confirms the higher thermodynamic stability of $Ag_{(2)}$ compared to $Ag_{(1)}$. Samples were stored in a nitrogen-filled drawer at room temperature and briefly exposed to ambient conditions during each characterization.

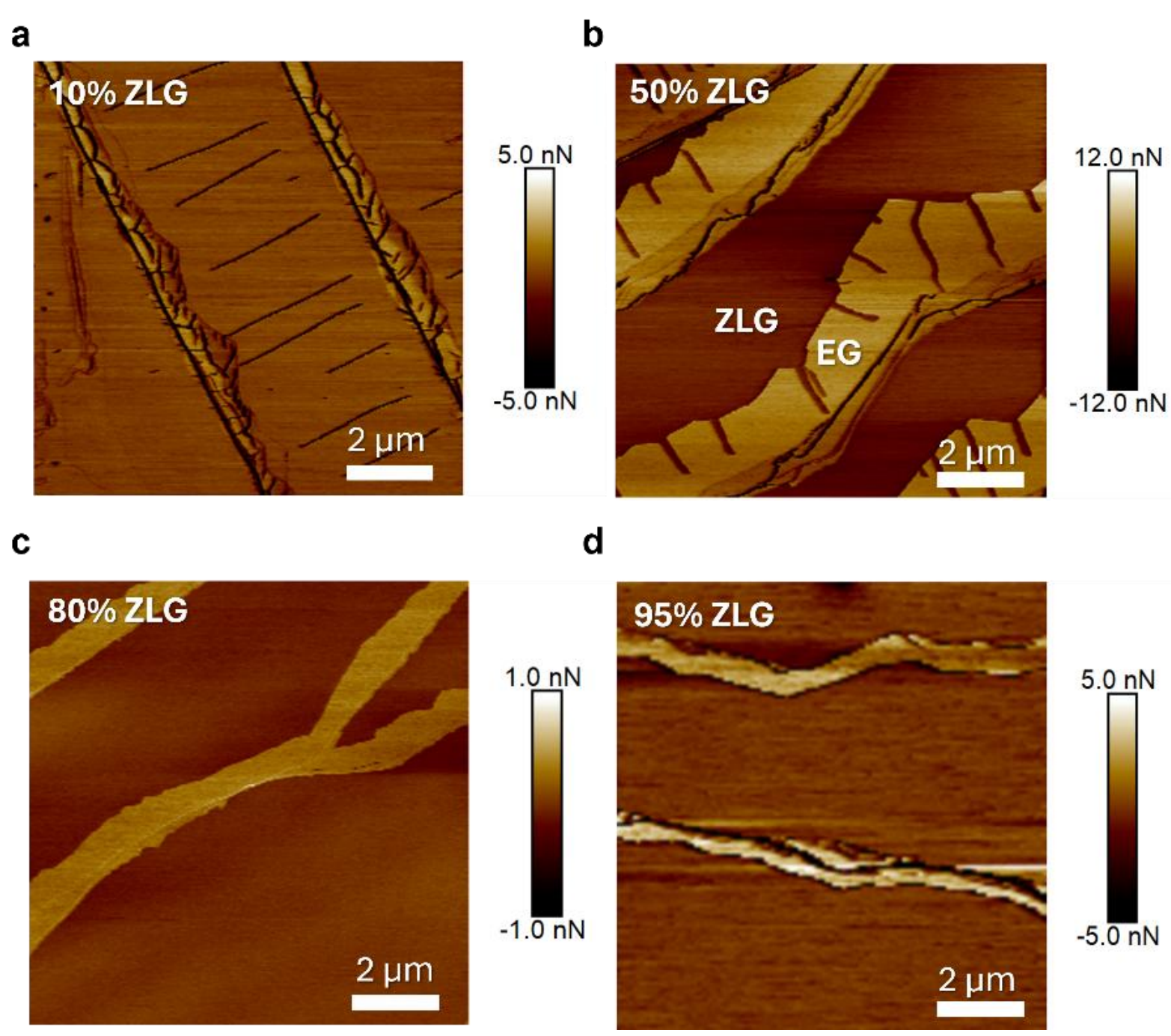

Figure S7. Atomic Force Microscopy (AFM) characterization of ZLG coverage. AFM Adhesion maps for **(a)** 10%, **(b)** 50%, **(c)** 80%, and **(d)** 95% exposed zero layer graphene (ZLG) illustrate a reliable method for quantifying ZLG coverage. Regions with lower adhesion (darker contrast) correspond to ZLG, while higher adhesion (brighter contrast) indicates overgrown epitaxial graphene (EG). These adhesion contrasts provide a facile and spatially resolved approach to estimate ZLG percentage across the surface.

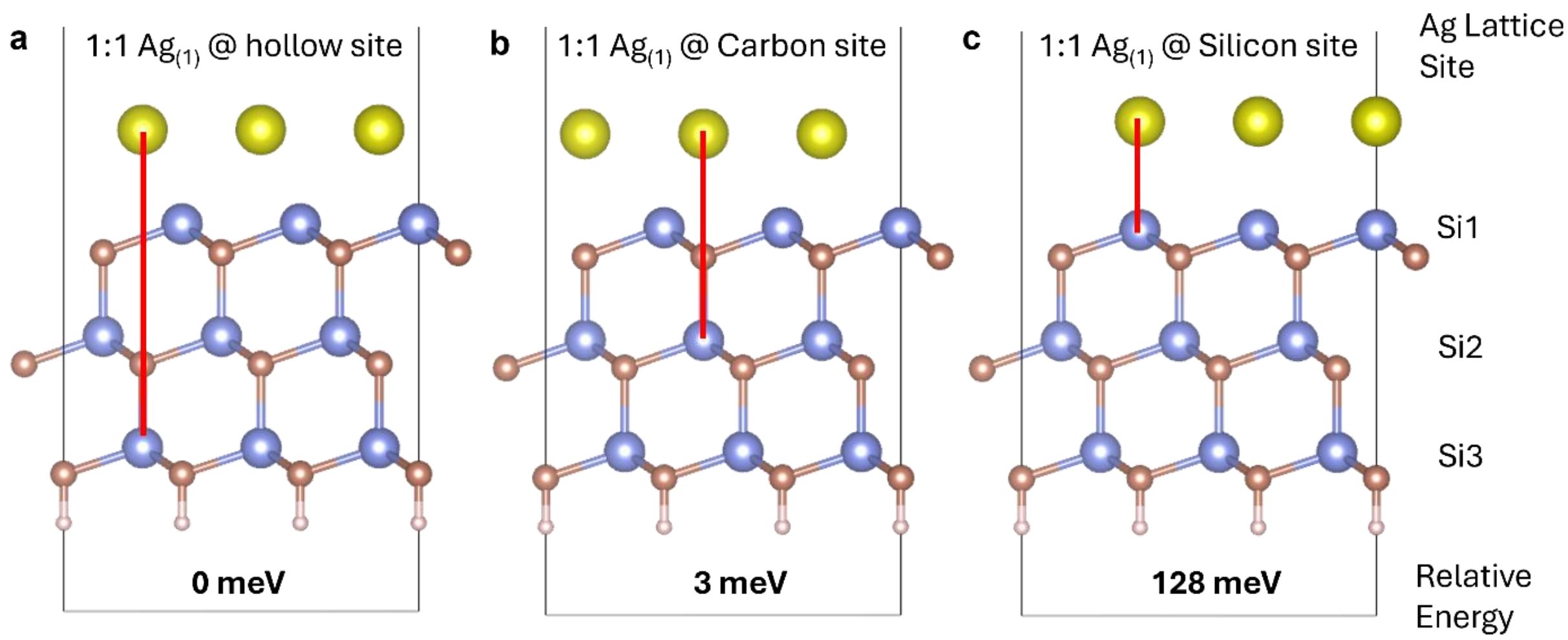

Figure S8. Stable lattice registries for $Ag_{(1)}$ over SiC. Side-view structural models of the three stable registries of the $Ag_{(1)}$ phase over the SiC substrate: $Ag_{(1)}$(hollow), $Ag_{(1)}$(carbon), and $Ag_{(1)}$(silicon). Relative total energies are reported with respect to the hollow-site configuration, which is the lowest-energy and therefore most thermodynamically favorable registry. These configurations illustrate the registry-dependent bonding behavior that contributes to the coexistence and optoelectronic properties of the $Ag_{(1)}$ phase.

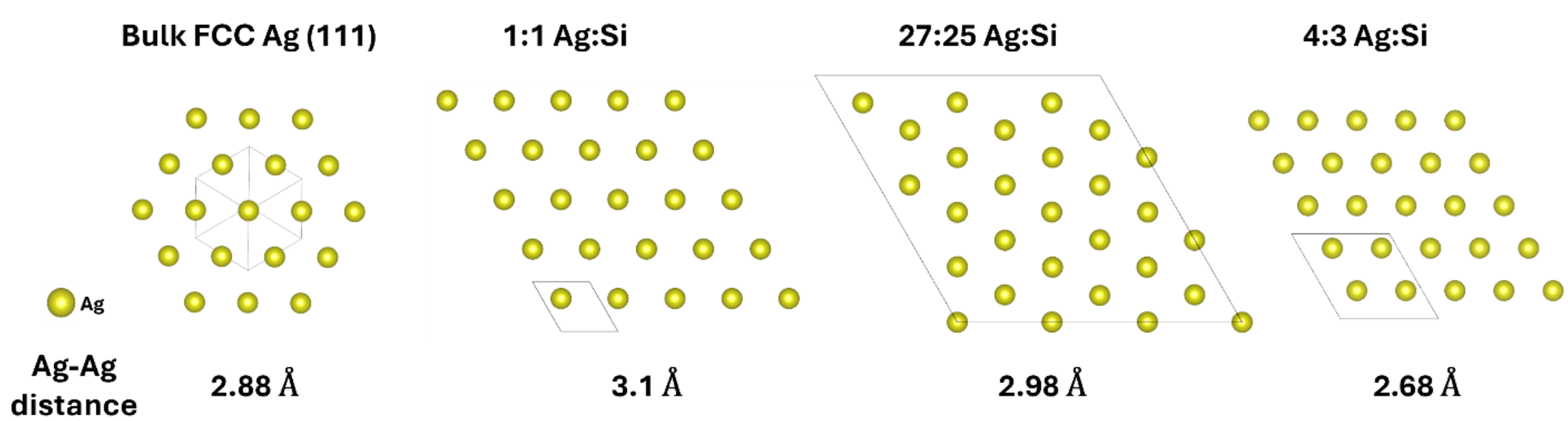

Figure S9. Comparison of Ag-Ag interatomic spacing in various packing densities of Ag. Top-view structural models showing the atomic arrangement of monolayer Ag in three different commensurate phases with the SiC substrate— 1:1 ($Ag_{(1)}$), 27:25 ($Ag_{(2)}$), and (c) 4:3 Ag:Si configurations. These are compared with the bulk Ag FCC (111) plane. The Ag–Ag interatomic distances vary between these configurations, illustrating how 2D confinement and registry with the underlying substrate modulate the atomic packing density of the intercalated silver. Respective unit cells are shown in light grey.

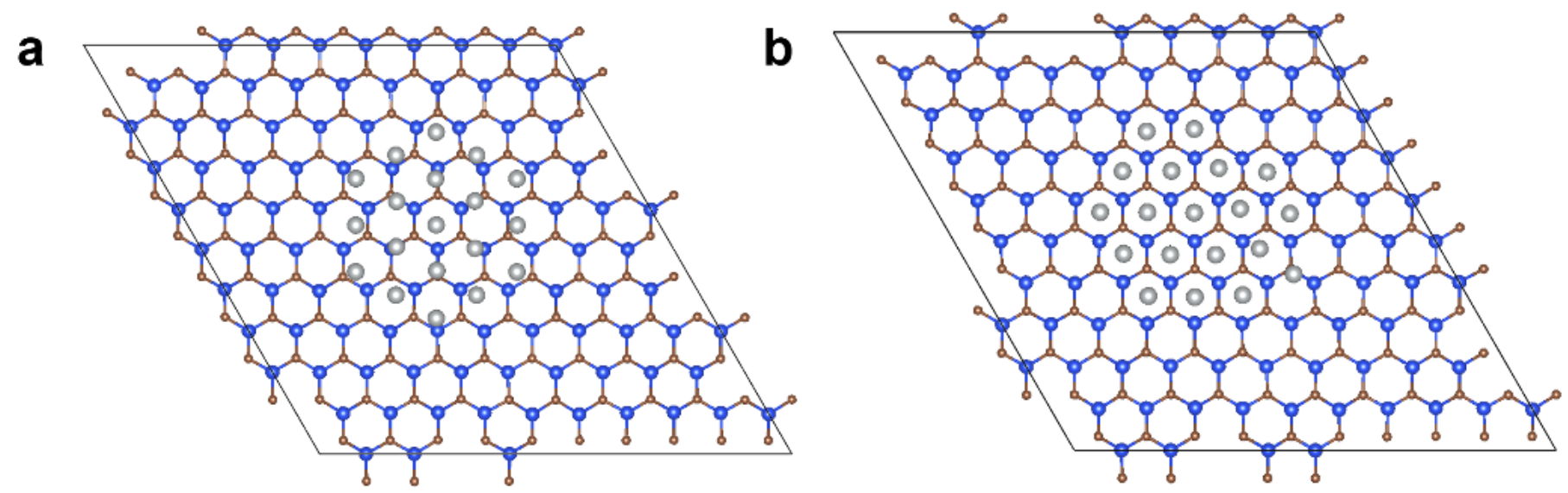

Figure S10. Silver cluster calculation. **(a)** Initial configuration of a finite $Ag_{(2)}$ phase cluster with a diameter of ~12 Å placed on SiC, with only the topmost atomic layer of SiC visualized. **(b)** Upon structural relaxation, the Ag atoms reorganize into $Ag_{(1)}$(hollow) registry, indicating that small $Ag_{(2)}$ clusters are unstable and tend to relax into the $Ag_{(1)}$ phase. This calculation highlights the role of size and registry in phase stabilization of 2D Ag.

Low-energy electron diffraction (LEED) pattern analysis

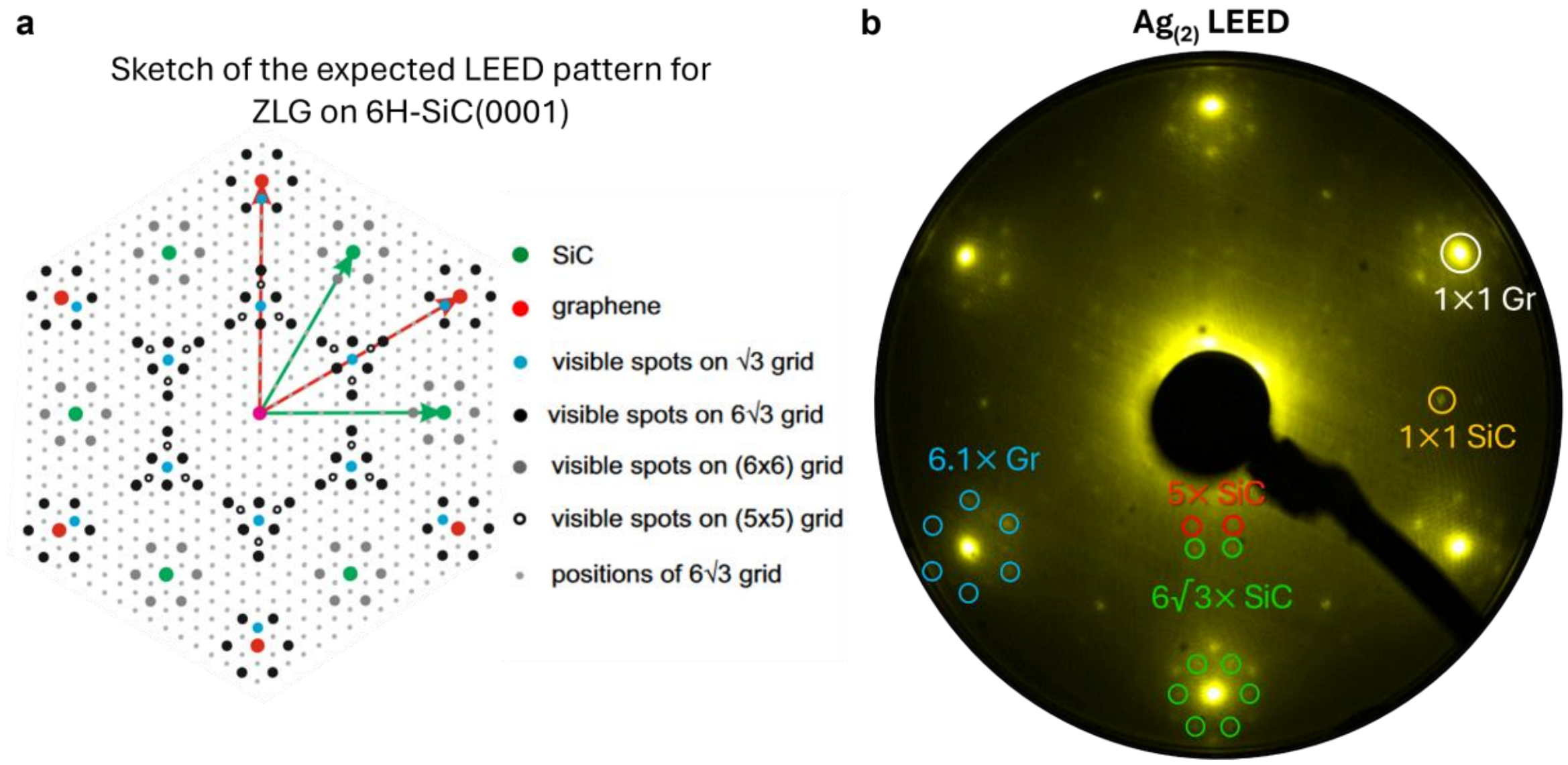

Figure S11. LEED pattern analysis for $Ag_{(2)}$. (a) Simulated LEED pattern for ZLG on 6H-SiC(0001) showing characteristic diffraction spots associated with the SiC substrate and the (6√3 × 6√3)R30° buffer layer reconstruction. (b) Experimentally acquired LEED pattern for a predominant $Ag_{(2)}$ sample, where additional satellite spots emerge beyond the standard ZLG pattern. These $Ag_{(2)}$-specific spots can be attributed to the (27:25) commensurate superstructure, confirming long-range ordering in the intercalated Ag layer.

Table S1: Lattice constants comparison for LEED

**Graphene**	2.46	6.1× = 15		13× = 32.0
**Silver ($Ag_{(2)}$)**	~2.98	5×(3) = 15	**3√3×**(2.98) = 15.5	11×(2.93) = 32.2
**SiC**	3.10		**5×** = 15.5	6√3× = 32.2

Table S1. Lattice constants comparison for LEED. All values are in angstroms (Å). By analyzing lattice constant mismatches, we determine the in-plane lattice parameter of the $Ag_{(2)}$ phase to be approximately 2.98 Å. This leads to a commensurate 27:25 matching with the SiC substrate—i.e., a (3√3 × 3√3)R30° $Ag_{(2)}$ lattice on top of a 5 × 5 SiC supercell.

Energy-dispersive X-Ray Spectroscopy (EDS) and Transmission Electron Microscopy (TEM) characterization

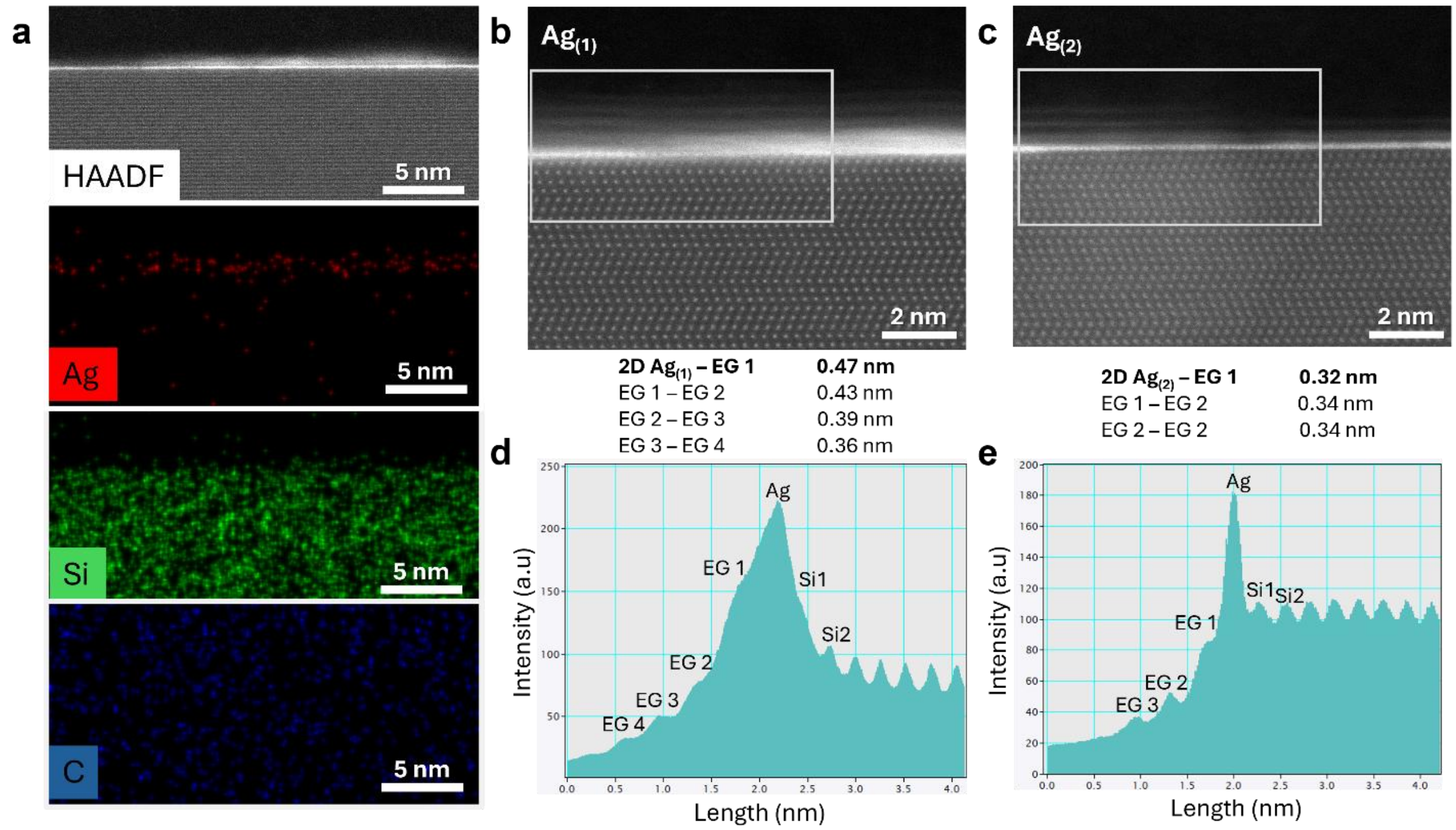

Figure S12. EDS and additional TEM characterization of intercalated 2D Ag. **(a)** High-angle annular dark-field scanning transmission electron microscopy (HAADF-STEM) image and corresponding energy-dispersive X-ray spectroscopy (EDS) elemental maps for 2D $Ag_{(2)}$, highlighting the spatial distribution of Ag, Si and C within the cross-section. The results confirm successful silver intercalation beneath epitaxial graphene, forming a predominantly monolayer Ag structure capped by a single graphene layer. Excess carbon at the top comes from the protective amorphous carbon cap on EG/Ag/SiC. **(b–c)** Cross-sectional HAADF-STEM images of monolayer $Ag_{(1)}$ and $Ag_{(2)}$, respectively, acquired near SiC step edges. **(d–e)** Intensity profiles extracted from the white rectangles in panels **(b)** and **(c)**, respectively, reveal a larger vertical separation between Ag and EG in $Ag_{(1)}$ (0.47 nm) compared to $Ag_{(2)}$ (0.32 nm). Note: Cross-sectional TEM measurements were performed at step edges containing multilayer graphene for better contrast, and may not fully represent terrace-level Ag–EG separation.

Semiconducting Behavior of Graphene-capped $Ag_{(2)}$

The electronic behavior of confined 2D Ag is governed by two related effects: the formation of an underlying gap in the Ag-derived band structure and the position of the Fermi level within that structure. For the commensurate $Ag_{(1)}$ phase, previous calculations established that bonding to the Si-terminated SiC substrate breaks the symmetries of the corresponding freestanding triangular Ag lattice and enables hybridization and anticrossing among Ag-derived (s), (p_z), and (d_{z^2}) bands.[7] Together with the appropriate electron filling from the Ag (5s) states and surface-Si dangling bonds, these local gaps combine to produce a global bandgap. This established $Ag_{(1)}$/SiC mechanism provides a framework for understanding the underlying gapped band manifold in $Ag_{(2)}$, although its different packing density substantially modifies the band filling.

For the ideal $Ag_{(2)}$ (27:25) band structure calculated without graphene, the two additional Ag atoms per supercell introduce excess electrons that shift the Fermi level into the conduction band, producing a self-doped metallic state despite the presence of an underlying gap in the band manifold (**Figure S13).** In contrast, ARPES measurements of the experimentally realized graphene-capped $Ag_{(2)}$ heterostructure show no Ag-derived bands crossing the Fermi level (**Figure 3f, g**), while scanning tunneling spectroscopy reveals a finite-energy conduction-band onset (**Figure S16**), consistent with semiconducting behavior.

This difference is attributed primarily to charge redistribution within the graphene/$Ag_{(2)}$/SiC heterostructure. The vacuum-level alignment shown in **Figure S13** favors electron transfer from $Ag_{(2)}$/SiC to the graphene overlayer. This interpretation is consistent with the larger graphene Fermi pockets and stronger n-type doping measured above $Ag_{(2)}$ compared with $Ag_{(1)}$ (**Figure 3h, i**). Electron transfer to graphene therefore partially compensates the excess charge associated with the denser $Ag_{(2)}$ structure and shifts the Fermi level toward the underlying gap.

Because graphene has a relatively low density of states near its Dirac point, the extent to which graphene alone compensates the $Ag_{(2)}$ self-doping remains uncertain. Native point defects observed by STM (**Figure 3e**), potentially including Ag vacancies or exchanged Si species, may provide additional charge-compensation or trapping pathways, although their precise contribution requires further investigation. Thus, the SiC substrate establishes the underlying gapped band structure, while the $Ag_{(2)}$ packing density, graphene-mediated charge transfer, and possible defect-

assisted compensation determine the experimentally observed Fermi-level position and semiconducting behavior.

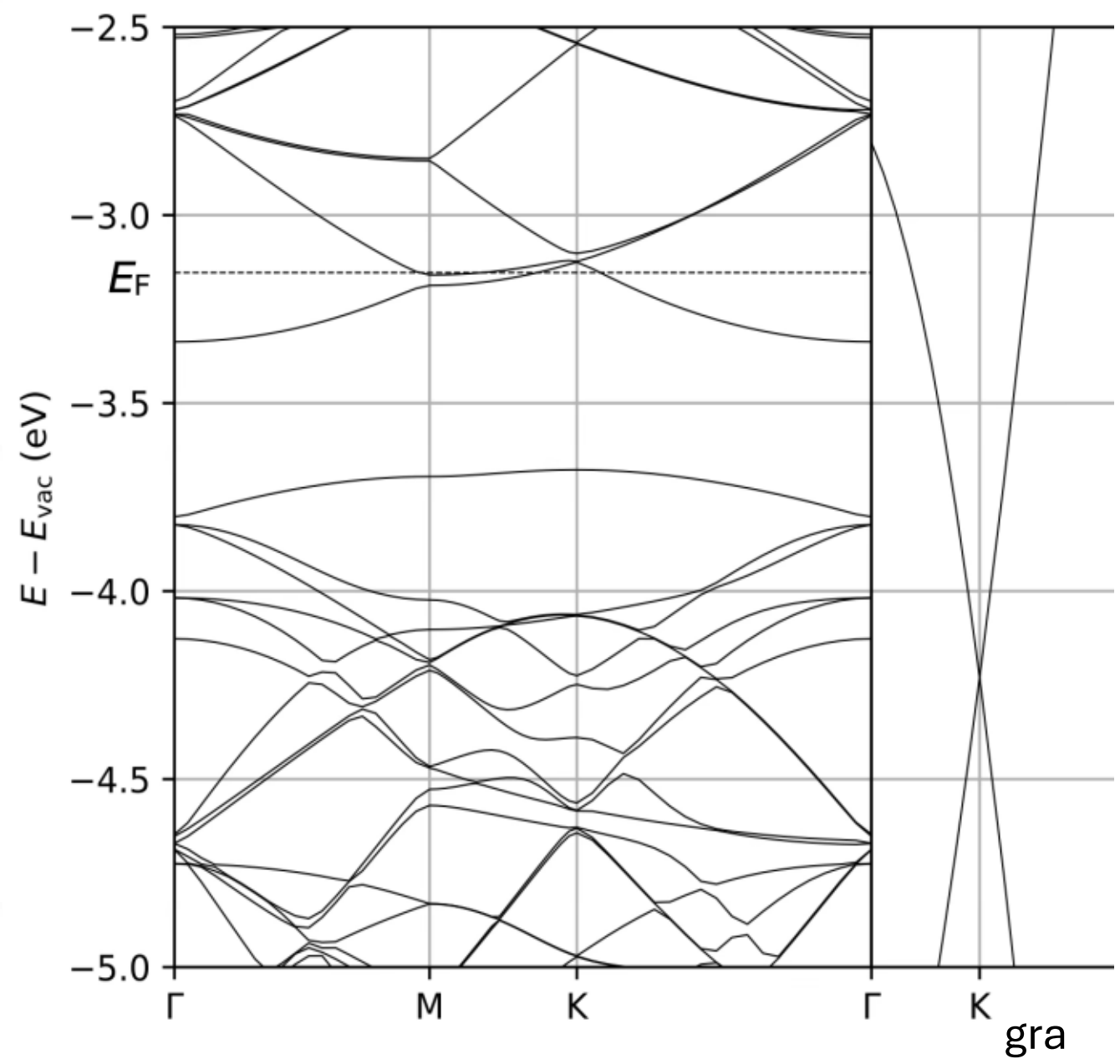

Figure S13. Calculated band structure for $Ag_{(2)}$ and comparison with freestanding graphene. Left: Electronic band structure of the $Ag_{(2)}$ (27:25) phase, referenced to the vacuum level. The Fermi level (dashed line) lies within the conduction band due to self-doping introduced by the two excess Ag atoms per supercell, indicating intrinsic metallicity in the absence of graphene. Right: Dirac cone band structure of freestanding monolayer graphene. The energy offset highlights that the Fermi level of $Ag_{(2)}$ resides above that of graphene, enabling charge transfer when the two systems are brought into proximity. This interaction results in partial electron depletion from the Ag–SiC system into the graphene cap.

Correlative Characterization of 2D Ag phases through Raman, SEM, AFM and AES mapping

Correlative multimodal imaging techniques enable precise identification and differentiation of $Ag_{(1)}$ and $Ag_{(2)}$ phases in 2D silver heterostructures. Prior studies have established that $Ag_{(1)}$ and $Ag_{(2)}$ exhibit distinct low-frequency Raman responses and different Auger electron emission intensities due to their differing silver concentrations.[8] Additionally, theoretical and experimental works have proposed that 2D Ag can form various commensurate structures, including 1:1 and 4:3 Ag:Si phases, with 1-4 layer Ag coverage.[9,10] To confirm phase assignment, thickness uniformity, and packing density in our intercalated samples, we employ a combination of Raman spectroscopy, secondary electron SEM, AFM, and AES mapping.

We perform all characterizations on a single terrace of a large-area, phase-segregated sample with optically visible $Ag_{(1)}$ and $Ag_{(2)}$ domains (**Figure S14a**), thus minimizing contrast artifacts from graphene thickness variation. Raman mapping in the ultra-low frequency (ULF) range confirms the presence of intercalated silver across the terrace, with red areas corresponding to $Ag_{(1)}$ and blue areas to $Ag_{(2)}$. The 2D peak width map clearly reveals uniform bilayer graphene on the terrace and thicker graphene on the edges.[11] After background subtraction, the graphene G and 2D peak positions shift differently depending on the underlying Ag phase—$Ag_{(2)}$ shows a G-peak blueshift and 2D-peak redshift compared to $Ag_{(1)}$, indicating variations in local strain and doping.[12] Decomposition of strain and doping contributions reveals that graphene above $Ag_{(2)}$ experiences greater tensile strain (~0.14%) and higher n-type doping ($\sim 3.2\times10^{13}$ cm^{-2}) than graphene above $Ag_{(1)}$ (~0.03%, $\sim 2.0\times10^{13}$ cm^{-2}), supporting stronger interaction and electron transfer at the $Ag_{(2)}$ interface—consistent with calculations in **Figure S13**, and experimental ARPES (**Figure S17**) and TEM (**Figure S12**) measurements.

In secondary electron SEM images acquired on the same terrace, $Ag_{(2)}$ domains appear darker than $Ag_{(1)}$ (**Figure S14c**). This difference arises from the lower secondary electron emission yield of more n-doped graphene over $Ag_{(2)}$ than $Ag_{(1)}$, which reduces surface band bending and emission efficiency.[13] AFM topography and adhesion mapping further support these findings. Adhesion contrast tracks well with SEM and Raman features, but height maps reveal no measurable step between $Ag_{(1)}$ and $Ag_{(2)}$, confirming that both phases exhibit similar layer thicknesses. If a layer

number difference existed, a ~0.3 nm height step—typical for additional silver layers—would have appeared.

To verify the difference in silver packing density between the two phases, we perform Auger Electron Spectroscopy (AES) mapping in the same region (**Figure S15**). AES intensity maps show $Ag_{(2)}$ domains as brighter than $Ag_{(1)}$, consistent with higher silver content. Since both regions have the same layer thickness and identical bilayer graphene capping, the contrast in AES must arise from differences in Ag atom packing. ImageJ analysis of regions of interest on the terrace yields an $Ag_{(2)}/Ag_{(1)}$ AES intensity ratio of 1.09 ± 0.01, aligning closely with the expected 27:25 Ag:Si commensuration ratio (1.08). This observation confirms that $Ag_{(2)}$ adopts a denser packing structure than $Ag_{(1)}$ and further supports our assignment of the $Ag_{(2)}$ phase as the 27:25 commensurate structure on SiC.

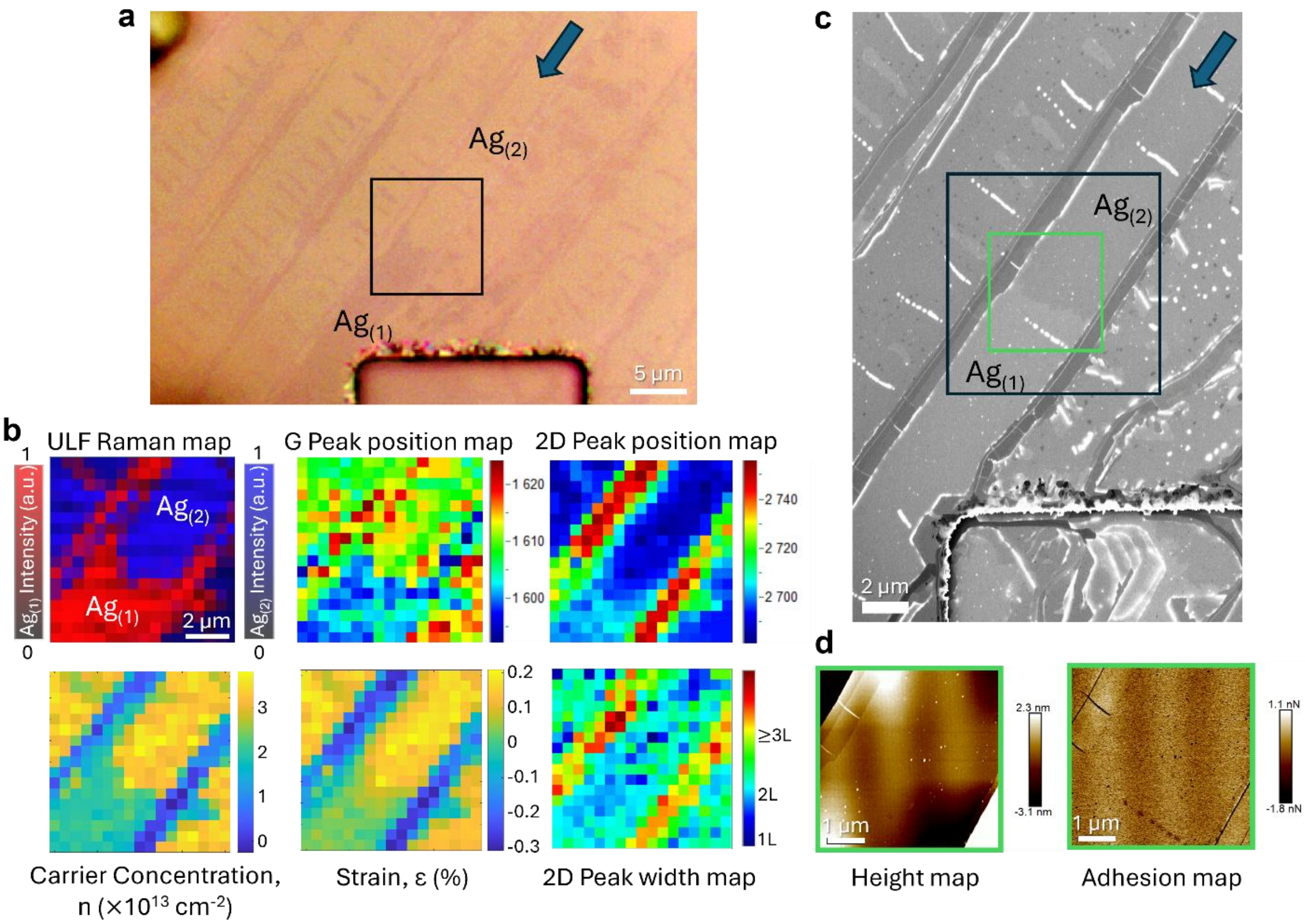

Figure S14. Correlative Optical, Raman, SEM and AFM characterization of 2D Ag phases. **(a)** Optical micrograph of a mixed-phase 2D Ag sample, showing $Ag_{(1)}$ (dark) and $Ag_{(2)}$ (bright) domains on a single terrace (marked with a blue arrow). A lithographic marker at the bottom aids spatial correlation. **(b)** Raman maps from the 10 × 10 μm² region (black square in **(a)**), showing ultra-low frequency (ULF) Raman intensity to distinguish $Ag_{(1)}$ and $Ag_{(2)}$, G and 2D peak positions, 2D peak width (layer number), and extracted strain and carrier concentration maps based on vector decomposition of the G/2D positions. **(c)** Secondary electron SEM image of the same region, showing contrast inversion between phases ($Ag_{(1)}$ – bright, $Ag_{(2)}$ – dark). The blue arrow and black square denote the same terrace and Raman-mapped region, respectively. **(d)** AFM topography and adhesion maps of a 5 × 5 μm² region (green square in **(c)**), across the $Ag_{(1)}/Ag_{(2)}$ boundary, showing no height difference but distinct adhesion contrast correlated with surface features.

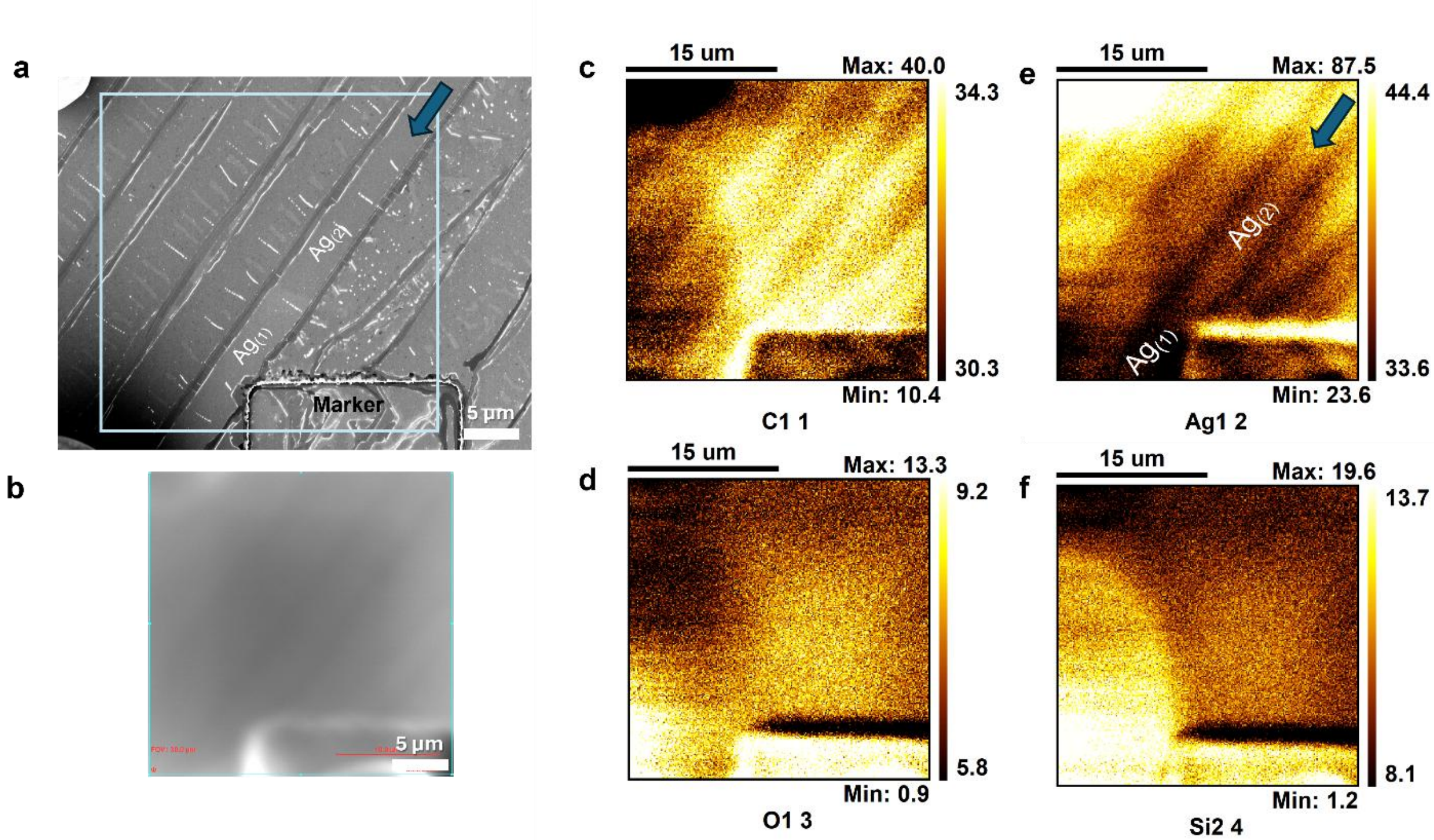

Figure S15. Correlative Auger Electron Spectroscopy (AES) and SEM characterization of 2D Ag phases. **(a)** Secondary electron SEM micrograph of the same sample in **Figure S14a**, highlighting $Ag_{(1)}$ and $Ag_{(2)}$ regions on a single terrace (blue arrow). The lithographic marker and a silver particle (white spot, top-left) serve as identifiers. **(b)** SEM image from the AES instrument showing the same area. **(c–f)** AES elemental maps from the 30 × 30 μm² region marked with a light blue square in **(a)**, showing distributions of **(c)** carbon, **(d)** oxygen, **(e)** silver, and **(f)** silicon. The blue arrow in **(e)** marks the same terrace as in **Figure S14**. $Ag_{(1)}$ and $Ag_{(2)}$ regions are labeled.

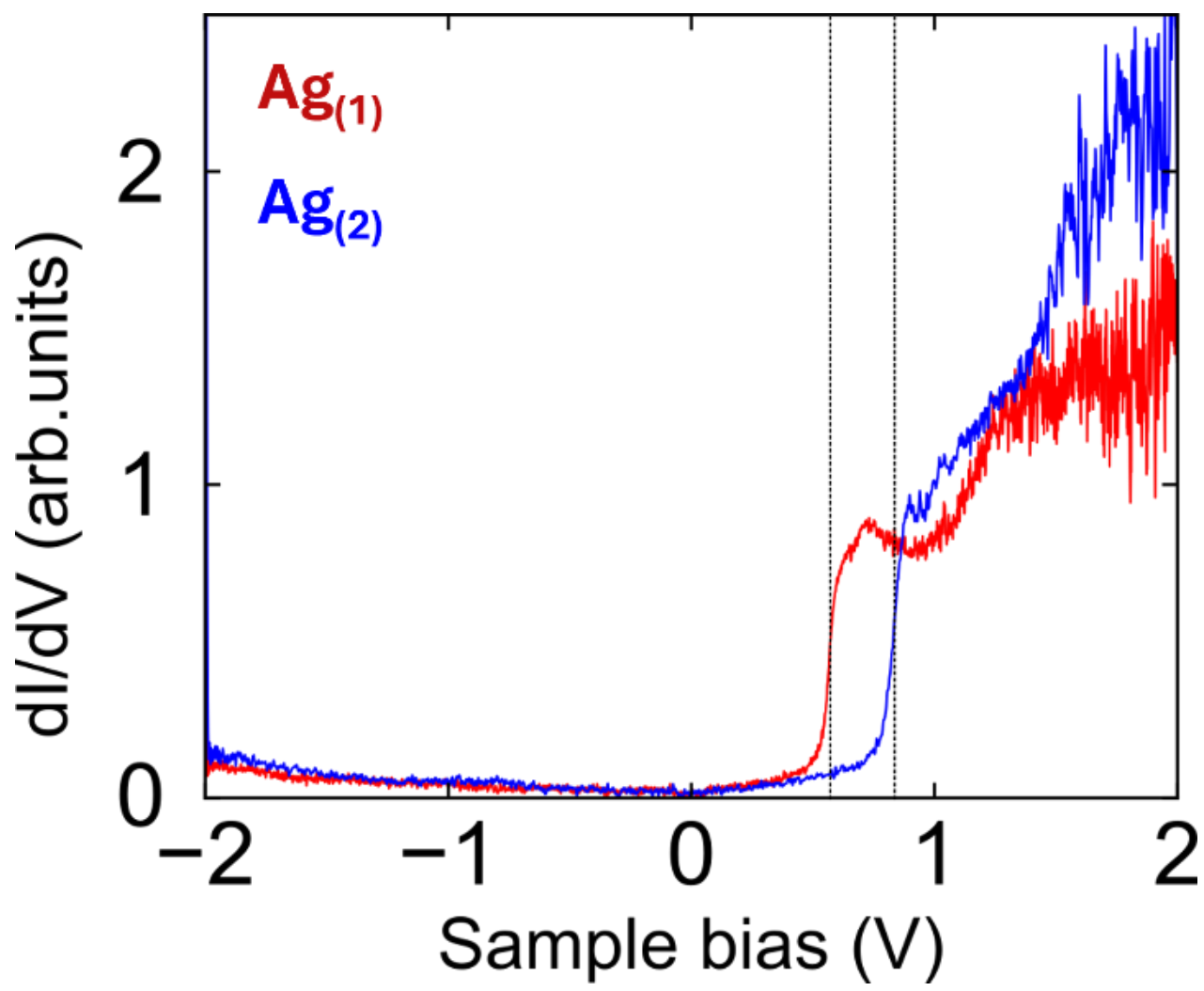

Figure S16. Scanning Tunneling Spectroscopy (STS) comparison of 2D Ag phases. Scanning tunneling spectroscopy (dI/dV) spectra acquired from $Ag_{(1)}$ and $Ag_{(2)}$ regions shown in **Figure 3(e)** reveal distinct phase-dependent electronic signatures.

Additional ARPES data and analysis

Figure S17a and **S17b** present the electronic band structures of the $Ag_{(1)}$ and $Ag_{(2)}$-intercalated samples, respectively, along the $\overline{M_{SiC}\Gamma K_{SiC}}$ direction, measured with lab-based 21.22 eV photons. The Brillouin zone (BZ) SiC, which coincides with $Ag_{(1)}$ BZ, is rotated by 30° relative to the graphene BZ as shown in **Figure S17c**. For both $Ag_{(1)}$ and $Ag_{(2)}$ ARPES data, the most intense features are the graphene π-band around 3.0 eV binding energy (BE) at $\overline{M}_{gr}$ ($k_{\|} = 1.474$ Å^{-1}) and the SiC valence band below 2.0 eV BE at Γ. All other observed bands originate from the intercalated Ag layer.[6,14,15] For $Ag_{(1)}$, the valence band maximum (VBM) is located at the $\overline{K}_{SiC}$ point at approximately 0.4 eV BE, and the saddle point at $\overline{M}_{SiC}$ lies near 1.2 eV BE (both are shown by the brown arrows in **Figure S17a**). In the $Ag_{(2)}$ sample, the VBM is too weak to clearly identify the band maximum, but the saddle point (shown by the blue arrow in **Figure S17b**) is shifted by approximately 0.2 eV toward higher binding energies.

Figure S17(d) presents the high-resolution Dirac cone for the $Ag_{(2)}$-intercalated sample, measured with 180 eV photon energy. Two distinct Dirac cones are clearly resolved, with Dirac points at approximately 0.3 eV (E'_{D2}) and 0.8 eV (E_{D2}). The momentum distribution curves (MDCs) (red dots) taken at the Fermi level (E_F) of **Figure 3h** and **Figure S17(d)** respectively for $Ag_{(1)}$ (**Figure S17 e(i)**) and $Ag_{(2)}$ (**Figure S17 e(ii)**) clearly reveal the opposite intensity patterns for QFMLG and QFBLG Dirac cones in these samples. From the fitted data (black line in the figure), the peak separation between the two branches of the Dirac cone corresponding to both QFMLG and QFBLG layer is more than 0.01 Å^{-1} larger for Ag_2. In other words, the Fermi pockets of both the QFMLG and QFBLG π^* bands are larger in the Ag_2 sample. It is important to note that the separation between the Ag_2-QFMLG π^*band peaks in **Figure S17 e(ii)** correspond to k_F= 0.274 Å^{-1}, which was used to calculate the charge carrier density in the main text.

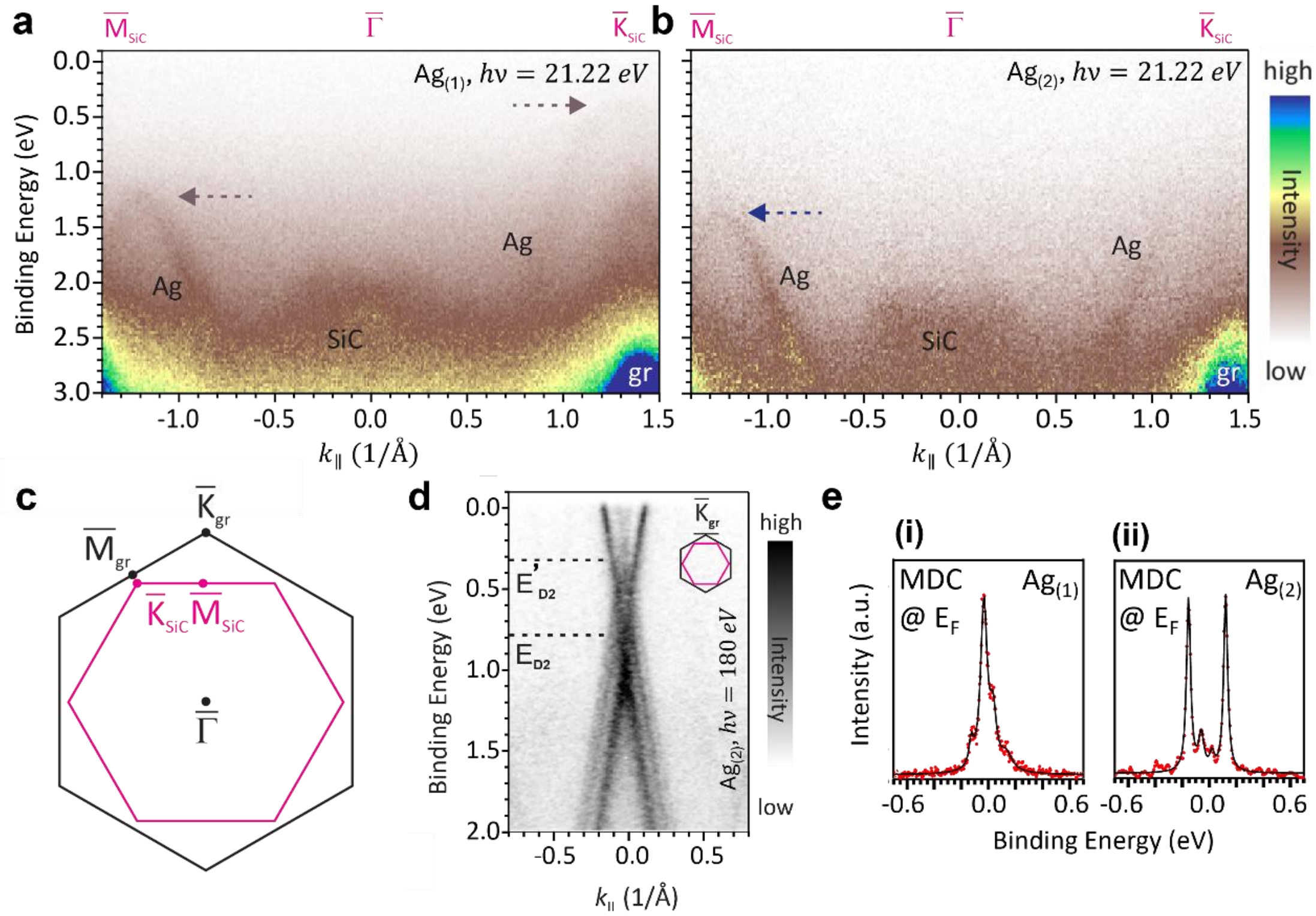

Figure S17. Electronic band structure of $Ag_{(1)}$ and $Ag_{(2)}$ intercalated graphene. Lab-based He Iα measurements of Ag band dispersion along $\overline{M_{SiC}\Gamma K_{SiC}}$ for **(a)** $Ag_{(1)}$ and **(b)** $Ag_{(2)}$. Brown arrows in **(a)** and blue arrows in **(b)** mark characteristic bands of $Ag_{(1)}$ and $Ag_{(2)}$, respectively. **(c)** Brillouin zone (BZ) of Ag/SiC (magenta), rotated and enclosed within the graphene BZ (black). **(d)** High-resolution Dirac cone of $Ag_{(2)}$–QFMLG (180 eV photons), with the corresponding path shown in the inset. **(e)** Momentum distribution curves (MDCs) at the Fermi level (E_F) for $Ag_{(1)}$ (**Figure 3h**) and $Ag_{(2)}$ **(Figure 3i)** are displayed; red dots indicate raw data, and black lines are fits.

Determination of the dielectric function from spectroscopic imaging ellipsometry (SIE)

Figure S18a and **b** show SIE Ψ-maps taken at 450 nm (~2.76 eV) on the $Ag_{(1)}$ (sample A) and $Ag_{(2)}$ (sample B) dominant samples, respectively. Several regions of interest (ROIs) of homogeneous response were chosen from both samples (outlined rectangles, **Figure S18a** and **b**. The $Ag_{(1)}$ regions on the $Ag_{(2)}$ dominant sample were deemed to small and therefore noisy for further investigation. The hyperspectral maps are manually corrected for small sample movements between measurements taken with different wavelengths in order analyze the response from the identical ROI on the samples for the whole spectral range. . Averaging the response over each ROI results in a set of ellipsometric spectra Ψ(λ), Δ(λ).

In ellipsometry, the change of the polarization state of light being reflected from the surface of a thin multilayer system is measured. Here we use the Jones matrix representation allowing to express the change ρ in the polarization by the ellipsometric angles Ψ(λ), Δ(λ) in the following way:

$$\rho = \frac{r_p}{r_s} = \frac{|r_p|}{|r_s|} e^{i(\delta_{rp} - \delta_{rs})} = \tan \Psi e^{i\Delta} \ (2)$$

Hereby, r_p and r_s are the complex amplitude reflection coefficients for p- and s-polarized light, which can be rewritten in exponential form with an amplitude $|r_i|$ and a phase shift δ_{ri}. As can be seen, tan(Ψ) can be understood as the amplitude ratio, while Δ is the relative phase shift between p- and s-polarized light reflected from the sample.

The spectra Ψ(λ), Δ(λ) corresponding to sample A can be seen for $Ag_{(1)}$ **Figure S18d, e** and for $Ag_{(2)}$ in **Figure S18f** and **g**. The spectra show a consistent response of the polarization change of the reflected light across the measured area, with the spread on the order of the measurement uncertainty.

To extract the complex dielectric function $\varepsilon(\lambda) = \varepsilon_1(\lambda) + i\varepsilon_2(\lambda)$ for $Ag_{(1)}$ and $Ag_{(2)}$ from the measured spectra Ψ(λ), Δ(λ), a comprehensive optical model is constructed and fit to the spectra using regression analysis. The model consists of a stack of flat layers, see **Figure S18c**, where each layer is fully described by its dielectric function ε(λ) and its thickness t. The top and bottom layers (Air and SiC) are assumed to be semi-infinite. The dielectric function of the 6H-SiC substrate in the

visible is well known and can be approximated in the investigated spectral range by a Sellmeier parametrization.[16]

Thin layers provide additional challenges to ellipsometry. Most importantly, at thicknesses $t \ll 10\,nm$, the dielectric function ϵ and thickness of a layer, can in general not be unambiguously evaluated anymore in what is called the small phase shift condition.[17–20] This is not only a measurement issue, but a physical limitation, as strictly speaking the dielectric function is a 3D bulk quantity and is not defined in the 2D limit. Instead, the measured signal is proportional to the product $(\epsilon - 1)\,t$ and one correctly speaks about an effective dielectric function and an effective thickness. As an immediate consequence, it does not make sense to vary both quantities for a single layer as no additional information can be extracted. The absolute magnitude of the dielectric function can therefore only be understood in conjunction with the reported thickness.

Another issue complicating the modeling of ultrathin films is electronic coupling between layers. In strong cases, such as in transition-metal dichalcogenide (TMD) heterobilayers, the optical response may only be understood as a single layer with an effective dielectric function, rather than as a stack of individual layers[21,22]. As discussed in the main text, the coupling between EG and 2D Ag can be qualitatively understood as electron doping of the graphene overlayer, i.e. a shift of the Fermi levels of two otherwise largely decoupled systems with no orbital hybridization between them[6]. This is analogous to other CHet intercalated metal systems[1,23]. For this reason, we choose to model the system as two decoupled layers, where the doping level is parametrized in the Drude term of the EG layer. The Ag monolayer and the SiC substrate on the other hand form a coupled system, due to the covalent bonding between them with strong contribution from orbitals of the topmost SiC layers expected for the interband transitions seen in $Ag_{(1)}$ and $Ag_{(2)}$.

Optically, this can be thought of as a depth dependent permittivity distribution $\epsilon(\omega, z)$, which one could either try to parameterize outright or use one or several effective medium approximation (EMA) layers[24]. However, in practice, no qualitative difference between the models was found using the EMA approach compared to using two independently parameterized layers. This is most likely due to the limitations of the small phase shift condition discussed above. In DFT calculations, the impact of the hybridization between Ag monolayers and SiC is significantly decaying with thickness and can be accounted for using only a few Si-C bilayers in z-direction, being still in the thin film limit with an effective thickness on the order of a few nanometer. We

therefore retain the simpler layered parameterization while explicitly interpreting the extracted Ag dielectric function as an effective interfacial response. In the following, a more detailed description of the layers is given:

On the top of the structure a thin adsorption layer is included since the measurements are taken in ambient. Taking time-series of spectroscopic ellipsometry measurements of stable CHet 2D metal samples, e.g. fully transitioned $Ag_{(2)}$, we find a shift of the ellipsometric response to lower Δ-values over several months, while the Ψ-spectra stay constant. This behavior is consistent with the accumulation of a transparent layer on top of the sample. The precise nature of this adsorption layer remains unclear. However, it can be satisfactorily modeled using a simple water layer with a variable thickness. For the $Ag_{(1)}$ dominant sample A we find $t_W = 2.39\ nm$, while for the, at point of measurement younger, $Ag_{(2)}$ dominant sample B the fit gives $t_W = 0.75\ nm$.

For graphene the dielectric function varies slightly depending on its preparation, layer number, doping and dielectric environment.[25–27] As discussed, in the case of CHet intercalated 2D Ag, the graphene is n-doped by the underlying metal, compare **Figure 3f**. To account for these additional charge carriers, we base our graphene model on measurements of field-effect doped bilayer graphene,[26] which uses a biaxial model, with an in-plane Lorentz-Drude response, while the out-of-plane component is ε=1. We model the $Ag_{(1)}$ and $Ag_{(2)}$ dominant samples with the assumption of homogeneous quasi-freestanding bilayer and monolayer graphene, respectively and use the effective thicknesses of $t_{eff}(BL) = 0.95$ nm and $t_{eff}(ML) = 0.475$ nm. See **Figure S18h** and **i** for the fitted dielectric function of the graphene layers.

The 2D Ag phases are modeled with a constant thickness $t_{Ag} = 0.3$ nm, which was estimated from the interlayer distance apparent in cross-sectional TEM. As already discussed, this choice is somewhat arbitrary. Values as high as $t_{Ag} = 2nm$ produce essentially the same dielectric function with the amplitude inversely proportional to the thickness. This explains the consistently higher amplitudes of the dielectric function derived from ellipsometry compared to DFT. The dielectric function was found to be dominated by several absorption peaks, which we describe by (Tauc-) Lorentz dispersion models. The Lorentz dispersion model is the prototypical model used to describe the dielectric response of a semiconductor. The dielectric constant ϵ_L is parameterized as a function of the photon energy E:

$$\epsilon_L(E) = \frac{A_L}{E_L^2 - E^2 - i\Gamma_\mathrm{L} E} \ (3)$$

where A_L, E_L and Γ_L are the oscillator strength, resonance energy and damping constant, respectively.

The Tauc-Lorentz oscillator is a modified Lorentz oscillator model of asymmetric shape for usage near the bandgap of a material.[17] The imaginary part of the dielectric function is defined as:

$$\epsilon_{TL,2}(E) = \begin{cases} 0 & E \leq E_g \\ \frac{\left(E - E_g\right)^2}{E^2} \frac{A_{TL} E_{TL} \Gamma_\mathrm{TL} E}{(E_{TL}^2 - E^2)^2 + \Gamma_{TL}^2 E^2} & E > E_g \end{cases} \ (4)$$

where E_g is the bandgap. The meaning of A_{TL}, E_{TL} and Γ_TL is consistent with the Lorentz oscillator. The real part of the dielectric response $\epsilon_{TL,1}$ is calculated from the above using the Kramers-Kronig relations.[28] The total dielectric function is simply the sum of all oscillators and the high energy limit ϵ_∞:

$$\epsilon = \epsilon_\infty + \sum_i \epsilon_i \ (5)$$

The high energy limit ϵ_∞ represents the dielectric background from high energy contributions outside the spectral measurement range. As there is considerable absorption above our measured spectral range, we expect $\epsilon_\infty > 1$. In our models, the parameter was strongly correlated (>0.95) with the thickness of the adsorption layer t_W. Essentially, due to how thin the layers are, we cannot distinguish between these parameters. Not including any adsorption layer leads to the unphysical result of $\epsilon_\infty < 0$ and is not consistent with our time series of measurements discussed above. To capture the magnitude of the effect, we performed a Uniqueness Test[29], which results in mathematically comparable fits within 10% of the minimum fitting error over the broad range $-5 < \epsilon_\infty < 16$ ($0 < t_W < 2.5$). Therefore, we cannot report a credible value. We set $\epsilon_\infty = 1$, and we do not interpret the absolute magnitude of the dielectric function. However, we want to reiterate that this does not affect the spectral positions of the resonances from the Lorentz- and Tauc-Lorentz oscillators as well as the relative spectral line shape and phase-dependent trends in ε_2, both of which nicely correspond with the DFT calculations.

As can be seen in **Figure S18j** and **k** the dielectric function of the $Ag_{(2)}$ phase extracted from the optical model show some slight variance between the two samples, with a lower dielectric response from sample B. The differences become clearer looking at the parameters tabulated in **Table S2**. While the dominating absorption peak at 2.19 eV is nearly identical, the higher energy, broad absorption peak is less pronounced on sample B. The size of the effect is too large to be explained by uncertainties in the modeling. The observed differences might arise due to the samples having different dominating graphene thicknesses (bilayer EG for $Ag_{(1)}$ predominant vs monolayer EG for $Ag_{(2)}$ predominant samples), which could lead to differing Fermi levels for $Ag_{(2)}$ between the samples.

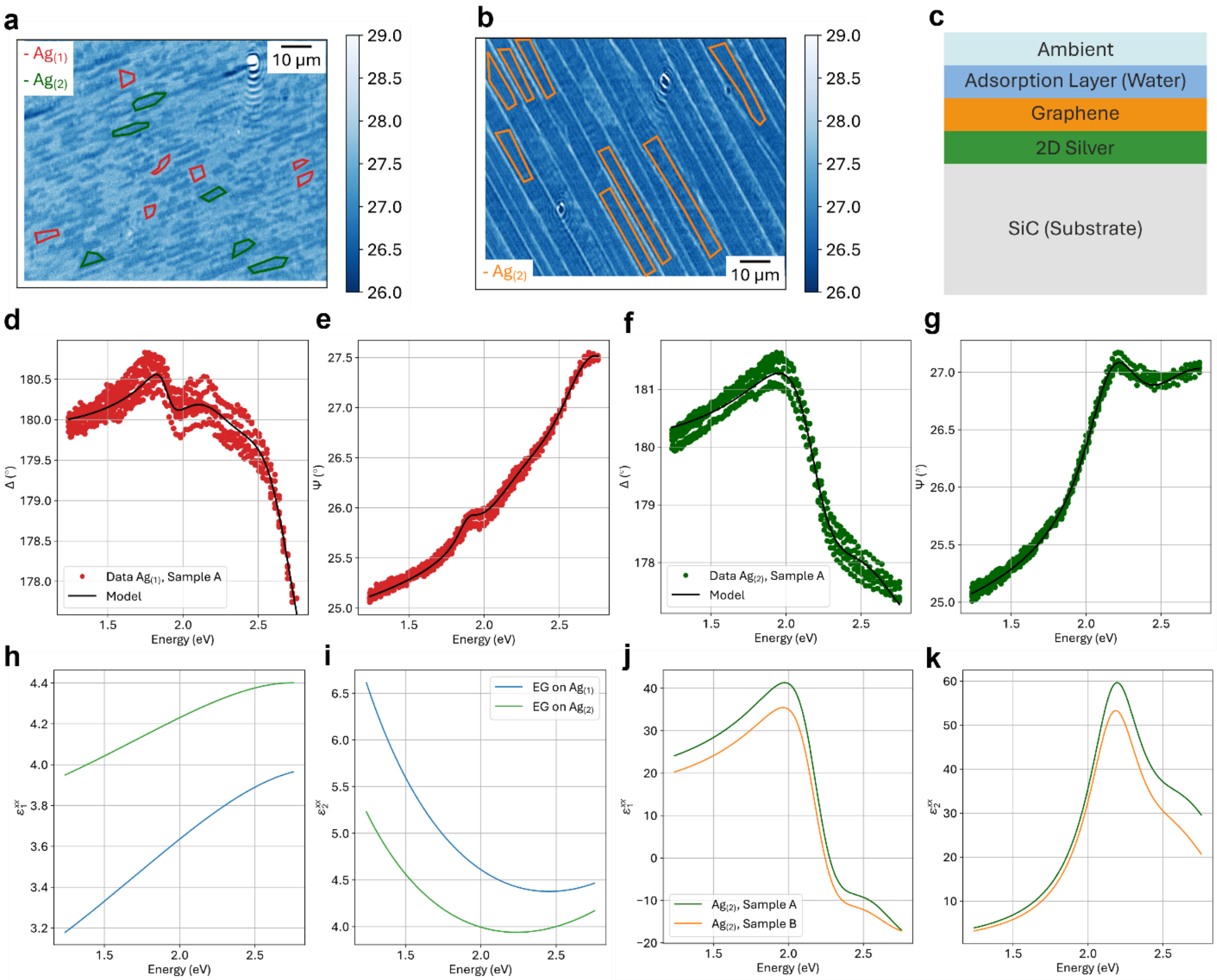

Figure S18. Spectroscopic Imaging ellipsometry (SIE) data analysis for $Ag_{(1)}$ and $Ag_{(2)}$. SIE Ψ-maps taken at 450 nm (~2.76 eV) on the **(a)** $Ag_{(1)}$ (sample A) and **(b)** $Ag_{(2)}$ (sample B) dominant samples, respectively. Regions of interest (ROI) are marked in red ($Ag_{(1)}$), green ($Ag_{(2)}$), and orange ($Ag_{(2)}$). **(c)** Optical model used to extract the dielectric function from SIE data, incorporating the 2D Ag layer, graphene, substrate, and ambient adsorption layer. Corresponding ellipsometric spectra (Ψ(λ), Δ(λ)) for **(d, e)** $Ag_{(1)}$ and **(f, g)** $Ag_{(2)}$, averaged over respective ROIs. **(h)** Real and **(i)** Imaginary parts of the dielectric function ($\varepsilon = \varepsilon_1 + i\,\varepsilon_2$) for EG atop $Ag_{(1)}$ (blue), and $Ag_{(2)}$ (green). **(j)** Real and **(k)** Imaginary parts of the dielectric function for $Ag_{(2)}$, extracted from Sample A (green) and Sample B (orange).

Table S2: Parameters of the dielectric function of $Ag_{(1)}$ and $Ag_{(2)}$

Parameter	sample A, $Ag_{(1)}$	sample A, $Ag_{(2)}$	sample B, $Ag_{(2)}$	Units
ϵ_∞	1	1	1	-
A_{TL}	11.03797	-	-	eV
E_{TL}	1.89207	-	-	eV
Γ_{TL}	0.17293	-	-	eV
E_g	1.15934	-	-	eV
E_L	2.28606	2.19207	2.18533	eV
A_L	21.87463	46.85389	43.9079	eV^2
Γ_L	0.65654	0.43531	0.43865	eV
E_L	2.7165	2.68844	2.63125	eV
A_L	61.52981	53.64585	33.64682	eV^2
Γ_L	0.52531	0.80726	0.72899	eV

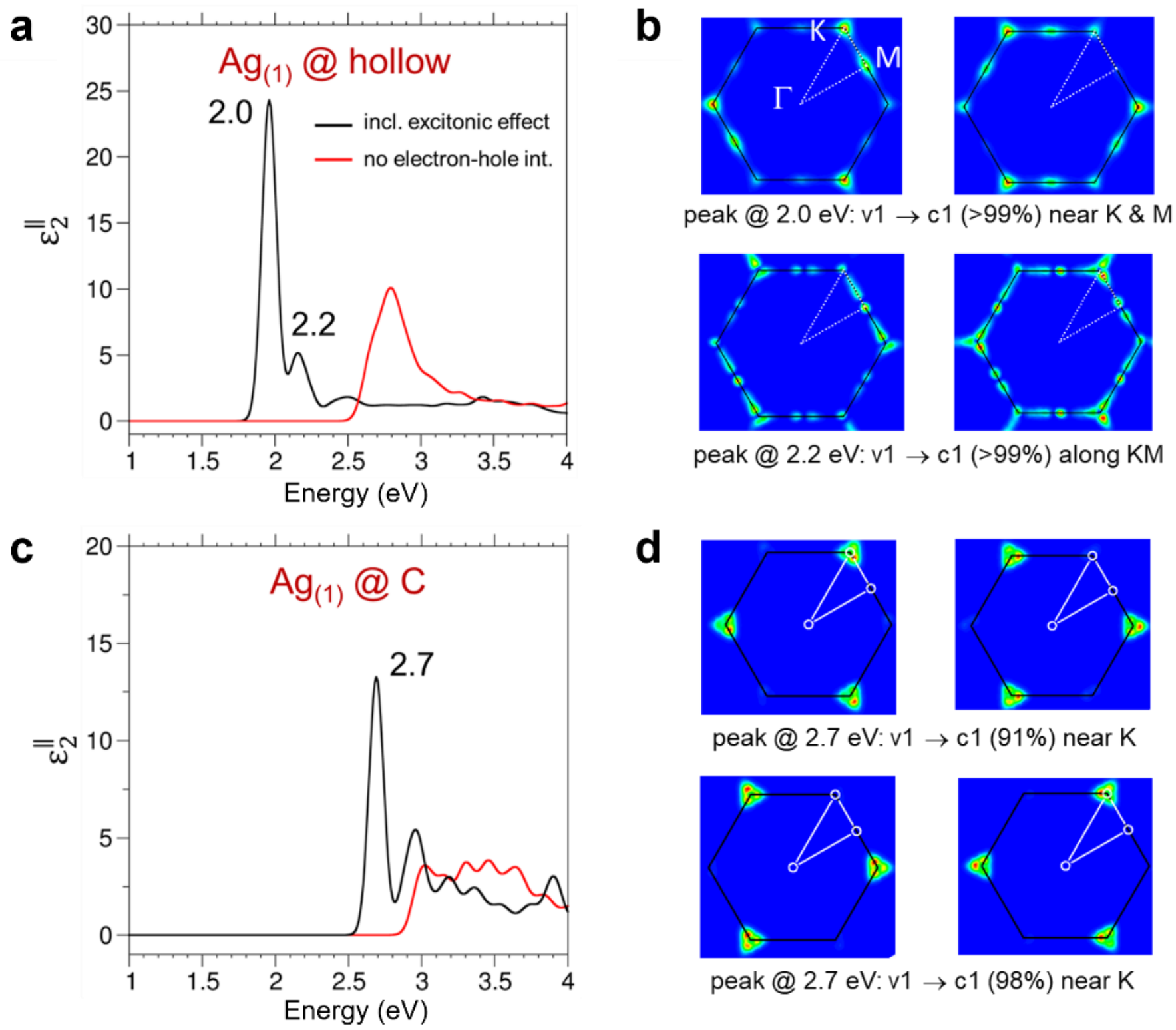

Figure S19. **Optical spectra and k-space distribution of the excitonic transitions for $Ag_{(1)}$.** Computed optical spectra including excitonic effects (GW-BSE) and without electron-hole interactions (GW-IPA) for $Ag_{(1)}$ on (a) hollow site ($Ag_{(1)}$(h)), and (c) carbon site ($Ag_{(1)}$(C)). The k-space distribution transitions leading to (b) excitonic peaks at 2.0 and 2.2 eV for $Ag_{(1)}$(h), and (d) the peak at 2.7 eV for $Ag_{(1)}$(C)). These transitions arise primarily from the topmost valence band to the lowest conduction band.

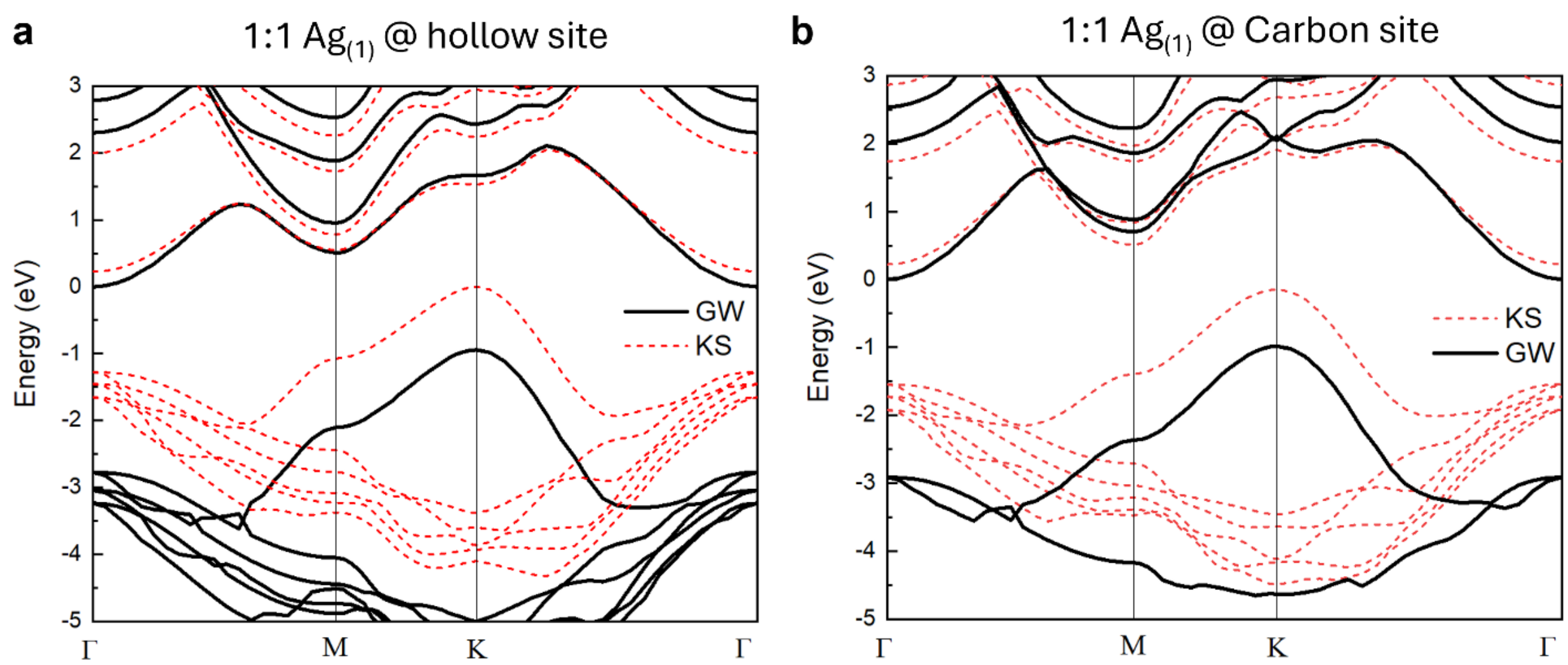

Figure S20. Electronic band structure of $Ag_{(1)}$ on different lattice registries. Electronic band structure for $Ag_{(1)}$ positioned on **(a)** hollow and **(b)** carbon site registries over SiC, calculated using density functional theory (DFT, red dotted lines) and GW (black solid lines) approximations.

Table S3: Calculated bandgaps for $Ag_{(1)}$ on hollow and carbon sites calculated at DFT and GW level (Figure S20)

$Ag_{(1)}$ lattice site	DFT bandgap (eV)		GW bandgap (eV)	
	Indirect	Direct	Indirect	Direct
C	0.37	1.77 (Γ)	0.98	2.91 (Γ)
Hollow	0.23	1.50 (Γ)	0.94	2.60 (K)

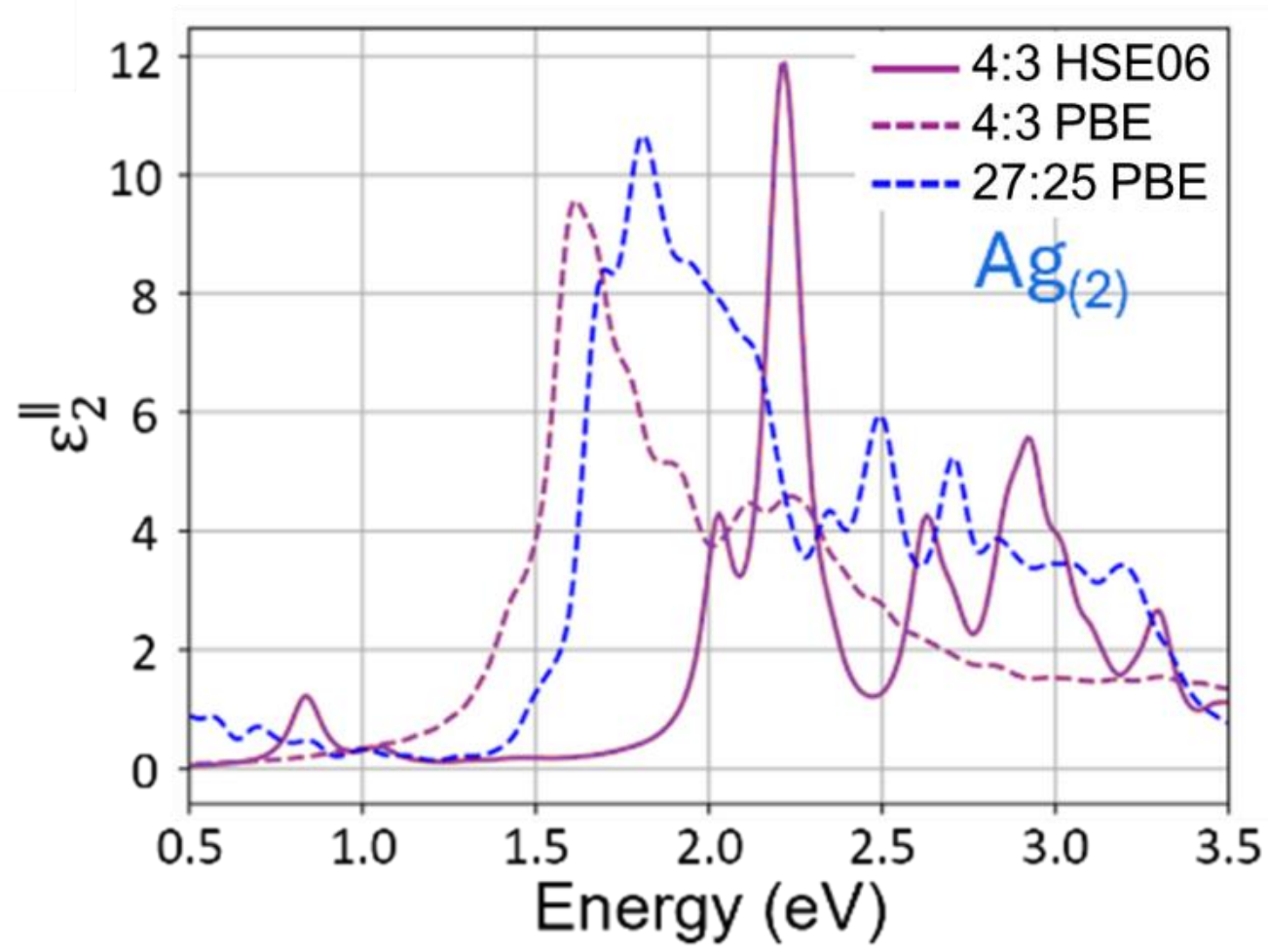

Figure S21. Comparison of ε_2 for $Ag_{(2)}$ using 27:25 and 4:3 Ag:Si models at the PBE level, and 4:3 at the HSE06 level. The spectra calculated at the PBE level using 27:25 and 4:3 Ag:Si models show reasonable agreement.

$\chi^{(2)}$ Analysis from second harmonic generation (SHG) images

The calculation of $\chi^{(2)}$ was done following methods described by Steves *et al.*[30] The total energy of the detected photons per pixel (E_ν) of each SHG image is calculated by

$$E_\nu = \frac{cts}{g} \cdot \frac{S}{QE} \cdot 3.65$$

where cts is the photon count per pixel of the taken SHG image, S is the CCD sensitivity, g is the electron multiplying, QE is the quantum efficiency, and 3.65 is a physical constant accounting for electron generation in Si. In our procedure, S = 4, g = 300, and QE = 0.71. The SHG power is then calculated by $P_{2\omega} = E_\nu/(t \times accu)$ where the collection time t = 10 s and the number of accumulations accu = 40. The sheet susceptibility $\chi^{(2)}_{sheet}$ (nm^2/V) was then calculated following Shen *et al.*[31] and Clark *et al.*[32],

$$|\chi^{(2)}_{sheet}|^2 = \frac{P_{2\omega}(n^{SiC}_\omega + 1)^6 \epsilon_0 c^3 A}{2048\pi^3\omega^2 P^2_\omega}$$

Where ω is the fundamental frequency, n^{SiC}_ω is the refractive index of 6H-SiC at the fundamental frequency adopted from literature,[33] P_ω is the incident laser power at the fundamental frequency, and A is the area illuminated by the exciting pulse. Finally, the effective susceptibility $\chi^{(2)}_{eff}$ (nm/V) is then through the relation $\chi^{(2)}_{eff} = \chi^{(2)}_{sheet}/d$, where d is the Ag monolayer thickness obtained from TEM. Five SHG images were taken for two different $Ag_{(1)}$ samples and two different $Ag_{(2)}$ samples, thus the calculated $\chi^{(2)}$ was averaged over ten ROIs for each Ag phase. The calculated $\chi^{(2)}_{sheet}$ for $Ag_{(1)}$ and $Ag_{(2)}$ are 0.001 ± 0.0001 and 3.2 ± 0.08 nm^2/V, respectively. And $\chi^{(2)}_{eff}$ for $Ag_{(1)}$ and $Ag_{(2)}$ are 0.004 ± 0.0005 and 11.4 ± 0.3 nm/V, respectively.

Extension of Defect-Templated Phase Engineering to Confined 2D Gallium

To investigate whether defect-templated phase engineering extends beyond Ag, we performed preliminary growth experiments using confined two-dimensional Ga. Confined Ga is known to form two structurally distinct phases: $Ga_{(1)}$, comprising two atomic Ga layers, and $Ga_{(2)}$, comprising three atomic Ga layers. These phases can be distinguished through their characteristic ultra-low-frequency Raman modes.[8]

Under conventional CHet growth conditions using oxygen-plasma-treated epitaxial graphene, the resulting samples exhibited mixed-phase growth or predominantly $Ga_{(1)}$, as shown by the optical image and corresponding ultra-low-frequency Raman spectrum in **Figure S22**. In contrast, growth using zero-layer graphene as the starting template preferentially produced the $Ga_{(2)}$ phase. This observation is consistent with previous demonstration of $Ga_{(2)}$ growth beneath zero-layer graphene and suggests that the bonding configuration and defect chemistry of the graphene template influence Ga phase selection during intercalation.[34]

The Ga results parallel the template-dependent behavior observed for Ag, although the selected structures differ in thickness rather than only lateral atomic packing. Together, these observations support a broader framework in which graphene defects and interfacial bonding configurations modify intercalant access, nucleation kinetics, and the relative accessibility of competing confined-metal phases.

The approach is expected to be most applicable to metals that possess multiple structural configurations with comparable formation energies but distinct nucleation barriers or interfacial bonding preferences. Its transferability may be limited when one structure is overwhelmingly thermodynamically favored, when the intercalant forms stable silicides, carbides, or three-dimensional clusters, or when the defect treatment compromises the integrity of the graphene confinement layer. Consequently, the optimal defect type, density, and chemical termination are expected to remain specific to each metal–substrate system.

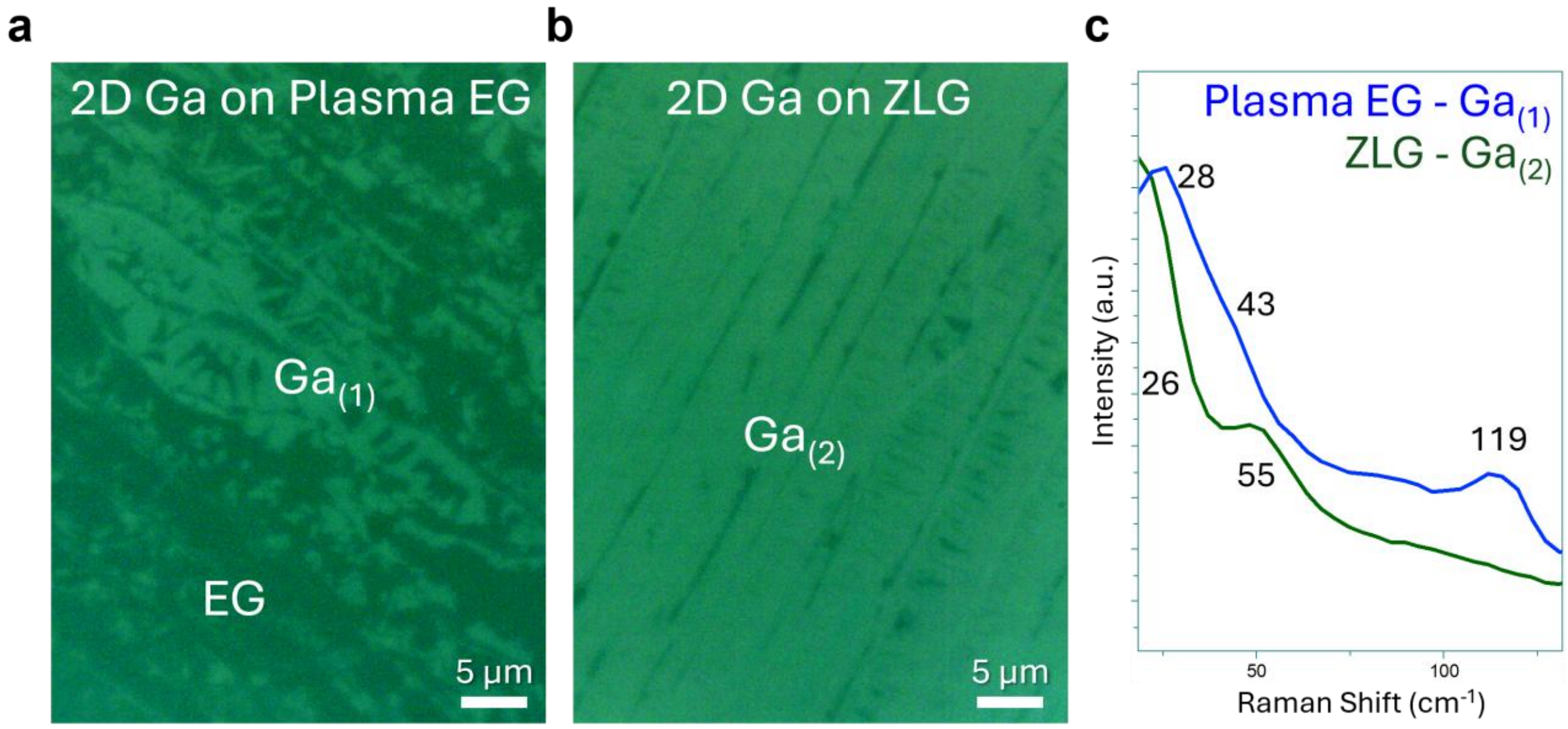

Figure S22. Extension of defect-templated phase engineering to confined 2D gallium. (a, b) Optical micrographs of 2D Ga grown on oxygen plasma-treated epitaxial graphene and zero-layer graphene, respectively. (c) Corresponding ultra-low-frequency Raman spectra showing preferential formation of $Ga_{(1)}$ on plasma-treated EG and $Ga_{(2)}$ on ZLG. Characteristic ULF Raman modes of the two phases are labelled. These results suggest that graphene defect chemistry can influence phase selection in confined metals beyond Ag.

Supplementary References

1. Briggs, N. *et al.* Atomically thin half-van der Waals metals enabled by confinement heteroepitaxy. *Nat. Mater.* **19**, 637–643 (2020).
2. Riedl, C. Quasi-Free-Standing Epitaxial Graphene on SiC Obtained by Hydrogen Intercalation. *Phys. Rev. Lett.* **103**, (2009).
3. Vera, A. *et al.* Large-Area Intercalated Two-Dimensional Pb/Graphene Heterostructure as a Platform for Generating Spin–Orbit Torque. *ACS Nano* **18**, 21985–21997 (2024).
4. Moulder, J. F. *Handbook of X-Ray Photoelectron Spectroscopy: A Reference Book of Standard Spectra for Identification and Interpretation of XPS Data*. (Physical Electronics Division, Perkin-Elmer Corporation, 1992).
5. Turker, F. *et al.* 2D Oxides Realized via Confinement Heteroepitaxy. *Advanced Functional Materials* **33**, 2210404 (2023).
6. Rosenzweig, P. & Starke, U. Large-area synthesis of a semiconducting silver monolayer via intercalation of epitaxial graphene. *Phys. Rev. B* **101**, 201407 (2020).
7. Wang, Y. & Crespi, V. H. Atlas of 2D metals epitaxial to SiC: filling-controlled gapping conditions and alloying rules. Preprint at https://doi.org/10.48550/arXiv.2011.01914 (2020).
8. Wetherington, M. T. *et al.* 2-dimensional polar metals: a low-frequency Raman scattering study. *2D Mater.* **8**, 041003 (2021).
9. Zhang, K. *et al.* Tunable phononic quantum interference induced by two-dimensional metals. *Sci Adv* **11**, eadw1800 (2025).

10. Briggs, N. *et al.* Epitaxial graphene/silicon carbide intercalation: a minireview on graphene modulation and unique 2D materials. *Nanoscale* **11**, 15440–15447 (2019).

11. Robinson, J. A. *et al.* Correlating Raman Spectral Signatures with Carrier Mobility in Epitaxial Graphene: A Guide to Achieving High Mobility on the Wafer Scale. *Nano Lett.* **9**, 2873–2876 (2009).

12. Lee, J. E., Ahn, G., Shim, J., Lee, Y. S. & Ryu, S. Optical separation of mechanical strain from charge doping in graphene. *Nat Commun* **3**, 1024 (2012).

13. Volotsenko, I. *et al.* Secondary electron doping contrast: Theory based on scanning electron microscope and Kelvin probe force microscopy measurements. *J. Appl. Phys.* **107**, (2010).

14. Rosenzweig, P., Karakachian, H., Marchenko, D. & Starke, U. Surface charge-transfer doping a quantum-confined silver monolayer beneath epitaxial graphene. *Phys. Rev. B* **105**, 235428 (2022).

15. Lee, W. *et al.* Confined Monolayer Ag As a Large Gap 2D Semiconductor and Its Momentum Resolved Excited States. *Nano Lett.* **22**, 7841–7847 (2022).

16. Wang, S. *et al.* 4H-SiC: a new nonlinear material for midinfrared lasers. *Laser & Photonics Reviews* **7**, 831–838 (2013).

17. Fujiwara, H. *Spectroscopic Ellipsometry: Principles and Applications*. (John Wiley & Sons, 2007).

18. Schaper, S., Duwe, M. & Wurstbauer, U. Unambiguous determination of optical constants and thickness of ultrathin films by using optical anisotropic substrates. Preprint at https://doi.org/10.48550/arXiv.2502.21241 (2025).

19. Majérus, B., Dremetsika, E., Lobet, M., Henrard, L. & Kockaert, P. Electrodynamic models of 2D materials: can we match thin film and single sheet approaches? *Phys. Rev. B* **98**, 125419 (2018).

20. Yoo, S. & Park, Q. Spectroscopic ellipsometry for low-dimensional materials and heterostructures. *Nanophotonics* **11**, 2811–2825 (2022).

21. Sigger, F. *et al.* Spectroscopic imaging ellipsometry of two-dimensional TMDC heterostructures. *Applied Physics Letters* **121**, 071102 (2022).

22. Nguyen, H. T. *et al.* Temperature Dependence of Optical Properties of MoS2 and WS2 Heterostructures Assessed by Spectroscopic Ellipsometry. *Nanomaterials* **15**, 76 (2025).

23. Nisi, K. *et al.* Light–Matter Interaction in Quantum Confined 2D Polar Metals. *Advanced Functional Materials* **31**, 2005977 (2021).

24. Majérus, B., Guillaume, E., Kockaert, P. & Henrard, L. Anisotropy and effective medium approach in the optical response of 2D material heterostructures. Preprint at https://doi.org/10.48550/arXiv.2309.14899 (2023).

25. Ochoa-Martínez, E. *et al.* Determination of a refractive index and an extinction coefficient of standard production of CVD-graphene. *Nanoscale* **7**, 1491–1500 (2015).

26. Okano, S. *et al.* Voltage-Controlled Dielectric Function of Bilayer Graphene. *Advanced Optical Materials* **8**, 2000861 (2020).

27. Tikuišis, K. K. Dielectric function of epitaxial quasi-freestanding monolayer graphene on Si-face 6H-SiC in a broad spectral range. *Phys. Rev. Mater.* **7**, (2023).

28. Jellison, G. E., Jr. & Modine, F. A. Parameterization of the optical functions of amorphous materials in the interband region. *Appl. Phys. Lett.* **69**, 371–373 (1996).

29. Hilfiker, J. N. *et al.* Survey of methods to characterize thin absorbing films with Spectroscopic Ellipsometry. *Thin Solid Films* **516**, 7979–7989 (2008).

30. Steves, M. A. *et al.* Unexpected Near-Infrared to Visible Nonlinear Optical Properties from 2-D Polar Metals. *Nano Lett.* **20**, 8312–8318 (2020).

31. Shen, Y. R. Optical Second Harmonic Generation at Interfaces. *Annual Review of Physical Chemistry* **40**, 327–350 (1989).

32. Clark, D. J. *et al.* Strong optical nonlinearity of CVD-grown MoS 2 monolayer as probed by wavelength-dependent second-harmonic generation. *Phys. Rev. B* **90**, 121409 (2014).

33. Ninomiya, S. & Adachi, S. Optical Constants of 6H SiC Single Crystals. *Japanese Journal of Applied Physics* **33**, 2479 (1994).

34. Yi, H. *et al.* Orbital-hybridization-induced Ising-type superconductivity in a confined gallium layer. *Nat. Mater.* 1–7 (2026) doi:10.1038/s41563-026-02573-y.
